# Addition of Amine-Substituted Heteroaromatic Rings to Alkynyl Bridging Ligands to Generate Phosphorescent Emitters Incorporating Iridaimidazoles with Fused Heterocyclic Rings

**DOI:** 10.1021/acs.inorgchem.5c04705

**Published:** 2025-12-24

**Authors:** Cristina Martín-Escura, Enrique Oñate, Montserrat Oliván, Ana M. López

**Affiliations:** Departamento de Química Inorgánica, Instituto de Síntesis Química y Catálisis Homogénea (ISQCH), Centro de Innovación en Química Avanzada (ORFEO-CINQA), Universidad de Zaragoza—CSIC, 50009 Zaragoza, Spain

## Abstract

Alkynyl ligands are versatile building blocks in the design of luminescent iridium­(III) complexes due to their ability to support postcoordination functionalization that gives rise to ligands non available through conventional coordination chemistry. Here, we report the synthesis and characterization of a new family of heteroleptic iridium­(III) green emitters based on cyclometalated 2-*p*-tolylpyridine as main ligands and new *C*,*N*-chelating units as the auxiliary ligand that yield iridaimidazole structures. The alkynyl bridging dimer *cis*-[Ir­(μ-C≡C^*t*^Bu)­{κ^2^-*C*,*N*-(MeC_6_H_3_-py)}_2_]_2_ (**1**) reacts with amine-substituted five-membered heterocycles bearing two heteroatoms, such as 1-methyl-1*H*-imidazol-2-amine, 1-methyl-1*H*-benzo­[*d*]­imidazol-2-amine, 4-methyloxazol-2-amine, benzo­[*d*]­oxazol-2-amine, 4-methylthiazol-2-amine, and benzo­[*d*]­thiazol-2-amine, giving the iridaimidazole derivatives Ir­{κ^2^-*C*,*N*-(MeC_6_H_3_-py)}_2_{κ^2^-*C*,*N*-[C­(CH_2_^*t*^Bu)­N-im]} (im = imidazole, **2**), Ir­{κ^2^-*C*,*N*-(MeC_6_H_3_-py)}_2_{κ^2^-*C*,*N*-[C­(CH_2_^*t*^Bu)­N-bzim]} (bzim = benzimidazole, **3**), Ir­{κ^2^-*C*,*N*-(MeC_6_H_3_-py)}_2_{κ^2^-*C*,*N*-[C­(CH_2_^*t*^Bu)­N-oxazol]} (**4**), Ir­{κ^2^-*C*,*N*-(MeC_6_H_3_-py)}_2_{κ^2^-*C*,*N*-[C­(CH_2_^*t*^Bu)­N-bzoxazol]} (**5**), Ir­{κ^2^-*C*,*N*-(MeC_6_H_3_-py)}_2_{κ^2^-*C*,*N*-[C­(CH_2_^*t*^Bu)­N-thiazol]} (**6**), and Ir­{κ^2^-*C*,*N*-(MeC_6_H_3_-py)}_2_{κ^2^-*C*,*N*-[C­(CH_2_^*t*^Bu)­N-bzthiazol]} (**7**), respectively. The iridium center in these complexes is in an octahedral environment with nitrogen and carbon atoms in a facial disposition. Complexes **2**, **4**, and **6** feature one five-membered heteroaromatic ring fused to the iridaimidazole cycle whereas **3**, **5**, and **7** additionally contain a benzo group fused to the organic heterocycle. All complexes are green phosphorescent emitters (474**–**558 nm) upon photoexcitation, with high quantum yields (0.55**–**0.92) in poly­(methyl methacrylate) films and 2-MeTHF at 298 K.

## Graphical abstract

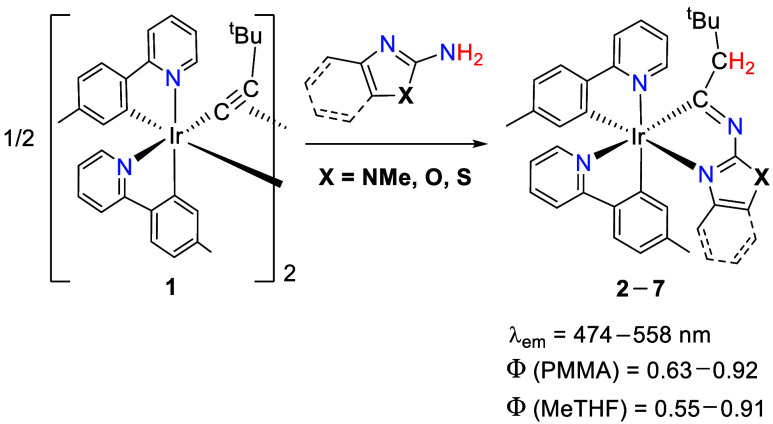

## Introduction

The development of new phosphorescent emitters based on iridium­(III) complexes is pursued mainly for two reasons. First, due to the large spin–orbit coupling constant of the metal ion, these type of complexes exhibit a fast intersystem crossing between the singlet S_1_ and triplet T_1_ excited states, allowing them to achieve internal quantum efficiencies approaching 100%.^1^ Second, their photophysical properties depend on the combination of ligands around the iridium­(III) center, which allows their emissions to be tuned by careful selection of these.^2^ The most common structural design involves octahedral coordination spheres formed by three bidentate 3-electron donor ligands (3b), often with two or all three different. Notably, heteroleptic complexes bearing three different ligands, such as [3b + 3b′ + 3b″], allow for more precise adjustment of the photophysical properties. However, they are difficult to prepare with good yields and usually present serious issues associated with ligand distribution equilibria.^3^ In order to avoid these problems, heteroleptic emitters bearing two different types of ligands ([3b + 3b + 3b′]) are particularly valued.^4^ Among heteroleptic emitters, it is well established that the ancillary 3b′ ligand can play a key role in tuning emission wavelength and modulating both photophysical and electrochemical behaviors.^5^ Most designs contain two 3b cyclometalated aryl-pyridine-type ligands, typically with *N*,*N*-*trans* configurations. The stereochemistry of homoleptic tris­(bidentate) iridium­(III) emitters, particularly the relative orientation of the donor atoms, has a marked impact on their electronic structure and photophysical behavior.^6^ Thus, facial (*fac*) and meridional (*mer*) isomers exhibit different redox properties and emission profiles: *mer* isomers typically show lower quantum yields, red-shifted emissions and shorter excited-state lifetimes compared to their *fac* counterparts, which tend to exhibit higher efficiencies and better color puritity.^7^ Much less studied has been the effect of the *cis*- or *trans*- arrangement of the heteroatoms of the bidentate ligands 3b in heteroleptic [3b + 3b + 3b′] iridium­(III) emitters,^8^ since most of them present a *trans* arrangement. Traditionally, ancillary 3b′ ligands are introduced via metathesis or ligand substitution reactions, beginning from an appropriate bis-cyclometalated iridium precursor.^2,4a,4c,4d^ While these methods are well established and broadly applicable, they inherently restrict the range of accessible ligands to those that can be isolated as stable free ligands or proligands. To overcome this limitation, an emergent approach for the preparation of cyclometalated [3b + 3b + 3b′] iridium­(III) emitters involves postfunctionalization of some coordinated ligands. This strategy enables the incorporation of ancillary ligands that are otherwise inaccessible through conventional synthetic routes.^9^

Acetylide anions are interesting auxiliary ligands for the design of transition metal phosphorescent emitters. For instance, in platinum­(II) and gold­(III) complexes, these strong σ-donating and strong-field ligands increase the energy gap between the lowest-lying excited state and the d-d state, which optimize their photoluminescence properties.^10^ Furthermore, the coordination of an acetylide ligand to a middle/late transition metal center, profoundly alters the electronic structure and reactivity of the triple bond.^11^ Specifically, binding of the alkynyl unit to the metal center shifts nucleophilicity from the C_α_ atom toward the C_β_ atom, enhancing susceptibility to electrophilic attack at C_β_ and enabling nucleophilic addition at C_α_.^12^ This significantly broadens the reactivity of the carbon–carbon triple bond and facilitates its postcoordination functionalization, offering access to structurally diverse systems with potential applications in materials science, photophysics, and catalysis.^11b,13^

Recently, our research group has reported the preparation of phosphorescent iridium­(III) emitters containing iridaimidazo­[1,2-*a*]­pyridine^14^ and iridaoxazole^15^ units (Scheme 1) using alkynyl ligands as building blocks. These complexes were synthesized via the alkynyl bridging dinuclear complex *cis*-[Ir­(μ-C≡C*^t^* Bu)­{κ^2^-*C*,*N*-(MeC_6_H_3_-py)}_2_]_2_ (**1**), which unlike the usual chloride bridging dimers [Ir­(*μ-*Cl)­(3b)_2_]_2_, has a *cis* disposition of the heterocycles of the 3b ligands.

**Scheme 1:**
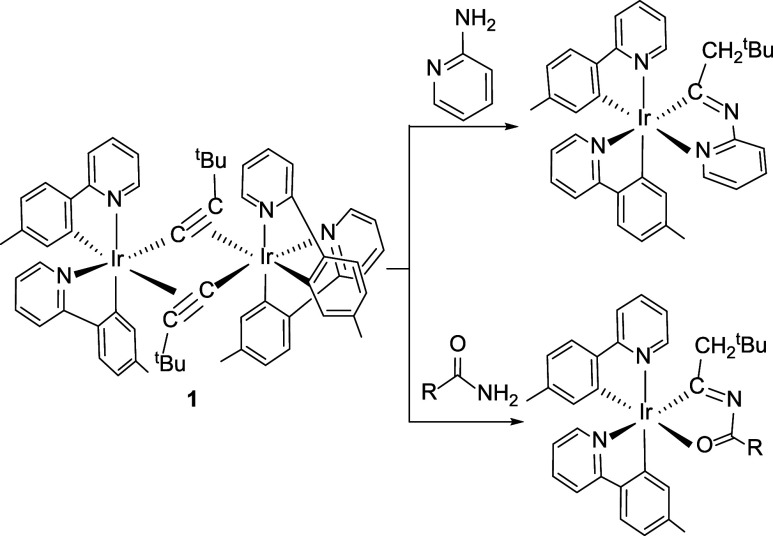
Preparation of Iridaimidazopyridine and Iridaoxazole Emitters

This work is a further demonstration that alkynyl ligands are useful building blocks in organometallic chemistry, leading to the formation of novel ligands in the metal coordination sphere. Here we show that the reactions between the alkynyl bridging dimer *cis*-[Ir­(μ-C≡C*^t^* Bu)­{κ^2^-*C*,*N*-(MeC_6_H_3_-py)}_2_]_2_ (**1**) and amine-substituted five-membered heteroaromatic rings bearing two heteroatoms (Chart 1) give rise to a new family of iridium­(III) green emitters of the type [3b + 3b + 3b′] containing the heteroatoms of the 3b ligands mutually *cis* disposed. They are iridaimidazoles with fused heterocyclic rings including *N*-methylimidazole, *N*-methylbenzimidazole, oxazole, benzoxazole, thiazole, and benzothiazole, which broadens the structural diversity of phosphorescent iridium­(III) complexes.

**Chart 1:**
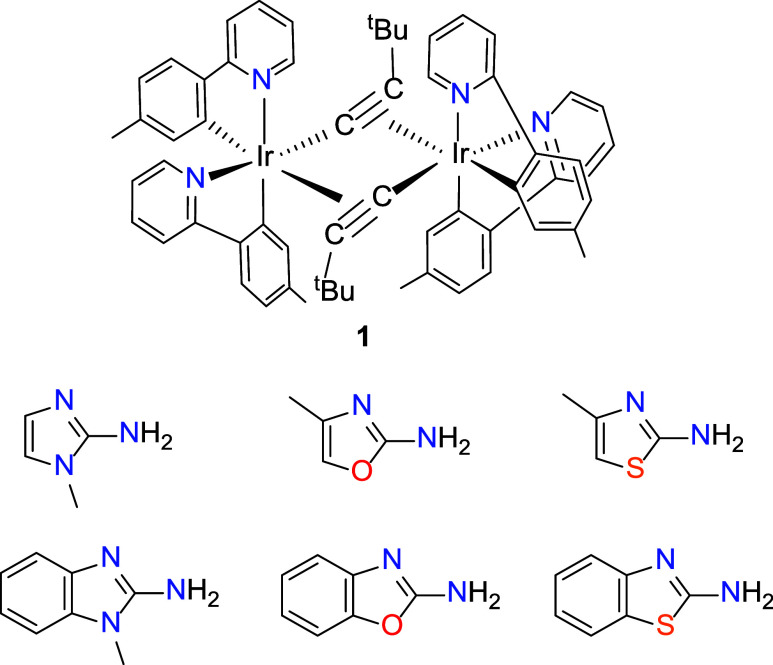
Alkynyl Iridium­(III) Complex and Amine-Substituted Heterocycles Used in This Work

## Results and Discussion

### Preparation of Iridaimidazole Derivatives with Fused Heterocycles

The treatment of a toluene suspension of the alkynyl dimer **1** with 1.0 equiv of 1-methyl-1*H*-imidazol-2-amine hydrochloride and 1.0 equiv of triethylamine, at 120 °C for 24 h, gives the mononuclear complex Ir­{κ^2^-*C*,*N*-(MeC_6_H_3_-py)}_2_{κ^2^-*C*,*N*-[C­(CH_2_^*t*^Bu)­N-im]} (**2**), which was isolated as a yellow solid in moderate yield (35%) after precipitation in pentane (Scheme 2). According to the reactions shown in Scheme 1, its formation is the result of the breaking of the alkynyl bridges of the precursor and the formal addition of the amino group to the triple bond together with the coordination of the unsubstituted nitrogen atom of the imidazole to the iridium center. The introduction of a benzo unit fused to the imidazole group extends the electronic delocalization, which could modify the photophysical properties of the resulting iridium­(III) complexes. This prompted us to carry out the reaction of dimer **1** with 1-methyl-1*H*-benzo­[*d*]­imidazol-2-amine. In toluene at 120 °C, after 24 h, the reaction afforded Ir­{κ^2^-*C*,*N*-(MeC_6_H_3_-py)}_2_{κ^2^-*C*,*N*-[C­(CH_2_^*t*^Bu)­N-bzim]} (**3**), which was isolated also as a yellow solid, in 40% yield.

**Scheme 2:**
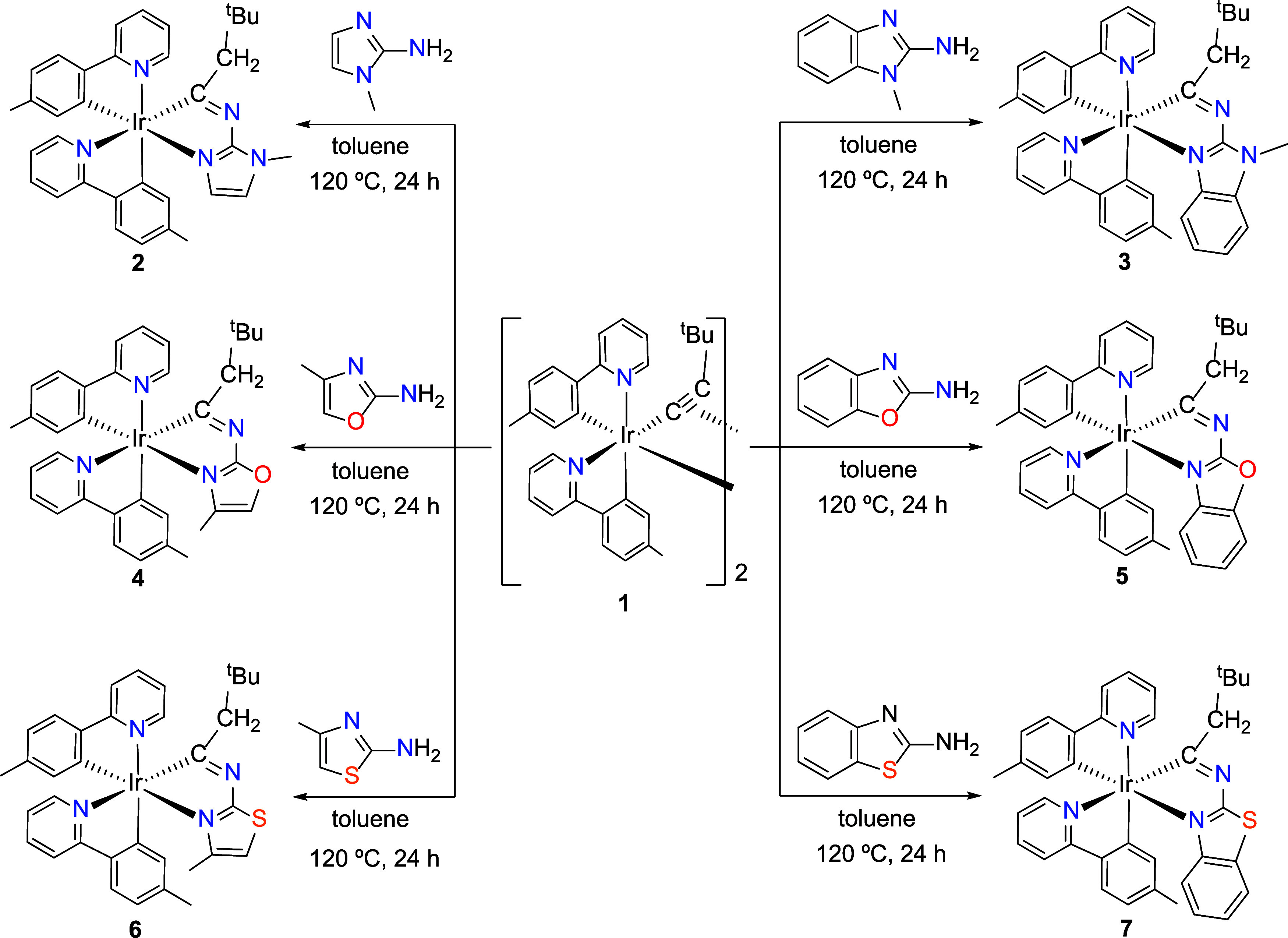
Synthesis of Complexes **2–7**

Complex **3** was characterized by X-ray diffraction analysis. Its molecular structure, shown in Figure 1, verifies the formation of an iridaimidazo­[1,2-*a*]­benzo­[*d*]­imidazole tricyclic system. The octahedral coordination sphere around the iridium center is completed by the two orthometalated 2-(*p*-tolyl)­pyridine ligands with the nitrogen atoms mutually *cis* disposed, and also *cis* to the other coordinated nitrogen, so that the three coordinated carbon atoms and the three coordinated nitrogen ones are facially arranged. Within the five-membered iridaimidazole ring, the bond lengths Ir–C(1), C(1)–N(2), N(2)–C(7), and C(7)–N(1) are 1.9948(18), 1.326(2), 1.363(2), and 1.330(2) Å, respectively, values intermediate between typical single and double bonds, indicating delocalized bonding through these atoms. These distances are comparable with those found for the related iridaimidazo­[1,2-*a*]­pyridine derivative Ir­{κ^2^-*C*,*N*-(C_6_H_4_-isoqui)}_2_{κ^2^-*C*,*N*-[C­(CH_2_Ph)­N-py]} (isoqui = isoquinoline).^14^ The iridaimidazole ring is planar, with the C(1) carbon atom deviating the most, 0.033(1) Å, from the ideal plane formed by the five atoms. Despite the observed bond distances and planarity, the iridaimidazole ring displays minimal aromatic character. Thus, the computed nuclear-independent chemical shift values at the ring center, NICS (0), and 1 Å above, NICS (1), and below the plane, NICS (−1), are 0.99, −0.63, and −1.23, respectively. For comparison, the corresponding NICS values for the five- and six-membered rings of the fused benzimidazole unit are much more negative, −9.60, −7.65, and −8.07 for the imidazole ring, and −11.25, −11.32, and −11.61 for the benzo moiety, as expected for truly aromatic rings. This is further supported by the anisotropy of the induced current density analysis (AICD),^16^ which shows no evidence of global diatropic current in the iridacycle but shows clear clockwise current density vectors in the benzimidazole unit (Figure S14b). Similarly, NICS (0, 1, and −1) values (0.83, −0.75, and −1.23) and AICD plot for the iridaimidazole unit of complex **2** (Figure S14a) are consistent with the lack of aromaticity of that five-membered ring in this compound.

**Figure 1:**
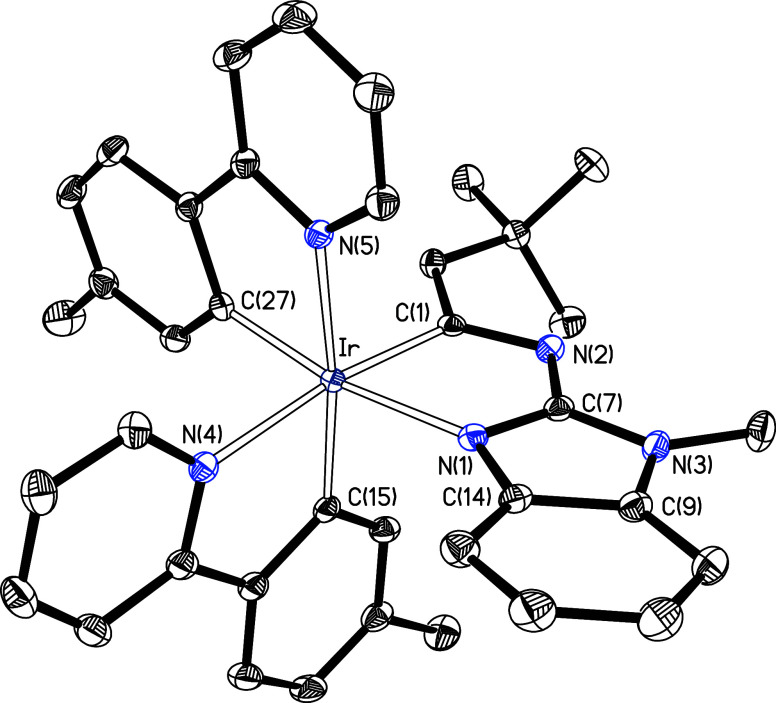
X-ray molecular structure of complex **3** (50% probability ellipsoids; hydrogen atoms have been omitted). Selected bond lengths (Å) and angles (deg): Ir–N(1) = 2.1352(15), Ir–N(4) = 2.1420(16), Ir–N(5) = 2.1243(16), Ir–C(1) = 1.9948(18), Ir–C(15) = 2.0113(19), Ir–C(27) = 2.0078(19), C(1)–N(2) = 1.326(2), N(2)–C(7) = 1.363(2), C(7)–N(1) = 1.330(2); C(1)–Ir–N(4) = 171.18(6), C(15)–Ir–N(5) = 173.28(6), C(27)–Ir–N(1) = 170.17(7), C(1)–Ir–N(1) = 76.94(7), C(15)–Ir–N(4) = 79.25(7), C(27)–Ir–N(5) = 79.95(7).

The ^1^H NMR spectra of complexes **2** and **3** (CD_2_Cl_2_, 298 K) agree with the X-ray structure of **3**. The most characteristic signal of these spectra is an AB spin system centered at 2.47 ppm, characterized by *J*_A–B_ coupling constants of ∼16 Hz and Δν values of 35.0 (**2**) and 19.0 (**3**) Hz, corresponding to the methylene protons of the neopentyl substituent of the newly generated *C*,*N*-bidentate ligand. The ^13^C­{^1^H} NMR spectra display the matching methylene carbon signal at around 60 ppm. Additionally, they contain a low-field resonance due to the metalated carbon atom of the Ir–C–N unit at 227.7 (**2**) and 239.7 (**3**) ppm, which support a significant double bond character for the Ir–C bond of the iridaimidazole.

Encouraged by these results, we decided to explore the reactivity of the alkynyl complex **1** with other amine-substituted five-membered heterocycles of oxazole and thiazole types (Chart 1). Heating of toluene suspensions of dimer **1** with one mol of the corresponding amine (4-methyloxazol-2-amine, benzo­[*d*]­oxazol-2-amine, 4-methylthiazol-2-amine, and benzo­[*d*]­thiazol-2-amine) per iridium mol, at 120 °C for 24 h, afforded the mononuclear derivatives Ir­{κ^2^-*C*,*N*-(MeC_6_H_3_-py)}_2_{κ^2^-*C*,*N*-[C­(CH_2_^*t*^Bu)­N-oxazol]} (**4**), Ir­{κ^2^-*C*,*N*-(MeC_6_H_3_-py)}_2_{κ^2^-*C*,*N*-[C­(CH_2_^*t*^Bu)­N-bzoxazol]} (**5**), Ir­{κ^2^-*C*,*N*-(MeC_6_H_3_-py)}_2_{κ^2^-*C*,*N*-[C­(CH_2_^*t*^Bu)­N-thiazol]} (**6**), and Ir­{κ^2^-*C*,*N*-(MeC_6_H_3_-py)}_2_{κ^2^-*C*,*N*-[C­(CH_2_^*t*^Bu)­N-bzthiazol]} (**7**), respectively (Scheme 2). Complexes **4**–**7** were isolated as yellow solids in moderate yields (34–46%). Complexes **4** and **6** were structurally characterized by single-crystal X-ray diffraction analysis. Figure 2a displays the structure of one of the two independent molecules of the oxazol-derived complex **4**, which are present in the asymmetric unit, while Figure 2b contains the structure of the thiazole derivative **6**. Both structures demonstrate that, similarly to the formation of **2** and **3**, the nitrogen atom of the amine group has added to the C_α_ atom of the alkynyl ligand of **1** whereas both amino hydrogen atoms have gone to the C_β_ atom. Furthermore, in both cases, of the two heteroatoms present in the five membered organic heterocycle, the nitrogen atom coordinates selectively to the iridium­(III) center giving rise to an iridaimidazole structure, common to complexes **2**–**7**. As in the case of **3**, the formation of **4** and **6** also takes place with retention of the stereochemistry of the starting dimer **1**, i.e., the relative *cis* arrangement of the nitrogen atoms of the *p*-tolylpyridine ligands, N(3) and N(4), is maintained. The coordinated nitrogen atom N(1) of the generated chelate ligands is also in a *cis* arrangement with respect to the other two nitrogen atoms coordinated to the metal center. The new iridaimidazole units in **4** and **6** are planar, with atoms C(7) in **4** and N(2) in **6** showing the greatest deviation (0.042(9) and 0.0004(13) Å) from the ideal plane. The bond lengths of the sequence Ir–C(1)–N(2)–C(7)–N(1) are very similar to those of **3**, suggesting some electron delocalization between these atoms. However, the NICS values (Table S1) and AICD diagrams (Figure S14c–f) of **4**–**7** indicate that the iridaimidazole ring of these compounds, like that of **2** and **3**, is also not aromatic.

**Figure 2:**
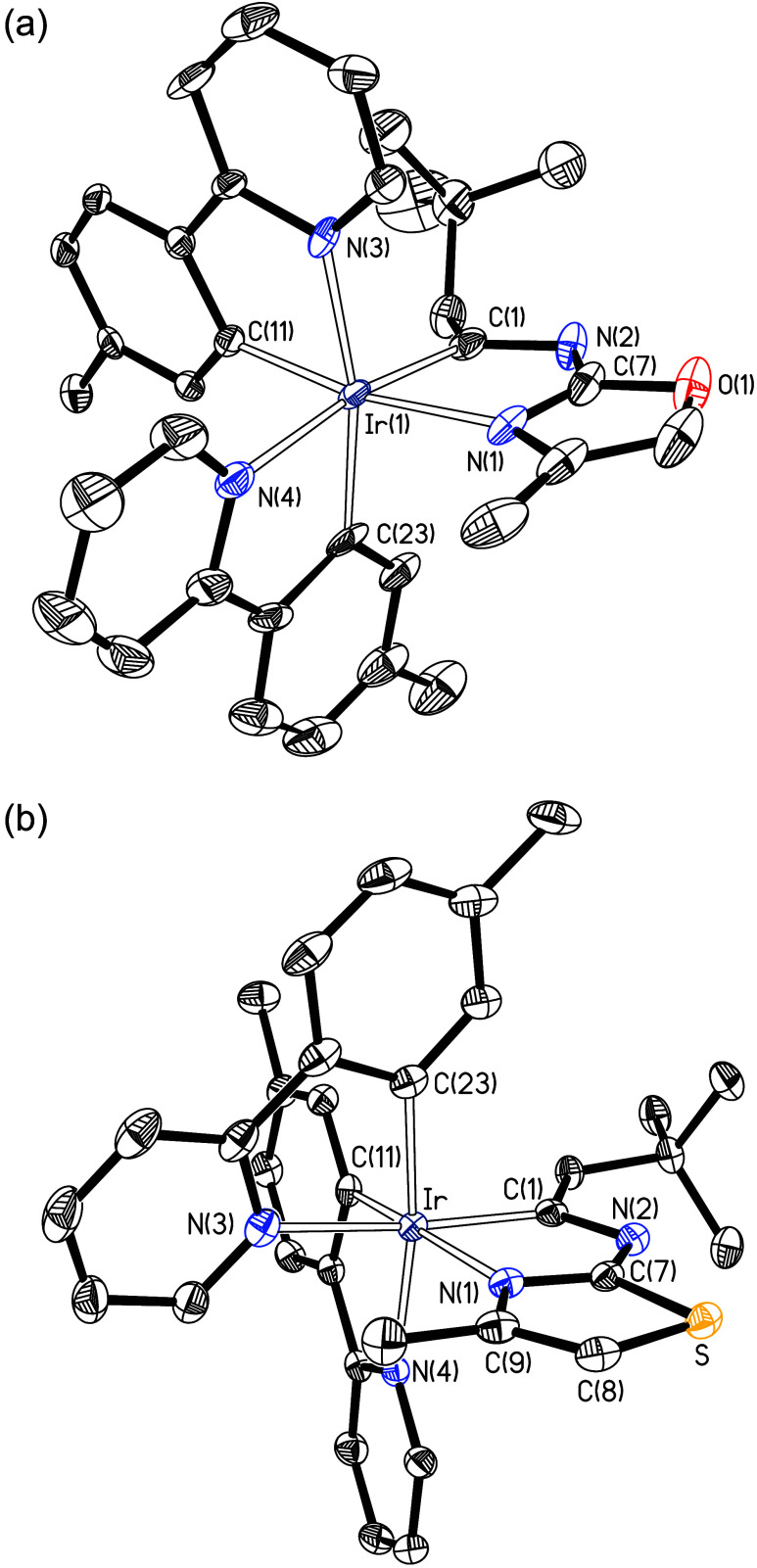
(a) X-ray molecular structure of one of the two independent molecules of complex **4** (50% probability ellipsoids; hydrogen atoms have been omitted). Selected bond lengths (Å) and angles (deg) for the two molecules in the asymmetric unit: Ir(1)–N(1) = 2.145(5), 2.117(5), Ir(1)–N(3) = 2.111(5), 2.134(5), Ir(1)–N(4) = 2.141(5), 2.074(4), Ir(1)–C(1) = 1.998(6), 2.004(6), Ir(1)–C(11) = 2.010(6), 2.017(5), Ir(1)–C(23) = 2.008(6), 2.114(5), C(1)–N(2) = 1.336(7), 1.319(8), N(2)–C(7) = 1.357(8), 1.356(8), C(7)–N(1) = 1.300(8), 1.314(9); C(1)–Ir(1)–N(4) = 170.63(19), 162.2(2), C(11)–Ir(1)–N(1) = 169.9(2), 171.6(2), C(23)–Ir(1)–N(3) = 167.7(2), 175.5(2), C(1)–Ir(1)–N(1) = 77.2(2), 76.7(2), C(11)–Ir(1)–N(3) = 80.1(2), 79.5(2), C(23)–Ir(1)–N(4) = 79.5(2), 77.6(3). (b) X-ray molecular structure of complex **6** (50% probability ellipsoids; hydrogen atoms have been omitted). Selected bond lengths (Å) and angles (deg): Ir–N(1) = 2.1420(18), Ir–N(3) = 2.153(2), Ir–N(4) = 2.1347(18), Ir–C(1) = 1.997(2), Ir–C(11) = 2.011(2), Ir–C(23) = 2.032(2), C(1)–N(2) = 1.330(3), N(2)–C(7) = 1.360(3), C(7)–N(1) = 1.339(3), C(1)–Ir–N(3) = 172.06(8); C(11)–Ir–N(1) = 170.38(8), C(23)–Ir–N(4) = 172.23(8), C(1)–Ir–N(1) = 76.90(8), C(23)–Ir–N(3) = 78.95(9), C(11)–Ir–N(4) = 80.62(8).

The NMR spectra of complexes **4**–**7**, in dichloromethane-*d*_2_ at 298 K, agree with the structures shown in Figure 2 and resemble those of **2** and **3**. In the ^1^H NMR spectra, the most salient signal is an AB spin system around 2.5 ppm, which correlates with a singlet in the ^13^C­{^1^H} NMR spectra around 60–62 ppm, due to the CH_2_ unit of the neopentyl substituent of the iridaimidazole rings. The ^13^C­{^1^H} NMR spectra show a characteristic low field signal between 240 and 250 ppm assigned to the metalated carbon atom of the new chelating ligands.

We monitored the reactions of dimer **1** with the amine-substituted heterocycles in toluene-*d*_8_ by ^1^H NMR spectroscopy, but the only organometallic species observed during the reactions are the starting dimer and the final products. Thus, in order to obtain some information about the reaction mechanism we have performed a computational study by DFT (B3LYP-D3//SDD­(f)/6–31G**) on the formation of complexes **2**–**7**, using 1-methyl-1*H*-imidazol-2-amine as model of the amine reagent (See Figure S13). Similar to the formation of the iridaoxazol derivative shown in Scheme 1,^15^ the formation of complexes **2**–**7** likely proceeds through the sequence of reactions depicted in Scheme 3. The rupture of the Ir-π-alkynyl bridges by coordination of the endocyclic N^3^ nitrogen atom of the corresponding azole to the metal center gives rise to intermediates **A**. The deprotonation of the 2-amino substituent of the heterocycles by the nucleophilic C_β_ atom of the σ-alkynyl ligand would give imine­(azolate)-iridium-vinylidene intermediates **B**. Then, the nucleophilic attack of the imine group to the C_α_ atom of the vinylidenes should afford intermediates **C**, which could evolve to the final iridaimidazoles via a 1,3-hydrogen shift from the NH-group of the iridacycle to the terminal carbon atom of the exocyclic double bond. This migration could be mediated by a second amine molecule. In fact, the energy barrier (Δ*G* ^⧧^ at 298 K) for the direct 1,3-hydrogen displacement calculated for the 1-methyl-1*H*-imidazol-2-amine derivative, 43.3 kcal mol^–1^, is much higher than the barrier for the assisted 1,3-hydrogen migration, 19.7 kcal mol^–1^ (Figure S13). Thus, the interaction of the NH group of **C** with the N^3^ atom of a second molecule of the amine-azol reagent, via a hydrogen bond, would give intermediates **D**. Next, in a concerted manner, the amine-azol molecule of the adducts **D** accepts the hydrogen atom of the NH group of the iridacycle while donating one of the amino hydrogens from the NH_2_ group to the C_β_ atom of the alkenyl unit generating the final products and releasing the imine tautomer of the amine reagent. This hydrogen shift takes place through eight-membered cyclic transition states, exemplified in Scheme 3 for complex **2** (**TS**_**DE**_).

**Scheme 3:**
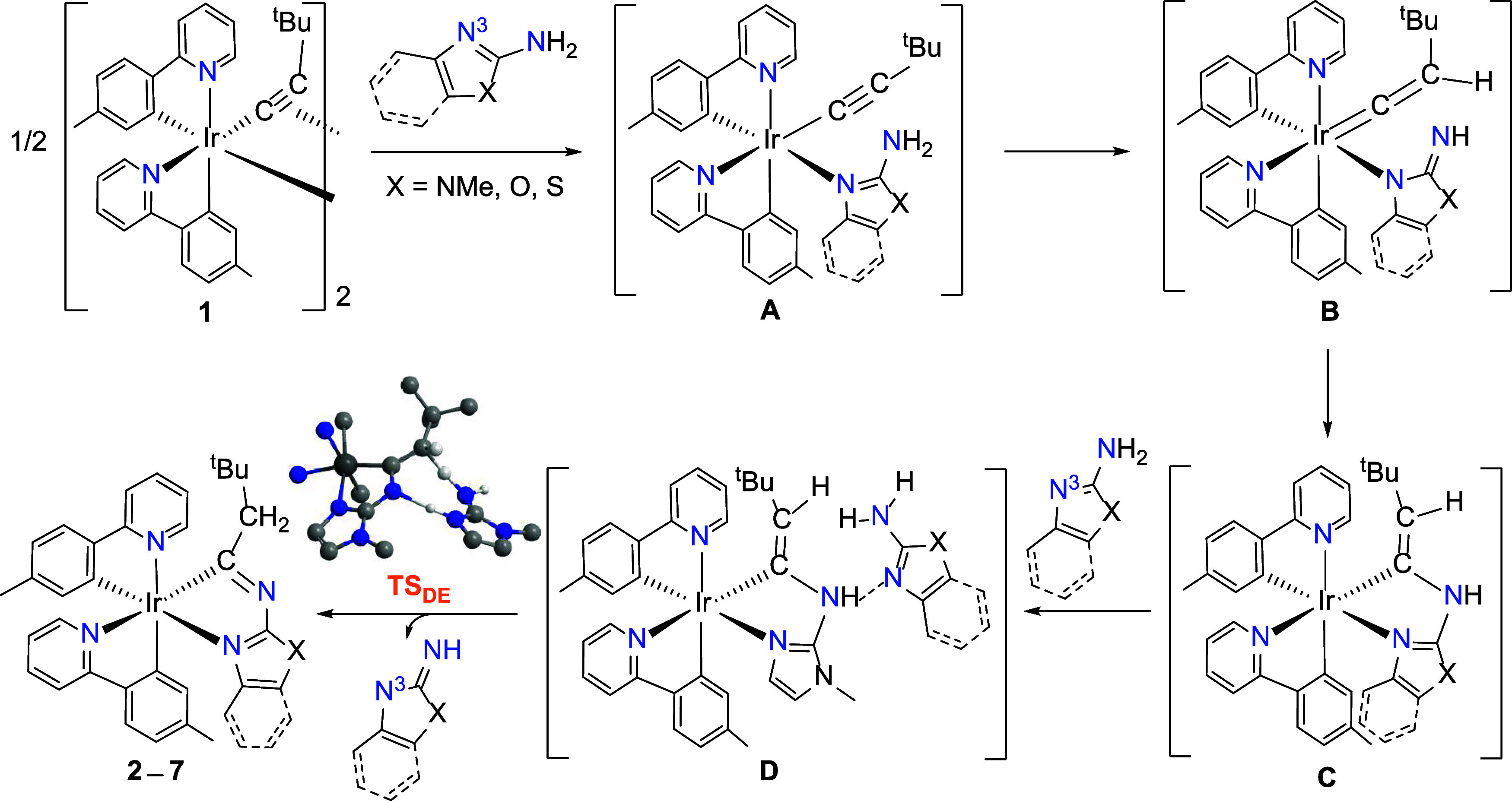
Possible Pathway for the Formation of the Iridaimidazole Complexes **2–7**

### Photophysical and Electrochemical Properties of the Iridaimidazole Derivatives

The ultraviolet–visible (UV–vis) spectra of 10^–4^ M solutions of complexes **2**–**7** were recorded in 2-methyltetrahydrofuran (2-MeTHF) at room temperature (Figures S15–S20) and the most notable absorptions are summarized in Table 1. To assign the observed bands to their respective electronic transitions, time-dependent density functional theory (TD-DFT) calculations were performed (B3LYP-D3//SDD­(f)/6–31G**), considering the solvent environment of tetrahydrofuran. The calculated electronic transitions, oscillator strengths, and the nature of the transitions are presented also in Tables 1 and S2–S7. Molecular orbitals plots are represented in Figures S21–S26. The spectra can be divided into three energy regions: below 350 nm, 350–450 nm, and above 450 nm. The most intense absorptions at wavelengths lower than 350 nm are attributed to intra- and interligand (LC and LLCT) ^1^π–π* transitions. The bands observed in the range of 350–450 nm are assigned to spin-allowed metal-to-ligand charge transfer (MLCT) combined with LC and LLCT processes. The weak absorption tails around 470–480 nm are ascribed to formally spin-forbidden transitions, predominantly HOMO–LUMO and HOMO–LUMO + 1 (complexes **2**–**5** and **7**), and HOMO–LUMO + 2 (complex **6**), produced by significant spin–orbit coupling induced by the iridium center. Analysis of the molecular orbitals (Tables S8–S13) indicates that the HOMO is mainly localized on d orbitals of the metal center (44–47%) and π orbitals of the 3b (35–45%) and 3b′ (10–18%) ligands. Conversely, the LUMO and LUMO + 1 are predominantly situated on the π* orbitals of the 3b ligands (90–97%) except for **7**, where they spread over 3b (49–53%) and 3b′ (44–45%) ligands. It is worth mentioning that the LUMO + 2 of complexes **2**, **4**, and **7** are almost exclusively localized on the 3b ligands (87–96%), while for **5** and **6** are mainly located on the 3b′ ligand (77–82%), and for complex **3** it is delocalized between the 3b (43%) and 3b′ (53%) groups.

**Table 1:** Selected Experimental UV–vis Absorptions in 2-MeTHF and Calculated (TD-DFT) Optical Transitions in THF for Complexes **2–7**

λ exp (nm)	ε (M^–1^ cm^–1^)	exitation energy (nm)	oscilator strength, *f*	transition	nature of the transition
Complex **2**					
308	13,588	306	0.0528	HOMO – 3 → LUMO (83%)	3b + 3b′ → 3b
344	7503	351	0.0359	HOMO → LUMO + 2 (64%)	Ir + 3b + 3b′ → 3b
387	5992	389	0.0773	HOMO – 1 → LUMO (93%)	Ir + 3b → 3b
433	2536	411 (S_1_)	0.009	HOMO → LUMO (96%)	Ir + 3b + 3b′→ 3b
487	410	457 (T_1_)	0	HOMO → LUMO (32%)	Ir + 3b + 3b′→ 3b
				HOMO → LUMO + 1 (31%)	
Complex **3**					
306	17,923	300	0.0717	HOMO – 3 → LUMO + 1 (67%)	3b + 3b′→ 3b
342	12,083	344	0.0556	HOMO – 2 → LUMO (68%)	Ir + 3b′→ 3b
388	6677	386	0.076	HOMO – 1 → LUMO (94%)	Ir + 3b → 3b
424	3569	407 (S_1_)	0.0083	HOMO → LUMO (96%)	Ir + 3b + 3b′→ 3b
478	455	454 (T_1_)	0	HOMO → LUMO + 1 (44%)	Ir + 3b + 3b′→ 3b
				HOMO → LUMO (23%)	
Complex **4**					
305	18,137	293	0.1727	HOMO – 3 → LUMO + 1 (73%)	3b + 3b′→ 3b
343	10,685	341	0.0656	HOMO – 2 → LUMO + 1 (58%)	Ir + 3b + 3b′ → 3b
380	8284	383	0.0825	HOMO – 1 → LUMO (92%)	Ir + 3b → 3b
416	4255	401 (S_1_)	0.0125	HOMO → LUMO (94%)	Ir + 3b + 3b′→ 3b
476	420	451 (T_1_)	0	HOMO → LUMO (29%)	Ir + 3b + 3b′→ 3b
				HOMO → LUMO + 1 (28%)	
Complex **5**					
309	16,692	292	0.1778	HOMO – 4 → LUMO + 1 (65%)	3b + 3b′→ 3b
343	12,474	332	0.0959	HOMO – 2 → LUMO (62%)	Ir + 3b + 3b′ → 3b
388	7078	379	0.0626	HOMO – 1 → LUMO (84%)	Ir + 3b → 3b
424	3454	396 (S_1_)	0.0176	HOMO → LUMO (92%)	Ir + 3b → 3b
475	479	449 (T_1_)	0	HOMO → LUMO (32%)	Ir + 3b → 3b
				HOMO → LUMO + 1 (22%)	
Complex **6**					
306	17,696	294	0.1611	HOMO – 3 → LUMO + 1 (68%)	3b + 3b′→ 3b
350	12,405	347	0.1057	HOMO – 2 → LUMO (89%)	Ir + 3b + 3b′ → 3b
405	5559	400 (S_1_)	0.0157	HOMO → LUMO (87%)	Ir + 3b → 3b
473	571	462 (T_1_)	0	HOMO → LUMO + 2 (47%)	Ir + 3b → 3b’
				HOMO – 2 → LUMO + 2 (24%)	
Complex **7**					
307	12,396	294	0.1843	HOMO – 4 → LUMO + 2 (40%)	3b + 3b′→ 3b
				HOMO – 3 → LUMO + 2 (22%)	
356	10,096	360	0.0395	HOMO – 1 → LUMO + 2 (91%)	Ir + 3b → 3b
400	4832	409 (S_1_)	0.0113	HOMO → LUMO (58%)	Ir + 3b → 3b+ 3b′
				HOMO → LUMO + 1 (35%)	
475	731	459 (T_1_)	0	HOMO → LUMO (35%)	Ir + 3b → 3b+ 3b′
				HOMO → LUMO + 1 (34%)	

The electrochemical properties of complexes **2**–**7** were investigated by cyclic voltammetry. The measurements were performed in dichloromethane under argon atmosphere, with tetrabutylammonium hexafluorophosphate as the supporting electrolyte at a concentration of 0.1 M. The voltammograms are presented in Figure S27. Table 2 summarizes the oxidation potentials referenced to the ferrocenium/ferrocene (Fc^+^/Fc) couple, along with the corresponding HOMO energy levels derived from these potentials. Additionally, the HOMO and LUMO energy levels calculated using density functional theory (DFT) are also included. All complexes display a reversible oxidation at the metal center within the potential range of 0.24–0.44 V. For all six complexes, the HOMO energies obtained from DFT calculations show a very good agreement with those estimated from the oxidation potentials. It should be noted that for the imidazole **2** and benzimidazole **3** derivatives, the oxidation potential is slightly lower (0.11–0.20 V) than for the oxygen (**4** and **5**) and sulfur (**6** and **7**) containing complexes, suggesting a modest destabilization of the HOMO of **2** and **3** compared to **4**–**7**, which is consistent with the calculated HOMO energy values. The HOMO–LUMO gap is similar in all of them, approximately 3.80 V.

**Table 2:** Electrochemical Data and DFT Molecular Orbital Energy Data for **2–7**

		obs (eV)	calcd (eV)		
complex	*E*_1/2_^ox^ (V)^a^	HOMO^b^	HOMO^c^	LUMO^c^	HLG^c^ ^,^ ^d^
**2**	0.24	–5.04	–4.95	–1.19	3.76
**3**	0.28	–5.08	–5.00	–1.21	3.79
**4**	0.42	–5.22	–5.11	–1.28	3.83
**5**	0.44	–5.24	–5.16	–1.28	3.88
**6**	0.40	–5.20	–5.14	–1.30	3.84
**7**	0.39	–5.19	–5.17	–1.31	3.86

a Measured in CH_2_Cl_2_ (10^–3^ M)/[Bu_4_N]­PF_6_ (0.1 M) solutions, under argon, vs Fc^+^/Fc at 0.1 V/s, at room temperature.

b HOMO = −[*E*_1/2_^ox^ vs Fc^+^/Fc + 4.8] eV.

c Values from electronic structure DFT calculations.

d HLG = LUMO – HOMO.

The new iridaimidazole complexes featuring different fused heterocycles are efficient phosphorescent emitters upon photoexcitation in poly­(methyl methacrylate) (PMMA) films, doped with 5% weight, at 298 K and in 2-MeTHF at 298 and 77 K. All of them emit in the green region of the spectrum with wavelength maxima between 474 and 558 nm (Table 3 and Figure 3). The fused heterocycle slightly influences the color of the emission. Thus, in general, complexes with a fused benzoxazole (**5**), thiazole (**6**), and benzothiazole (**7**) emit at lower energies than those with a fused imidazole (**2**), benzimidazole (**3**), and oxazole (**4**). The spectra of **2**, **3**, **4**, and **6** at 298 K display a structured band whereas those of **5** and **7** contain a broad and structureless band, that resolves into vibronic fine structures in 2-MeTHF at 77 K for **3** and **4**, which is indicative of emission from a mixture of ^3^MLCT, ^3^LC, and ^3^LLCT states. The estimated emission maxima, from the difference in energy between the optimized triplet states T_1_ and the singlet ground states S_0_ in tetrahydrofuran, closely match the experimental results, as expected for emissions corresponding to T_1_ excited states. The spin density distribution calculated for the optimized T_1_ of complexes **2**–**5** is mainly centered on the d orbitals of the metal and one of the two *p*-tolylpyridine ligands. However, for complexes **6** and **7**, which contain a sulfur atom, this spin density is localized, in addition to the metal center, in the thiazol and benzothiazol fragments (Figure 4). This suggest that, in accordance with the nature of the transitions of lower energy (S_0_ → T_1_) collected in Table 1, the emissions from the T_1_ excited states are originated by HOMO → LUMO and HOMO → LUMO + 1 charge transfer for complexes **2**–**5** and **7** but it involves also the LUMO + 2 orbital for complex **6**. The participation of the 3b′ ligands in the orbitals involved in the charge transfer processes that generate the triplets for **2**–**5** is residual, while for **6** and **7** its participation is very significant (Tables S2–S13), according to the spin density diagrams. This could explain why although the HOMO–LUMO separation is equal in all complexes, the emission color is slightly different from one to another. All complexes exhibit phosphorescence lifetimes ranging from 0.82 to 10.70 μs and good to excellent quantum yields (0.55 to 0.92). It is worth highlighting the quantum yields of the benzimidazole and benzothiazole derivatives **3** (0.91 in PMMA and 0.92 in 2-MeTHF) and **7** (0.80 in PMMA and 0.88 in 2-MeTHF), which are higher than those of their analogues without benzo group, **2** (0.68 in PMMA and 0.70 in 2-MeTHF) and **6** (0.63 in PMMA and 0.73 in 2-MeTHF).

**Figure 3:**
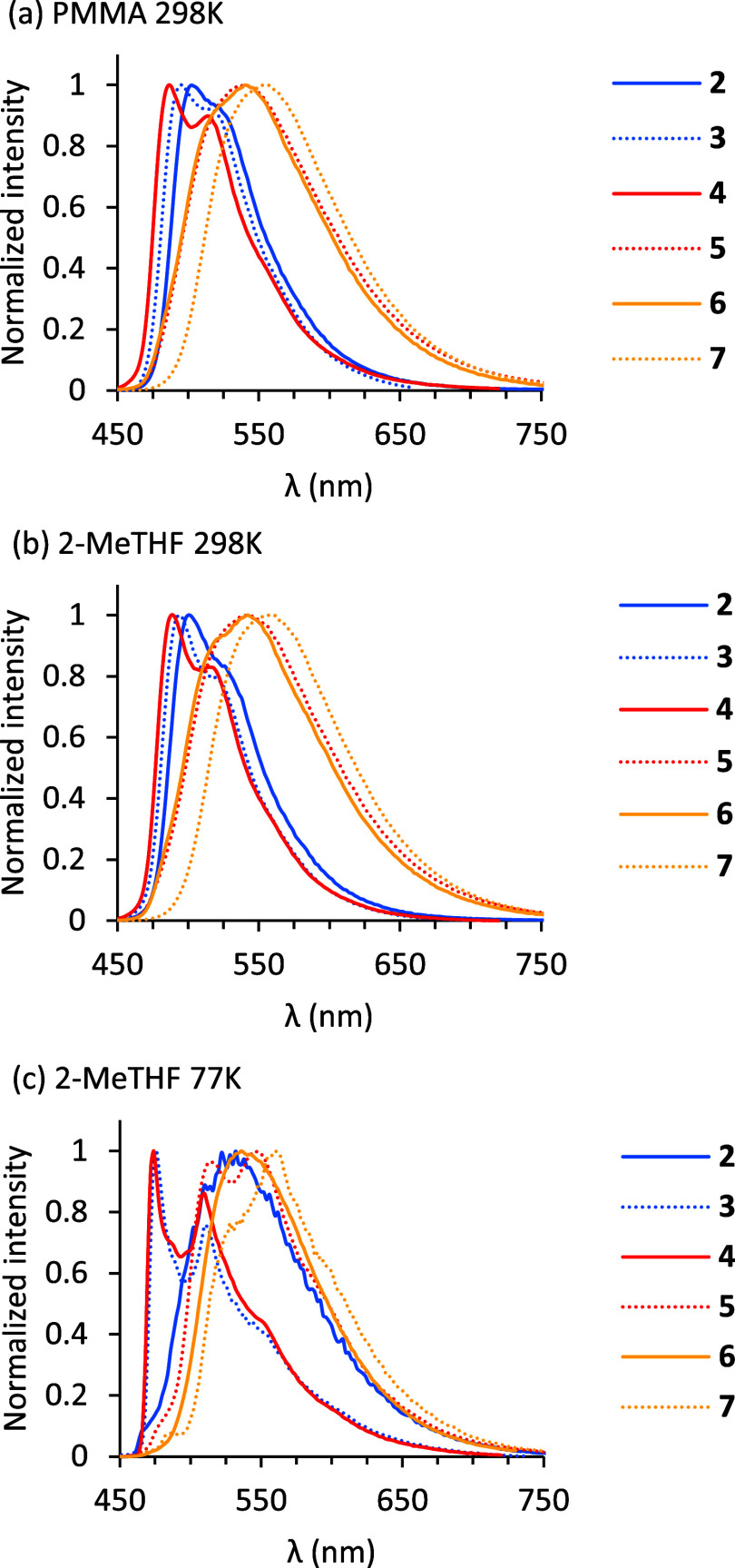
Emission spectra of **2**–**7** in (a) 5 wt % PMMA films at 298 K, (b) 2-MeTHF at 298 K, and (c) 2-MeTHF at 77 K.

**Figure 4:**
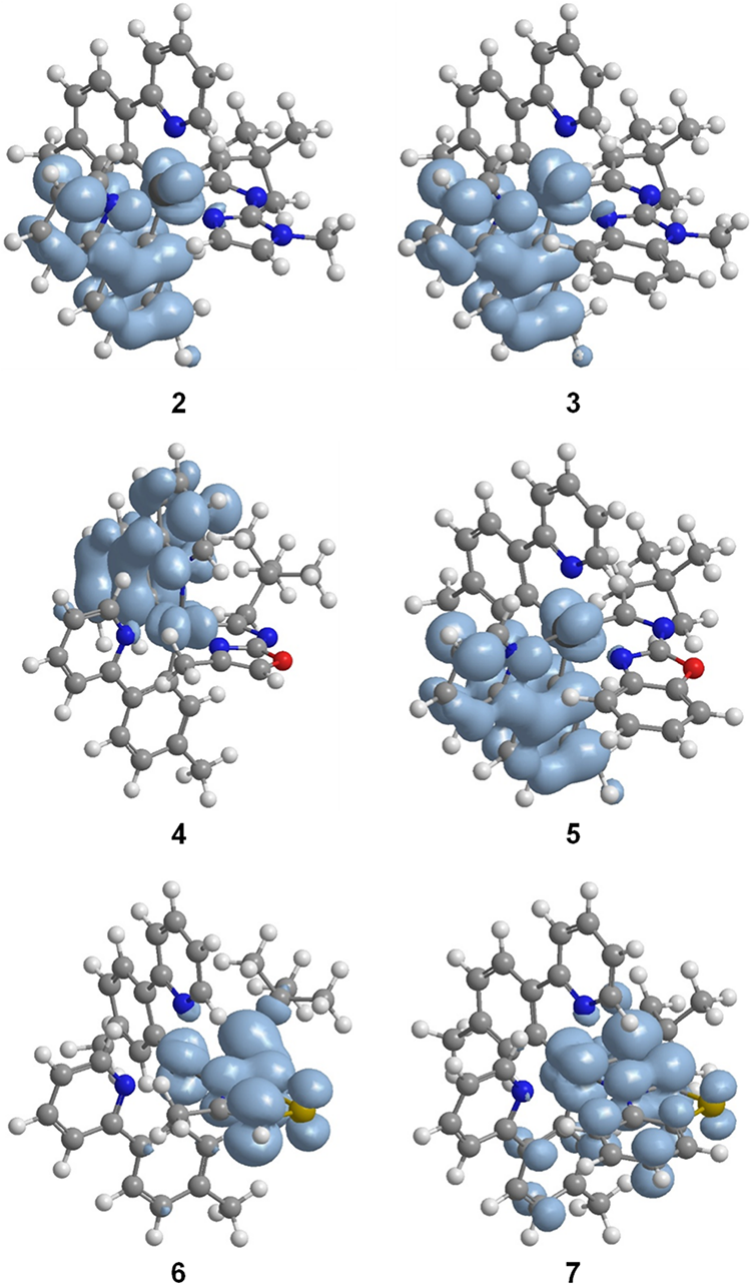
Spin density distributions for the optimized T_1_ state of complexes **2**–**7** (0.003 isovalue).

**Table 3:** Photophysical Data for Complexes **2–7**

calcd λ_em_ (nm)^a^	media (*T*/K)		λ_em_ (nm)	FWHM (nm)^b^	τ (μs)^c^	Φ	*k*_r_ (s^–1^)^d^	*k*_nr_ (s^–1^)^d^	*k*_r_/*k*_nr_
Complex **2**									
503	PMMA (298)	502_max_, 524_sh_		68	1.38 (1.45, 80.3%; 0.59, 19.7%)	0.68	4.9 × 10^5^	2.3 × 10^5^	2.13
	MeTHF (298)	500_max_, 528_sh_		67	1.77	0.70	3.9 × 10^5^	1.7 × 10^5^	0.04
	MeTHF (77)	532		98	7.73 (9.04, 49.5%; 5.70, 50.5%)				
Complex **3**									
498	PMMA (298)	496_max_, 520		68	1.50 (1.57, 85.1%; 0.67, 14.9%)	0.92	6.1 × 10^5^	5.3 × 10^4^	11.5
	MeTHF (298)	494_max_, 518_sh_		60	1.86	0.91	4.9 × 10^5^	4.8 × 10^4^	10.2
	MeTHF (77)	476_max_, 510, 554_sh_		61	3.68 (7.50, 3.8%; 3.36, 96.2%)				
Complex **4**									
493	PMMA (298)	486_max_, 514_sh_		68	1.24 (1.42, 60.0%; 0.55, 40.0%)	0.76	6.1 × 10^5^	1.9 × 10^5^	3.21
	MeTHF (298)	488_max_, 514		64	0.82	0.60	7.3 × 10^5^	4.9 × 10^5^	1.49
	MeTHF (77)	474_max_, 510, 554		69	3.79				
Complex **5**									
492	PMMA (298)	536		110	1.11 (1.24, 66.0%; 0.62, 34.0%)	0.67	1.2 × 10^5^	6.4 × 10^4^	1.88
	MeTHF (298)	542		110	1.23	0.55	6.8 × 10^4^	3.6 × 10^4^	1.89
	MeTHF (77)	515, 547_max_		100	3.90				
Complex **6**									
541	PMMA (298)	520_sh_, 542_max_		107	2.20 (2.29, 85%; 0.94, 15%)	0.63	2.9 × 10^5^	1.7 × 10^5^	1.71
	MeTHF (298)	518_sh_, 542_max_		106	2.62	0.73	2.8 × 10^5^	1.0 × 10^5^	2.80
	MeTHF (77)	536		90	10.70 (11.31, 80.4%; 5.71, 19.6%)				
**Complex 7**									
534	PMMA (298)	554		103	1.50 (1.58, 51.6%; 0.76, 18.4%)	0.80	5.3 × 10^5^	1.3 × 10^5^	4.08
	MeTHF (298)	558		104	1.96	0.88	4.5 × 10^5^	6.1 × 10^4^	7.38
	MeTHF (77)	532, 558_max_		94	6.71 (9.43, 28.9%; 4.28, 71.1%)				

a Estimated from TD-DFT calculations in THF at 298 K.

b Full width at half-maximum.

c Relative amplitudes (%) are given in parentheses for biexponential decays.

d Calculated according to *k*_r_ *=* Φ/τ and *k*_nr_ *=* (1 – Φ)/τ. For biexponential decays, the amplitude-weighted average lifetimes were used.

The comparison of the emissive properties of derivatives **2**-**7** with those of the related iridium­(III) complexes depicted in Scheme 1 reveals that although they are green emitters as the iridaimidazopyridine derivative (λ_em_ 473–513 nm) their quantum yields are lower.^14^ Unlike these green emitters, the iridaoxazoles derived from the reaction of the alkynyl bridging dimer **1** with amides are very weak orange emiters (λ_em_ 578–639 nm) with very low quantum yields (<0.10) and, in addition, they have very low chemical stability.^15^

## Conclusions

This study reinforces the role of alkynyl ligands as building blocks in organometallic chemistry and their usefulness for the synthesis of phosphorescent derivatives, that remains almost unexplored. Their postfunctionalization reactions can lead to the formation of novel ligand structures in the metal coordination sphere, unavailable by conventional coordination chemistry. So, the reactions between the alkynyl bridging dimer *cis*-[Ir­(μ-C≡C^t^Bu)­{κ^2^-*C*,*N*-(MeC_6_H_3_-py)}_2_]_2_ and amine-substituted five-membered heteroaromatic rings bearing two heteroatoms give rise to a new family of iridium­(III) green emitters containing two cyclometalated 2-*p*-tolylpyridine, with the heteroatoms mutually *cis* disposed, and a third unprecedented *C*,*N*-chelating ligand that give rise to iridaimidazole structures with a fused heterocyclic ring including *N*-methylimidazole, *N*-methylbenzimidazole, oxazole, benzoxazole, thiazole, and benzothiazole, which broadens the structural diversity of phosphorescent Ir­(III) complexes. The second heteroatom (N, O, or S) of the five membered heterocycle fused to the iridaimidazole moiety as well as the presence of the additional fused benzo unit slightly affect the photophysical properties of the emitters. Thus, complexes with benzoxazole, thiazole, and benzothiazole emit at slightly lower energies than the others, whereas derivatives with *N*-methylbenzimidazole, and benzothiazole exhibit high phosphorescence quantum yields (0.80–0.92) both in PMMA films and 2-MeTHF solutions at room temperature.

## Experimental Section

### General Information

All reactions were carried out under argon with dried solvents and using Schlenk tube techniques. Instrumental methods are given in the Supporting Information. In the NMR spectra, chemical shifts (expressed in ppm) are referenced to residual solvent peaks and coupling constants (*J*) are given in hertz. Signals were assigned using also bidimensional NMR spectra (^1^H–^1^H COSY, ^1^H–^13^C­{^1^H} HSQC and ^1^H–^13^C­{^1^H} HMBC). Complex *cis*-[Ir­(μ-C≡C^t^Bu)­{κ^2^-*C*,*N*-(MeC_6_H_3_-py)}_2_]_2_ (**1**) was prepared according to the published procedure.^14^

### Preparation of Ir­{κ^2^-*C*,*N*-(MeC_6_H_3_-py)}_2_{κ^2^-*C*,*N*-[C­(CH_2_^*t*^Bu)­N-im]} (**2**)

A mixture of **1** (100 mg, 0.082 mmol), 1-methyl-1*H*-imidazol-2-amine hydrochloride (22 mg, 0.164 mmol), and triethylamine (23 μL, 0.164 mmol) in toluene (6 mL), was heated in a Schlenk flask equipped with a PTFE stopcock for 24 h, at 120 °C. The resulting orange solution was concentrated until approximately 2 mL and pentane was added to give a yellow solid, which was washed with pentane (3 × 3 mL) and dried under vacuum. Yield: 40 mg (35%). Anal. Calcd for C_34_H_36_IrN_5_ (%): C, 57.77; H, 5.13; N, 9.91. Found: C, 57.37; H, 4.96; N, 9.44. HRMS (electrospray, *m*/*z*): Calcd for C_34_H_37_IrN_5_ [M + H]^+^: 708.2674; found: 708.2678. *T*_d_ = 302 °C.^17^ ^1^H NMR (300 MHz, CD_2_Cl_2_, 298 K): δ 7.88 (d, ^3^ *J*_H–H_ *=* 8.2, 1H, py), 7.76 (d, ^3^ *J*_H–H_ *=* 8.2, 1H, py), 7.75–7.66 (2H, py), 7.55–7.49 (3H, 1H py +2H MeC_6_ *H*_3_), 7.26 (d, ^3^ *J*_H–H_ *=* 5.5, 1H, py), 7.12 (s, 1H, MeC_6_ *H*_3_), 7.01 (ddd, ^3^ *J*_H–H_ *=* 7.1, ^3^ *J*_H–H_ *=* 5.6, ^4^ *J*_H–H_ *=* 1.3, 1H, py), 6.80–6.73 (2H, 1H py +1H MeC_6_ *H*_3_), 6.00–6.65 (3H, 2H MeC_6_ *H*_3_ + 1H im), 6.10 (d, ^3^ *J*_H–H_ *=* 1.7, 1H, im), 3.79 (s, 3H, NCH_3_), 2.47 (AB spin system, Δν = 35.0, *J*_A–B_ = 15.6, 2H, CH_2_), 2.32, 2.08 (both s, 3H each, *Me*C_6_H_3_), 0.69 (s, 9H, ^*t*^Bu). ^13^C­{^1^H} NMR (75 MHz, CD_2_Cl_2_, 298 K): δ 227.7 (C_q_, Ir–C=N), 167.6, 166.7 (both C_q_, py), 165.4 (C_q_, im), 161.9, 160.6 (both C_q_, MeC_6_H_3_), 148.5, 147.3 (both CH, py), 142.5, 141.7 (both C_q_, Ir–C MeC_6_H_3_), 140.3, 139.6 (both C_q_, MeC_6_H_3_), 138.8, 138.4 (both CH, MeC_6_H_3_), 136.8, 136.5 (both CH, py), 124.3, 124.2 (both CH, MeC_6_H_3_), 122.1 (CH, py), 121.6, 121.5 (both CH, MeC_6_H_3_-py), 121.3 (CH, py), 121.1 (CH, im), 119.0, 118.5 (both CH, py), 117.4 (CH, im), 59.8 (CH_2_), 33.8 (NCH_3_), 32.5 (C_q_, ^*t*^Bu), 30.4 (CH_3_, ^*t*^Bu), 21.9 (CH_3_, *Me*C_6_H_3_).

### Preparation of Ir­{κ^2^-*C*,*N*-(MeC_6_H_3_-py)}_2_{κ^2^-*C*,*N*-[C­(CH_2_^*t*^Bu)­N-bzim]} (**3**)

A mixture of **1** (300 mg, 0.246 mmol) and 1-methyl-1*H*-benzo­[*d*]­imidazol-2-amine (72 mg, 0.492 mmol) in toluene (18 mL), was heated in a Schlenk flask equipped with a PTFE stopcock for 24 h, at 120 °C. The resulting orange solution was concentrated until approximately 2 mL and pentane was added to give a yellow solid, which was washed with pentane (3 × 3 mL) and dried under vacuum. Yield: 150 mg (40%). Anal. Calcd for C_38_H_38_IrN_5_ (%): C, 60.30; H, 5.06; N, 9.25. Found: C, 59.98; H, 5.01; N, 9.39. HRMS (electrospray, *m*/*z*): Calcd for C_38_H_38_IrN_5_ [M]^+^: 758.2831; found: 758.2814. *T*_d_ = 335 °C.^17^ ^1^H NMR (300 MHz, CD_2_Cl_2_, 298 K): δ 7.91 (d, ^3^ *J*_H–H_ *=* 8.1, 1H, py), 7.82 (d, ^3^ *J*_H–H_ *=* 8.2, 1H, py), 7.72–7.61 (3H, py), 7.56 (d, ^3^ *J*_H–H_ *=* 8.0, 1H, MeC_6_ *H*_3_), 7.50 (d, ^3^ *J*_H–H_ *=* 7.9, 1H, MeC_6_ *H*_3_), 7.42 (ddd, ^3^ *J*_H–H_ *=* 5.6, ^4^ *J*_H–H_ *=* 1.6, ^4^ *J*_H–H_ *=* 0.8, 1H, py), 7.24 (d, ^3^ *J*_H–H_ *=* 7.9, 1H, bzim), 7.06 (ddd, ^3^ *J*_H–H_ *=* 8.3, ^3^ *J*_*H–H*_ *=* 7.5, ^4^ *J*_H–H_ *=* 1.2, 1H, bzim), 7.06 (s, 1H, MeC_6_ *H*_3_), 6.94–6.85 (m, 2H, py), 6.80 (ddd, ^3^ *J*_H–H_ *=* 8.1, ^3^ *J*_H–H_ *=* 7.7, ^4^ *J*_H–H_ *=* 1.2, 1H, bzim), 6.76–6.70 (3H, MeC_6_ *H*_3_), 5.91 (d, ^3^ *J*_H–H_ *=* 7.9, 1H, bzim), 3.94 (s, 3H, NCH_3_), 2.46 (AB spin system, Δν = 19.0, *J*_A–B_ = 15.8, 2H, CH_2_), 2.28, 2.11 (both s, 3H each, *Me*C_6_H_3_), 0.76 (s, 9H, ^*t*^Bu). ^13^C­{^1^H} NMR (75 MHz, CD_2_Cl_2_, 298 K): δ 239.7 C_q_, (Ir–C=N), 169.7 (C_q_, CN_3_ bzim), 167.5, 167.0 (both C_q_, py), 161.3, 159.7 (both C_q_, MeC_6_H_3_), 149.2, 147.7 (both CH, py), 142.6, 141.6 (both C_q_, Ir–C MeC_6_H_3_), 140.6, 139.9 (both C_q_, MeC_6_H_3_), 139.5 (C_q_, bzim), 138.7, 138.2 (both CH, MeC_6_H_3_), 137.1, 137.0 (both CH, py), 135.4 (C_q_, bzim), 124.3, 124.3 (both CH, MeC_6_ *H*_3_), 122.6 (CH, Bzim), 122.3 (CH, py), 122.0, 121.7 (both CH, MeC_6_H_3_), 121.4 (CH, py), 120.9 (CH, bzim), 119.1, 118.7 (both CH, py), 113.6, 110.1 (both CH, bzim), 61.3 (CH_2_), 32.7 (C_q_, ^*t*^Bu), 31.0 (NCH_3_, bzim), 30.5 (CH_3_, ^*t*^Bu), 22.0, 21.9 (both CH_3_, *Me*C_6_H_3_).

### Preparation of Ir­{κ^2^-*C*,*N*-(MeC_6_H_3_-py)}_2_{κ^2^-*C*,*N*-[C­(CH_2_^*t*^Bu)*N*-oxazol]} (**4**)

It was prepared following the same procedure as for **3**, starting from **1** (300 mg, 0.246 mmol) and 4-methyloxazol-2-amine (48 mg, 0.492 mmol). Yellow solid. Yield: 160 mg (46%). Anal. Calcd for C_34_H_35_IrN_4_O (%): C, 57.69; H, 4.98; N, 7.91. Found: C, 57.65; H, 5.21; N, 8.07. HRMS (electrospray, *m*/*z*): Calcd for C_34_H_36_IrN_4_O [M + H]^+^: 709.2514; found: 709.2535. *T*_d_ = 309 °C.^17^ ^1^H NMR (300 MHz, CD_2_Cl_2_, 298 K): δ 7.96–7.86 (2H, py), 7.81–7.71 (2H, py), 7.58–7.50 (3H, 1H py +2H MeC_6_ *H*_3_), 7.23 (ddd, ^3^ *J*_H–H_ *=* 5.5, ^4^ *J*_H–H_ *=* 1.4, ^4^ *J*_*H–H*_ *=* 0.8, 1H, py), 7.15 (q, ^4^ *J*_H–H_ *=* 1.3, 1H, oxazol), 7.08 (ddd, ^3^ *J*_H–H_ *=* 7.1, ^3^ *J*_H–H_ *=* 5.5, ^4^ *J*_H–H_ *=* 1.3, 1H, py), 7.03 (s, 1H, MeC_6_ *H*_3_), 6.84 (d, ^3^ *J*_H–H_ *=* 8.1, 1H, MeC_6_ *H*_3_), 6.78 (ddd, ^3^ *J*_H–H_ *=* 7.2, ^3^ *J*_H–H_ *=* 5.7, ^4^ *J*_H–H_ *=* 1.3, 1H, py), 6.71 (d, ^3^ *J*_H–H_ *=* 7.7, 1H, MeC_6_ *H*_3_), 6.62 (1H, MeC_6_ *H*_3_), 2.54 (s, 2H, CH_2_), 2.31, 2.08 (both s, 3H each, *Me*C_6_H_3_), 1.32 (d, ^4^ *J*_H–H_ = 1.3 Hz, 3H, *Me*-oxazol), 0.67 (s, 9H, ^*t*^Bu). ^13^C­{^1^H} NMR (75 MHz, CD_2_Cl_2_, 298 K): δ 240.4 (Ir–C=N), 178.4 (C_q_, CN_2_O oxazol), 167.4, 166.8 (both C_q_, py), 159.5, 158.7 (both C_q_, MeC_6_H_3_), 149.5, 147.5 (both CH, py), 142.3, 141.4 (both C_q_, Ir–C MeC_6_H_3_), 141.0, 140.0 (both C_q_, MeC_6_H_3_), 138.8, 138.0 (both CH, MeC_6_H_3_), 137.3, 137.1 (both CH, py), 135.0 (C_q_, Me-*C* oxazol), 131.1 (CHO oxazol), 124.4 (2 CH MeC_6_H_3_), 122.5 (CH, py), 122.3, 122.2 (both CH, MeC_6_H_3_), 121.6, 119.4, 118.7 (all CH, py), 61.2 (CH_2_), 32.7 (C_q_, ^*t*^Bu), 30.4 (CH_3_^*t*^Bu), 22.0 (2 CH_3_, *Me*C_6_H_3_), 9.7 (CH_3_, *Me*-oxazol).

### Preparation of Ir­{κ^2^-*C*,*N*-(MeC_6_H_3_-py)}_2_{κ^2^-*C*,*N*-[C­(CH_2_^*t*^Bu)*N*-bzoxazol]} (**5**)

It was prepared following the same procedure as for **3**, starting from **1** (200 mg, 0.164 mmol) and benzo­[*d*]­oxazol-2-amine (44 mg, 0.328 mmol). Yellow solid. Yield: 110 mg (45%). Anal. Calcd for C_37_H_35_IrN_4_O (%): C, 59.74; H, 4.74; N, 7.53. Found: C, 59.58; H, 4.85; N, 7.64. HRMS (electrospray, *m*/*z*): Calcd for C_37_H_36_IrN_4_O [M + H]^+^: 745.2515; found: 745.2544. *T*_d_ = 314 °C.^17^ ^1^H NMR (300 MHz, CD_2_Cl_2_, 298 K): δ 7.94 (d, ^3^ *J*_H–H_ *=* 8.3, 1H, py), 7.88–7.80 (2H, py), 7.74 (ddd, ^3^ *J*_H–H_ *=* 8.4, ^3^ *J*_H–H_ *=* 7.9, ^4^ *J*_H–H_ *=* 1.4, 1H, py), 7.67 (ddd, ^3^ *J*_H–H_ *=* 8.2, ^3^ *J*_H–H_ *=* 7.5, ^4^ *J*_H–H_ *=* 1.3, 1H, py), 7.57 (d, ^3^ *J*_H–H_ *=* 7.9, 1H, MeC_6_ *H*_3_), 7.52 (d, ^3^ *J*_H–H_ *=* 7.9, 1H, MeC_6_ *H*_3_), 7.44 (d, ^3^ *J*_H–H_ *=* 8.1, 1H, bzoxazol), 7.36 (d, ^3^ *J*_H–H_ *=* 5.6, 1H, py), 7.12 (ddd, ^3^ *J*_H–H_ *=* 8.1, ^3^ *J*_H–H_ *=* 7.7, ^4^ *J*_H–H_ *=* 1.3, 1H, CH bzoxazol), 7.03–6.86 (4H, 1H MeC_6_ *H*_3_ + 2H py +1H bzoxazol), 6.78 (d, ^3^ *J*_H–H_ *=* 7.9, 1H, MeC_6_ *H*_3_), 6.74 (d, ^3^ *J*_H–H_ *=* 8.0, 1H, MeC_6_ *H*_3_), 6.70 (s, 1H, MeC_6_ *H*_3_), 6.00 (d, ^3^ *J*_H–H_ *=* 7.8, 1H, CH bzoxazol), 2.66 (AB spin system, Δν = 42.1, *J*_A–B_ = 15.3, 2H, CH_2_), 2.28, 2.11 (both s, 3H each, *Me*C_6_H_3_), 0.71 (s, 9H, ^*t*^Bu). ^13^C­{^1^H} NMR (75 MHz, CD_2_Cl_2_, 298 K): δ 250.4 (Ir–C=N), 167.3, 166.7 (both C_q_, py), 158.9, 157.8 (both C_q_, MeC_6_H_3_), 150.8 (C_q_, CN_2_O bzoxazol), 149.7, 147.5 (both CH, py), 142.4 (Ir–C MeC_6_H_3_), 141.5 (C_q_, bzoxazol), 141.3 (Ir–C MeC_6_H_3_), 141.1, 140.2 (both C_q_, MeC_6_H_3_), 139.7 (C_q_, bzoxazol), 138.8, 138.1 (both CH, MeC_6_H_3_), 137.6, 137.5 (both CH, py), 125.2 (CH, bzoxazol), 124.5, 124.4 (both CH, MeC_6_H_3_), 123.0 (CH, bzoxazol), 122.6 (CH, py), 122.5, 122.4 (both CH, MeC_6_H_3_), 121.6, 119.5, 118.9 (all CH, py), 113.9, 111.5 (both CH, bzoxazol), 62.3 (CH_2_), 33.0 (C_q_, ^*t*^Bu), 30.5­(CH_3_, ^*t*^Bu), 22.0, 21.9 (both CH_3_, *Me*C_6_H_3_).

### Preparation of Ir­{κ^2^-*C*,*N*-(MeC_6_H_3_-py)}_2_{κ^2^-*C*,*N*-[C­(CH_2_^*t*^Bu)*N*-thiazol]} (**6**)

It was prepared following the same procedure as for **3**, starting from **1** (300 mg, 0.246 mmol) and 4-methylthiazol-2-amine (56 mg, 0.492 mmol). Yellow solid. Yield: 120 mg (34%). Anal. Calcd for C_34_H_35_IrN_4_S (%): C, 56.41; H, 4.87; N, 7.74. Found: C, 56.62; H, 5.15; N, 7.45. HRMS (electrospray, *m*/*z*): Calcd for C_34_H_36_IrN_4_S [M + H]^+^: 725.2284; found: 725.2313. *T*_d_ = 317 °C.^17^ ^1^H NMR (300 MHz, CD_2_Cl_2_, 298 K): δ 7.91 (d, ^3^ *J*_H–H_ *=* 8.2, 1H, py), 7.74 (3H, py), 7.54 (3H, 1H py +2H MeC_6_ *H*_3_), 7.21 (d, ^3^ *J*_H–H_ *=* 5.8, 1H, py), 7.04 (ddd, ^3^ *J*_H–H_ *=* 7.2, ^3^ *J*_H–H_ *=* 5.5, ^4^ *J*_H–H_ *=* 1.4, 1H, py), 6.99 (s, 1H, MeC_6_ *H*_3_), 6.85–6.74 (2H, 1H MeC_6_ *H*_3_ + 1H py), 6.71 (d, ^3^ *J*_H–H_ *=* 7.5, 1H, MeC_6_ *H*_3_), 6.54 (2H, 1H MeC_6_ *H*_3_ + 1H thiazol), 2.56 (AB spin system, Δν = 31.3, *J*_A–B_ = 15.1, 2H, CH_2_), 2.29, 2.06 (both s, 3H, *Me*C_6_H_3_), 1.47 (br s, 3H, *Me*-thiazol), 0.65 (s, 9H, ^*t*^Bu). ^13^C­{^1^H} NMR (75 MHz, CD_2_Cl_2_, 298 K): δ 238.5 (C_q_, Ir–C=N), 167.3 (C_q_, py), 166.5 (C_q_, CN_2_O thiazol), 164.9 (C_q_, py), 159.7, 158.6 (both C_q_, MeC_6_H_3_), 149.7 (CH py), 148.4 (C_q_, Me-*C* thiazol), 147.3 (CH py), 142.1, 141.2 (both C_q_, Ir–C MeC_6_H_3_), 140.8, 140.1 (both C_q_, MeC_6_H_3_), 138.7, 137.6 (both CH, MeC_6_H_3_), 137.3, 137.0 (both CH, py), 124.5, 124.3 (both CH, MeC_6_H_3_), 122.5 (CH, py), 122.4, 122.1 (both CH, MeC_6_H_3_), 121.6, 119.5, 118.8 (all CH, py), 109.0 (CH, thiazol), 60.3 (CH_2_), 32.9 (C_q_, ^*t*^Bu), 30.5 (CH_3_^*t*^Bu), 22.0, 21.9 (both CH_3_, *Me*C_6_H_3_), 16.1 (CH_3_, *Me*-thiazol).

### Preparation of Ir­{κ^2^-*C*,*N*-(MeC_6_H_3_-py)}_2_{κ^2^-*C*,*N*-[C­(CH_2_^*t*^Bu)*N*-bzthiazol]} (**7**)

It was prepared following the same procedure as for **3**, starting from **1** (100 mg, 0.082 mmol) and benzo­[*d*]­thiazol-2-amine (25 mg, 0.164 mmol). Yellow solid. Yield: 50 mg (40%). Anal. Calcd for C_37_H_35_IrN_4_S (%): C, 58.48; H, 4.64; N, 7.37. Found: C, 58.21; H, 4.39; N, 7.59. HRMS (electrospray, *m*/*z*): Calcd for C_37_H_36_IrN_4_S [M + H]^+^: 761.2285; found: 761.2262. *T*_d_ = 311 °C.^17^ ^1^H NMR (300 MHz, CD_2_Cl_2_, 298 K): δ 7.92 (d, ^3^ *J*_H–H_ *=* 8.2, 1H, py), 7.84 (d, ^3^ *J*_H–H_ *=* 8.3, 1H, py), 7.75–7.62 (4H, 3H py +1H bzthiazol), 7.56, 7.52 (both d, ^3^ *J*_H–H_ *=* 8.0, 1H each, MeC_6_ *H*_3_), 7.35 (d, ^3^ *J*_H–H_ *=* 6.1, 1H, py), 7.12 (ddd, ^3^ *J*_H–H_ *=* 8.3, ^3^ *J*_H–H_ *=* 7.5, ^4^ *J*_H–H_ *=* 1.0, 1H, bzthiazol), 6.98–6.87 (4H, 1H MeC_6_ *H*_3_ + 2H py +1H bzthiazol), 6.79–6.72 (2H, MeC_6_ *H*_3_), 6.60 (s, 1H, MeC_6_ *H*_3_), 6.15 (d, ^3^ *J*_H–H_ *=* 8.1, 1H, bzthiazol), 2.66 (AB spin system, Δν = 62.1, *J*_A–B_ = 15.0, 2H, CH_2_), 2.26, 2.09 (both s, 3H each, *Me*C_6_H_3_), 0.70 (s, 9H, ^*t*^Bu). ^13^C­{^1^H} NMR (75 MHz, CD_2_Cl_2_, 298 K): δ 247.0 (s, Ir–C=N), 167.2, 166.6 (both C_q_, py), 159.2, 158.0 (both C_q_, MeC_6_H_3_), 149.8­(CH py), 149.7 (C_q_, bzthiazol), 147.3 (CH, py), 142.2, 141.2 (both C_q_, Ir–C MeC_6_H_3_), 141.0 (C_q_, MeC_6_H_3_), 140.8 (C_q_, bzthiazol), 140.3 (C_q_, MeC_6_H_3_), 138.7, 137.7 (both CH, MeC_6_H_3_), 137.5, 137.4 (both CH, py), 134.5 (C_q_, bzthiazol), 127.0 (CH, bzthiazol), 124.5, 124.4 (both CH, MeC_6_H_3_), 123.4, 123.2 (both CH, bzthiazol), 122.7 (CH, py), 122.6, 122.3 (both CH, MeC_6_H_3_), 121.7, 119.5, 119.1 (all CH, py), 117.3 (CH, bzthiazol), 61.3 (CH_2_), 33.0 (C_q_, ^*t*^Bu), 30.5 (CH_3_, ^*t*^Bu), 22.0 (2 CH_3_, *Me*C_6_H_3_).

## Supplementary Material





## Notes

General information for the experimental section, NMR spectra, structural analysis, computational details and energies of optimized structures, experimental and computed UV/vis spectra, molecular orbitals, cyclic voltammograms, normalized excitation and emission spectra, and TGA curves (PDF)
Cartesian coordinates of the optimized structures (XYZ)
