# Supplementary material for: Addition of Amine-Substituted Heteroaromatic Rings to Alkynyl Bridging Ligands to Generate Phosphorescent Emitters Incorporating Iridaimidazoles with Fused Heterocyclic Rings
Source: Inorg Chem. 2025 Dec 24;65(1):582–92. doi: 10.1021/acs.inorgchem.5c04705 (PMC12801302; doi:10.1021/acs.inorgchem.5c04705)
Supplement: Supplementary file 1 [file ic5c04705_si_001.pdf]

Supporting Information For

Addition of Amine-Substituted Heteroaromatic Rings to Alkynyl Bridging Ligands to Generate Phosphorescent Emitters Incorporating Iridaimidazoles with Fused Heterocyclic Rings

*Cristina Martín-Escura, Enrique Oñate, Montserrat Oliván, and Ana M. López**

Departamento de Química Inorgánica, Instituto de Síntesis Química y Catálisis Homogénea (ISQCH), Centro de Innovación en Química Avanzada (ORFEO-CINQA), Universidad de Zaragoza – CSIC, 50009 Zaragoza, Spain

*Corresponding author's e-mail address: amlopez@unizar.es

CONTENTS

Experimental Section: General Information S3

NMR spectra S4

Structural Analysis of Complexes 3, 4, and 6 S10

Computational Details S11

Energies of Optimized Structures S12

NICS and NICSzz for complexes 2−7 S16

Induced current density (AICD) plots of complexes 2−7 S17

UV-Vis Spectra of Complexes 2−7 (Observed and Calculated) S18

Analysis of Computed UV/Vis Data of 2−7 S21

Theoretical Analysis of Molecular Orbitals of 2−7 S27

Cyclic Voltammograms S39

Normalized Excitation and Emission Spectra of Complexes 2−7 S40

Time-resolved Photoluminescence Decay of Complexes 2−7 S46

TGA curves of complexes 2−7 S55

References S56

Experimental Section: General Information. All reactions were performed with exclusion of air at an argon/vacuum manifold using standard Schlenk-tube or glovebox techniques. Solvents were obtained oxygen-and water-free from an MBraun solvent purification apparatus or were dried by the usual procedures and distilled under argon atmosphere. NMR spectra were recorded on a Bruker Avance 300 MHz instruments. Chemical shifts (expressed in parts per million) are referenced to residual solvent peaks (^{1}H, ^{13}C{^{1}H}). Coupling constants *J* are given in Hertz. Elemental analyses were carried out in a Perkin-Elmer 2400 CHNS/O Series II analyzer. High-resolution electrospray (HRMS) mass spectra were acquired using a MicroTOF-Q hybrid quadrupole time-of-flight spectrometer (Bruker Daltonics, Bremen, Germany). UV-visible spectra were registered on a JASCO V-780 spectrophotomer. Steady-state photoluminescence spectra and lifetimes were recorded on a PicoQuant FluoTime 300 spectrometer. Data were fitted to either monoexponential or biexponential functions. Quantum yields were measured using the Hamamatsu Absolute PL Quantum Yield Measurement System C11347-11 (an uncertainty of ±5% is estimated). PMMA films at 5% weight were prepared in a glove box: 19 mg of PMMA (average M_w 97 000, average M_n 46 000) was dissolved in 1.0 mL of dichloromethane and then 1 mg of the iridium complex (5 wt %) was added with stirring to form a solution, which was filtered through a PTFE syringe filter (0.22 micron pore size), drop-coated onto a quartz substrate, and dried at room temperature. Cyclic voltammetry measurements were performed using a Bipotentiostat/Galvanostat μSTAT400 (Dropsens) controlled with DropView 8400 software. The electrochemical cell (1 mL volume) was a DRP-PTGRID-TRANSCELL (DropSens). It contains Pt grid working electrode (0.6 × 0.4 cm) which allows the bulk electrolysis of the solution contained in the cell, a Ag/AgCl reference electrode, and a platinum counter electrode. The experiments were carried out under argon in dichloromethane solutions (10^{-3} M), with Bu_4NPF_6 as supporting electrolyte (0.1 M). Scan rate was 100 mVs^{-1}. The potentials were referenced to the ferrocenium/ferrocene (Fc^+/Fc) couple. Thermogravimetric analyses were performed in a thermobalance from TA Instruments (model SDT 2960) by heating the solids under nitrogen (100 mL/min) at 10 °C/min.

NMR spectra

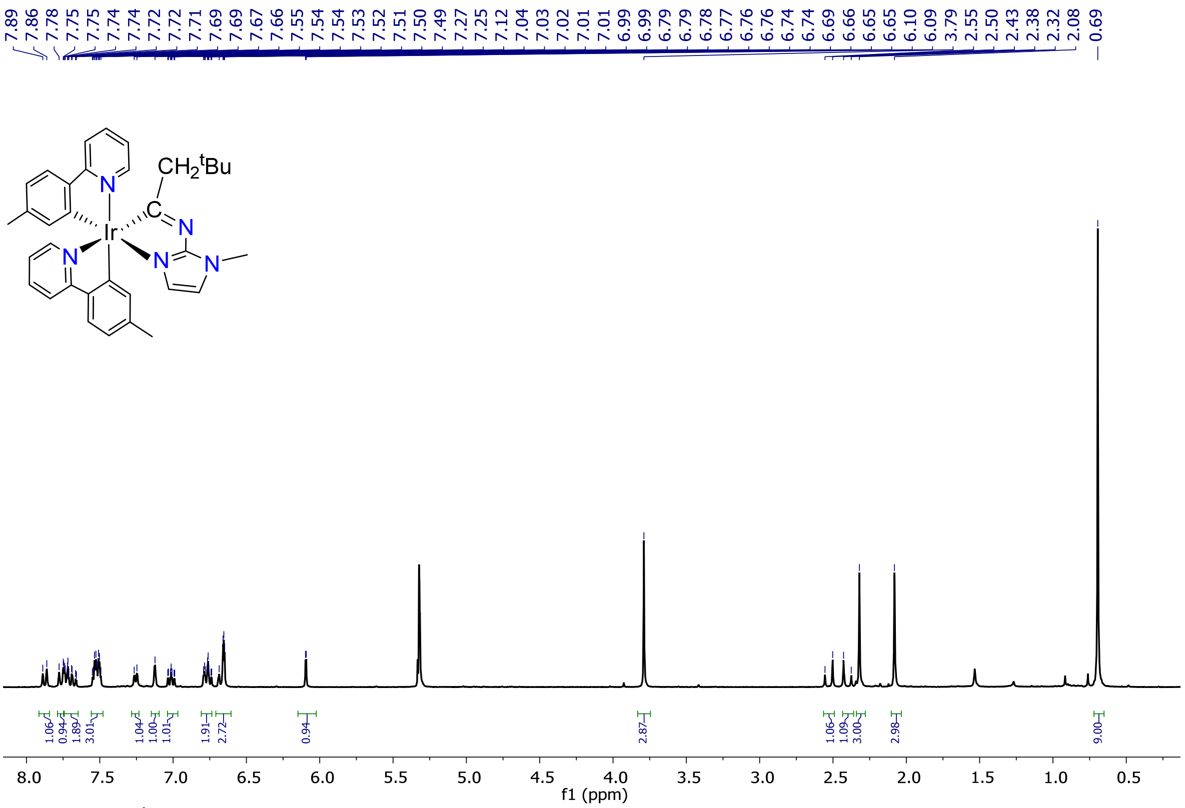

Figure S1. ^{1}H-NMR (300 MHz, CD_2Cl_2, 298 K) of **2**.

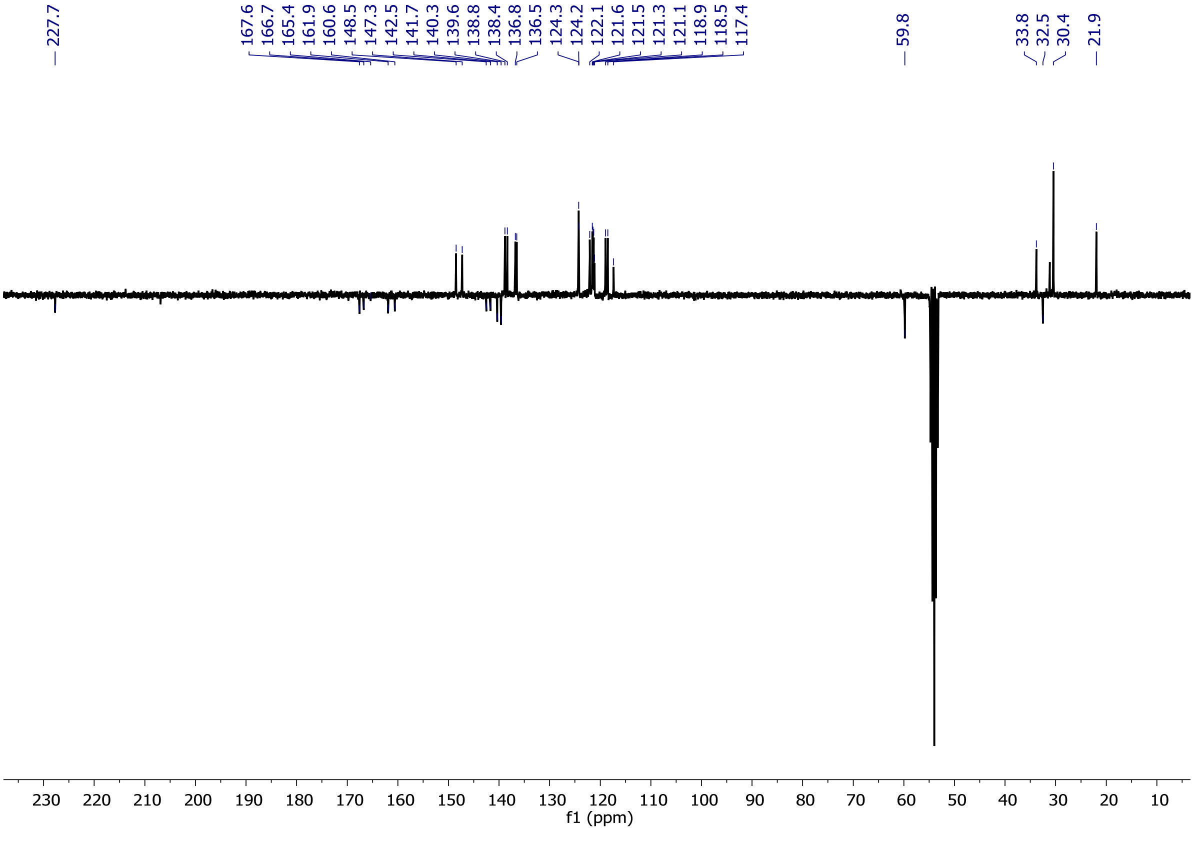

Figure S2. ^{13}C{^{1}H}-apt NMR (75 MHz, CD_2Cl_2, 298 K) of **2**.

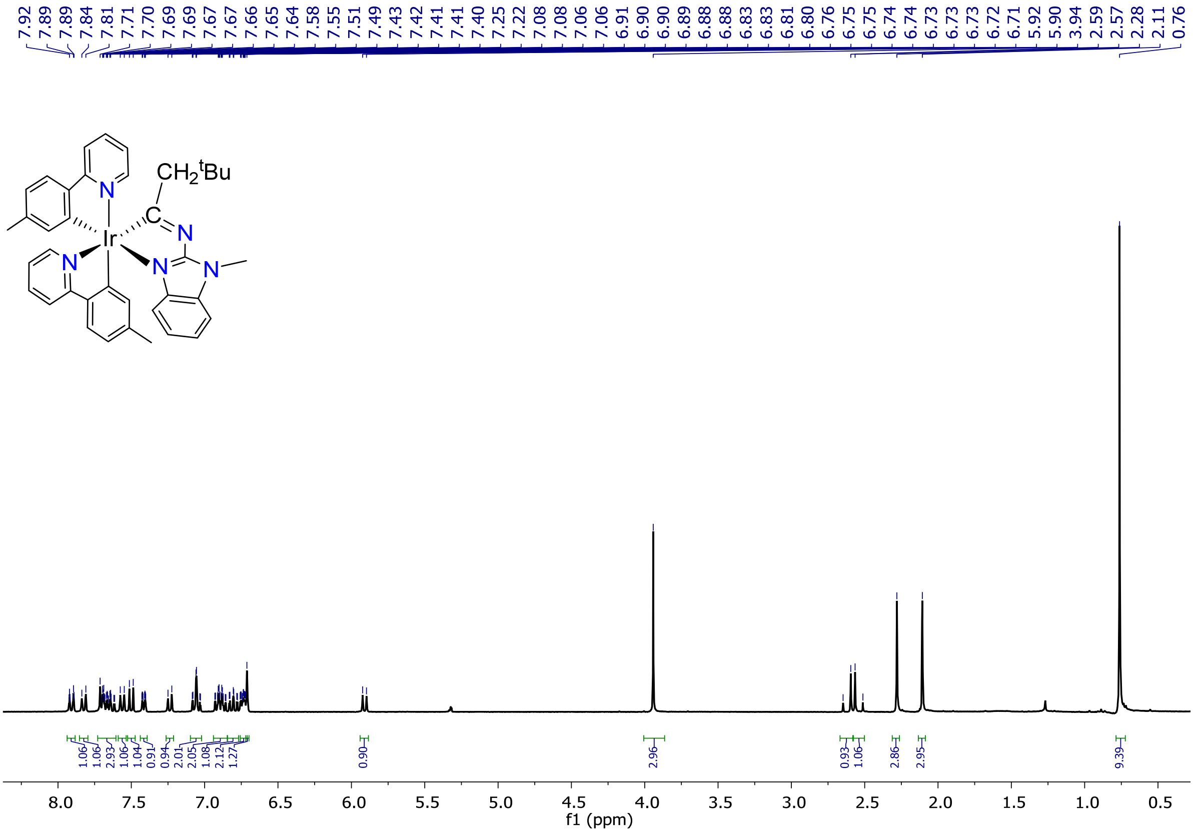

Figure S3. ^{1}H-NMR (300 MHz, CD_2Cl_2, 298 K) of **3**.

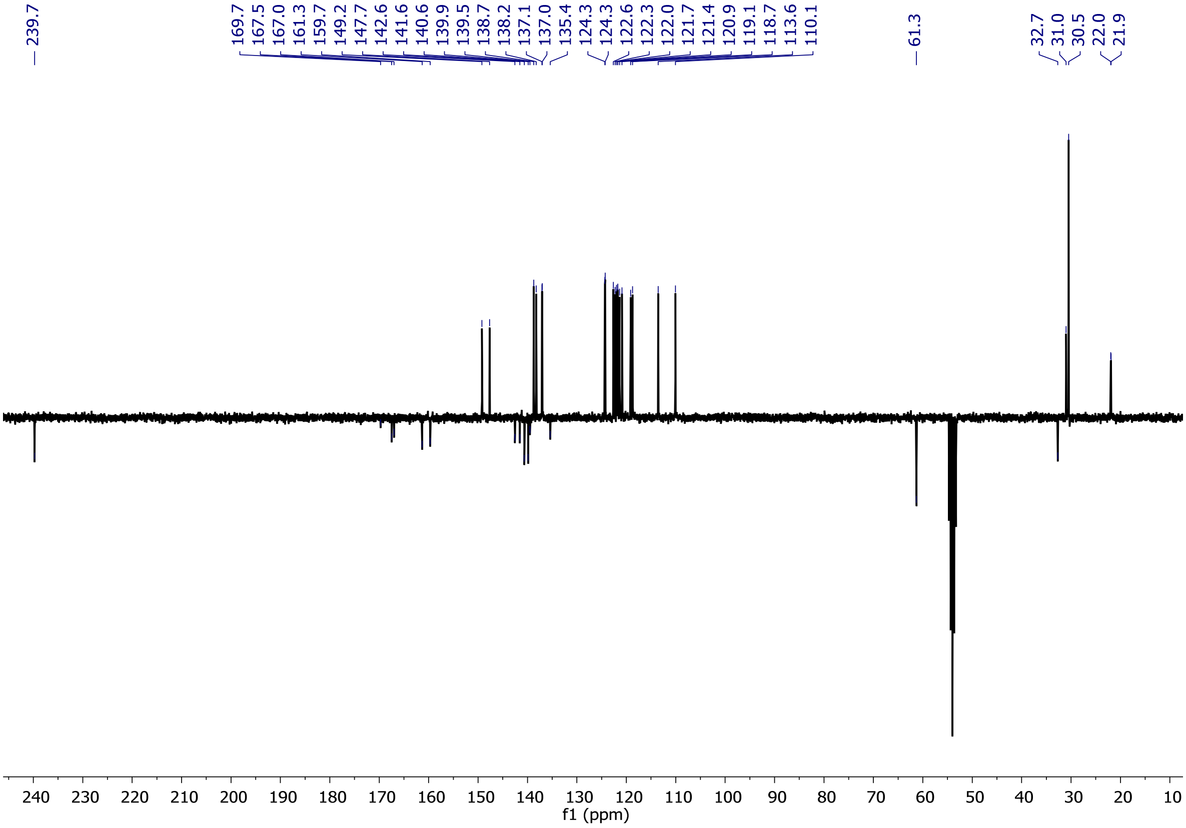

Figure S4. $^{13}C\{^{1}H\}$-apt NMR (75 MHz, CD_2Cl_2, 298 K) of **3**.

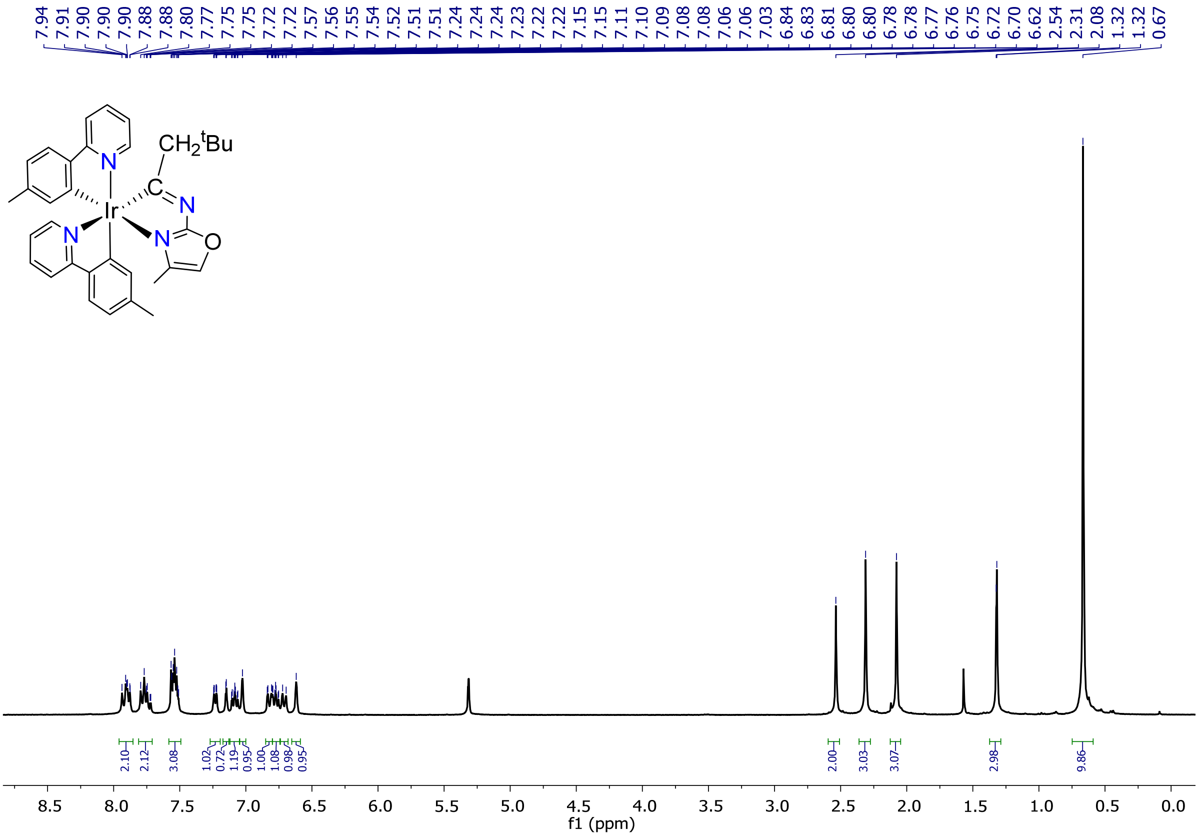

Figure S5. ^{1}H-NMR (300 MHz, CD_2Cl_2, 298 K) of **4**.

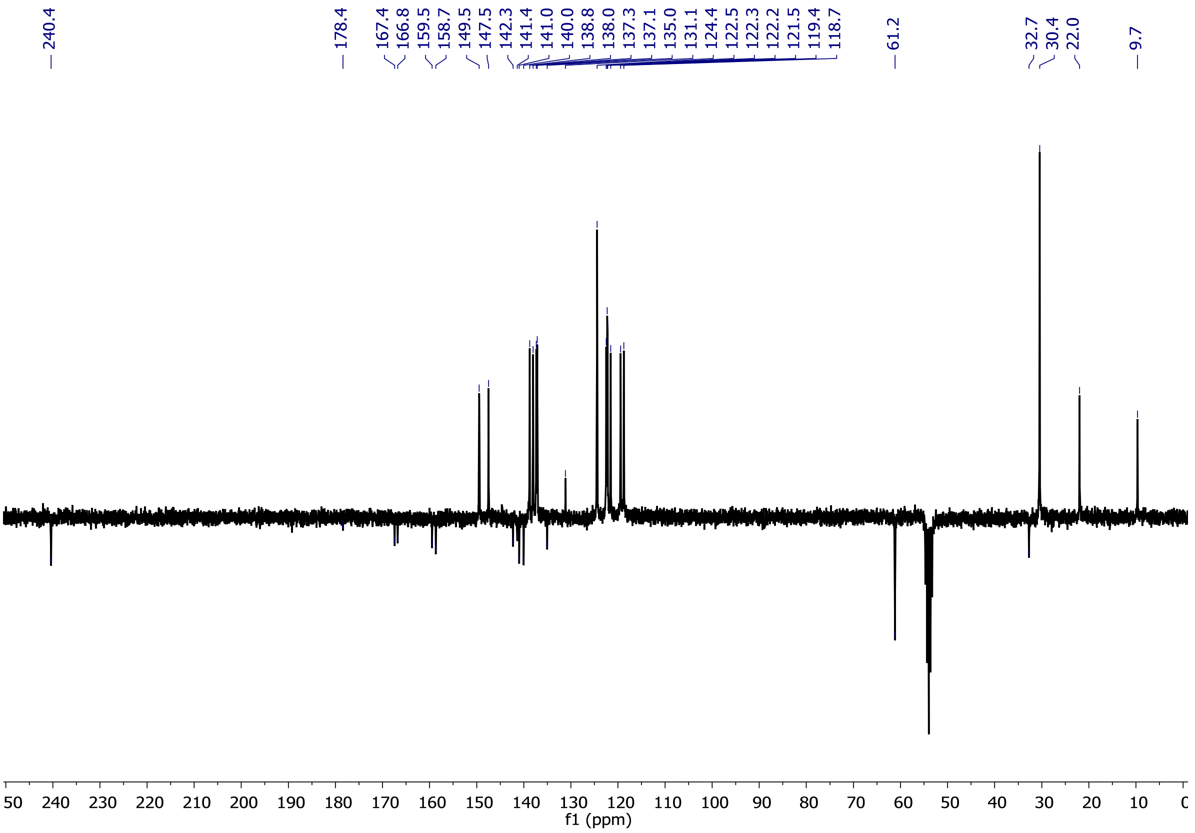

Figure S6. $^{13}C\{^{1}H\}$-apt NMR (75 MHz, CD_2Cl_2, 298 K) of **4**.

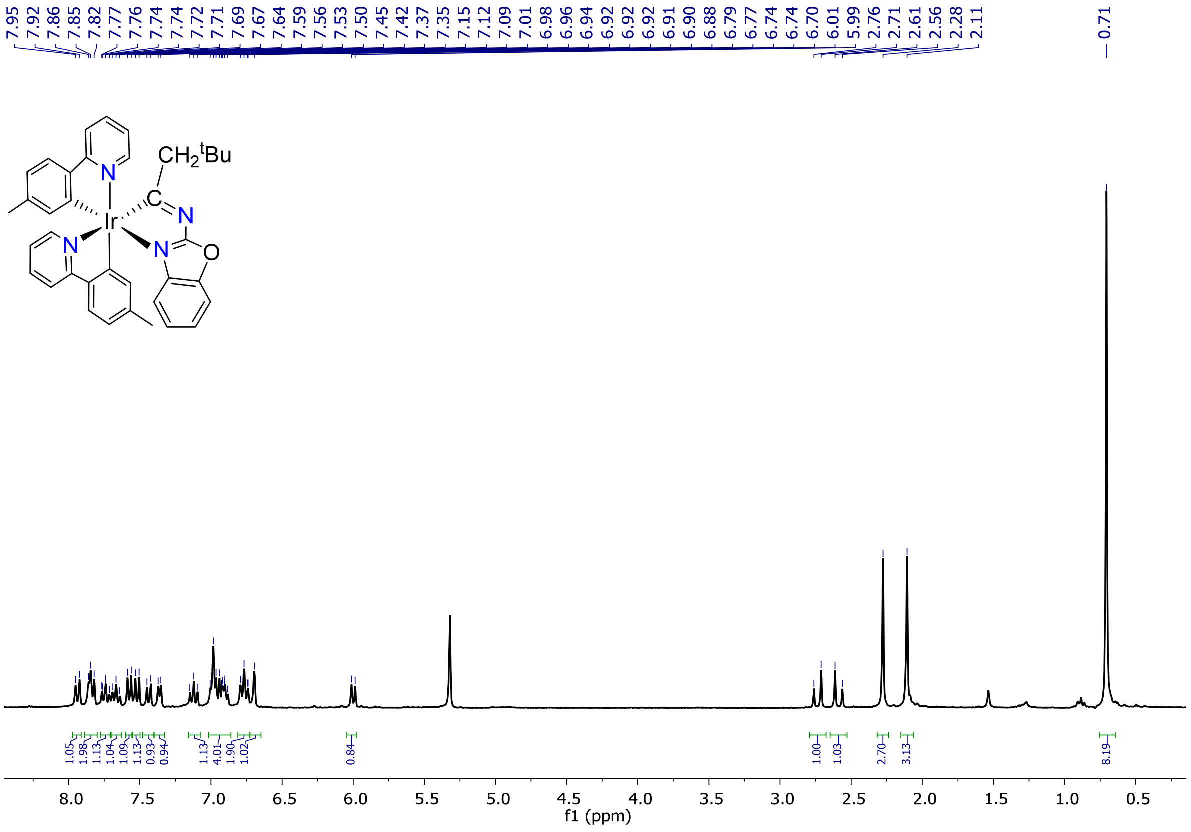

Figure S7. ^{1}H-NMR (300 MHz, CD_2Cl_2, 298 K) of **5**.

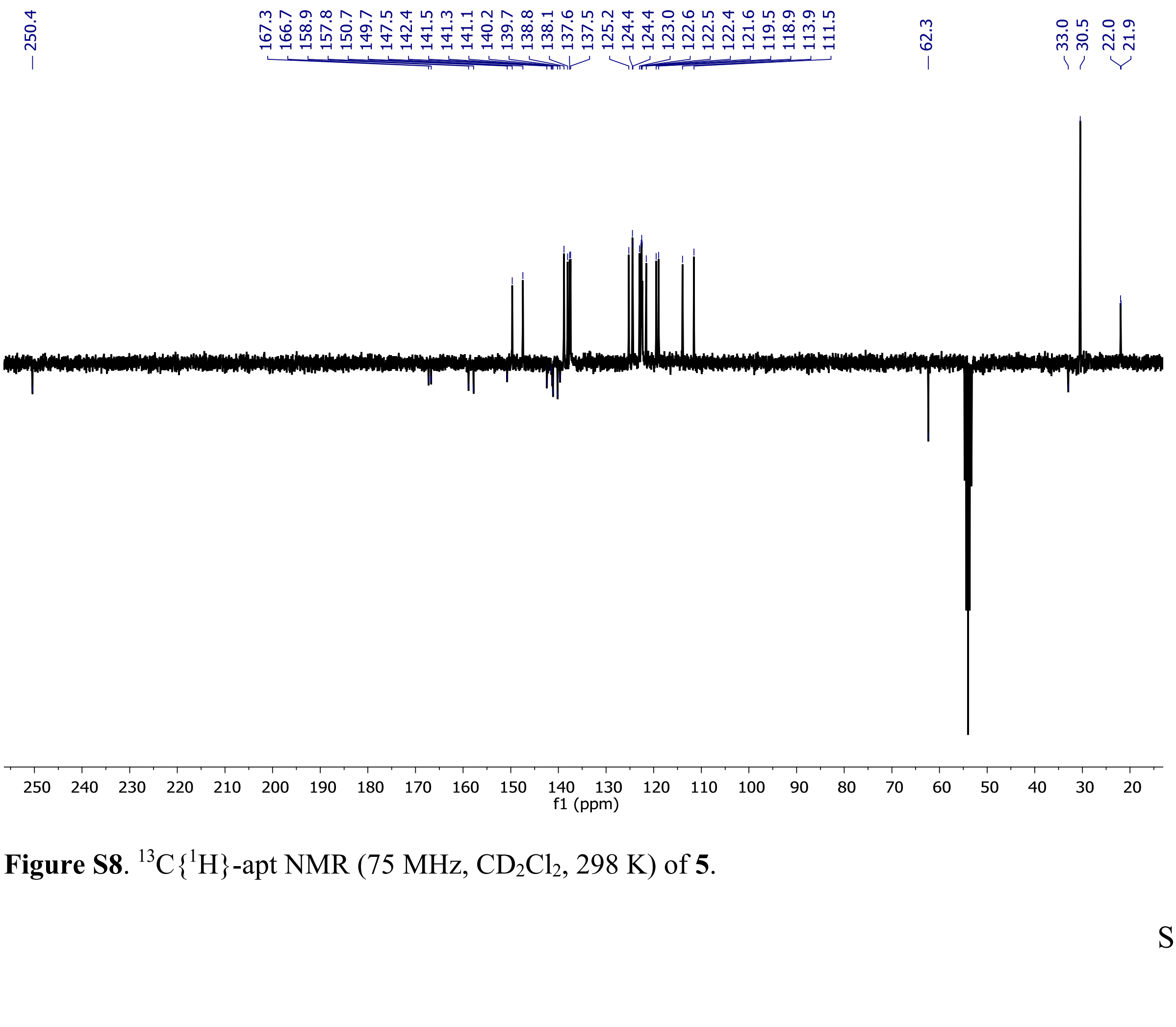

Figure S8. $^{13}C\{^{1}H\}$-apt NMR (75 MHz, CD_2Cl_2, 298 K) of **5**.

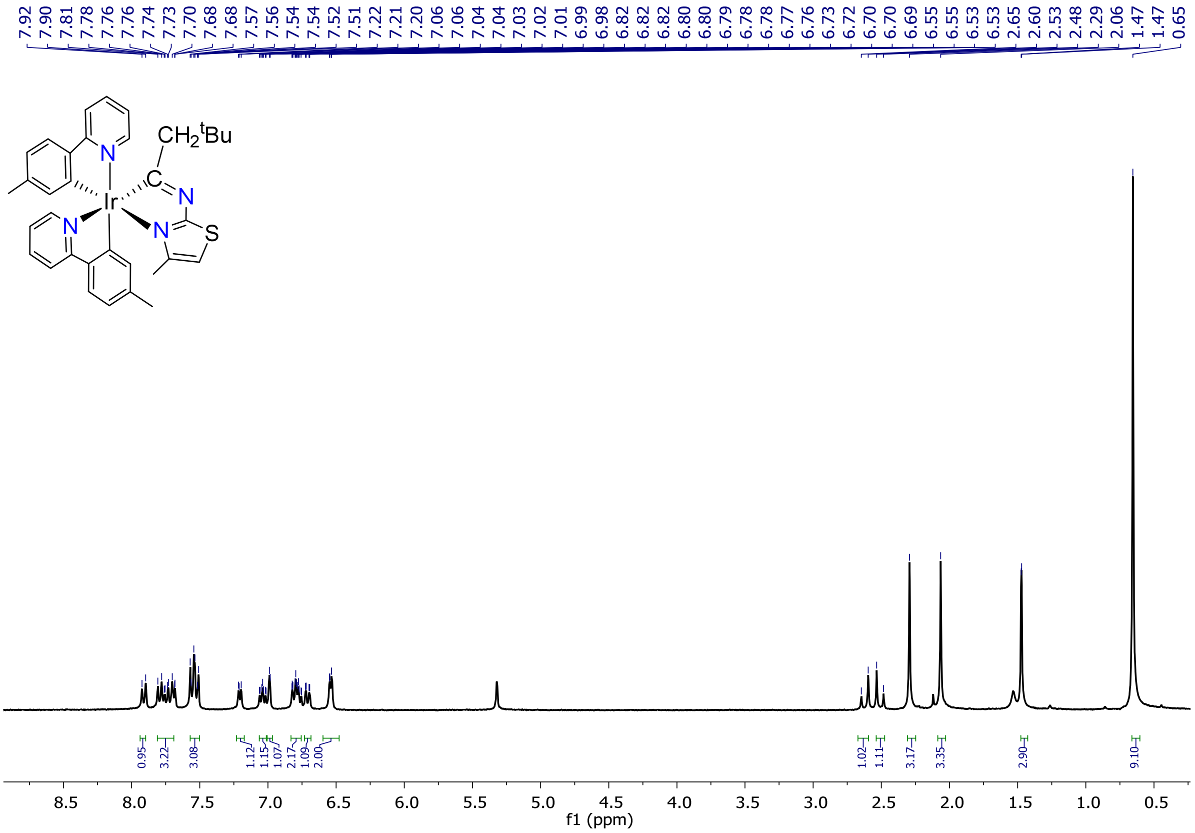

Figure S9. ^{1}H-NMR (300 MHz, CD_2Cl_2, 298 K) of **6**.

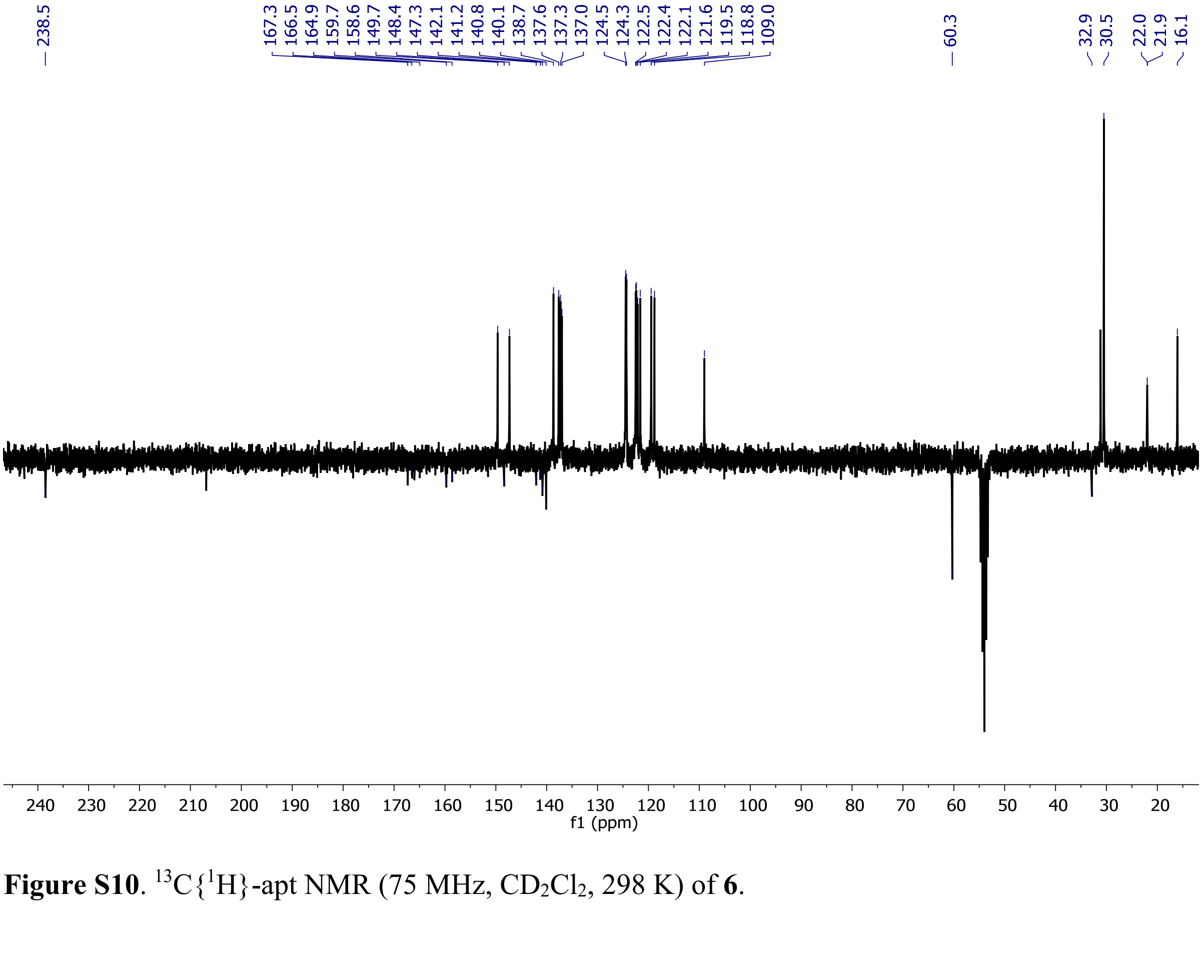

Figure S10. $^{13}C\{^{1}H\}$-apt NMR (75 MHz, CD_2Cl_2, 298 K) of **6**.

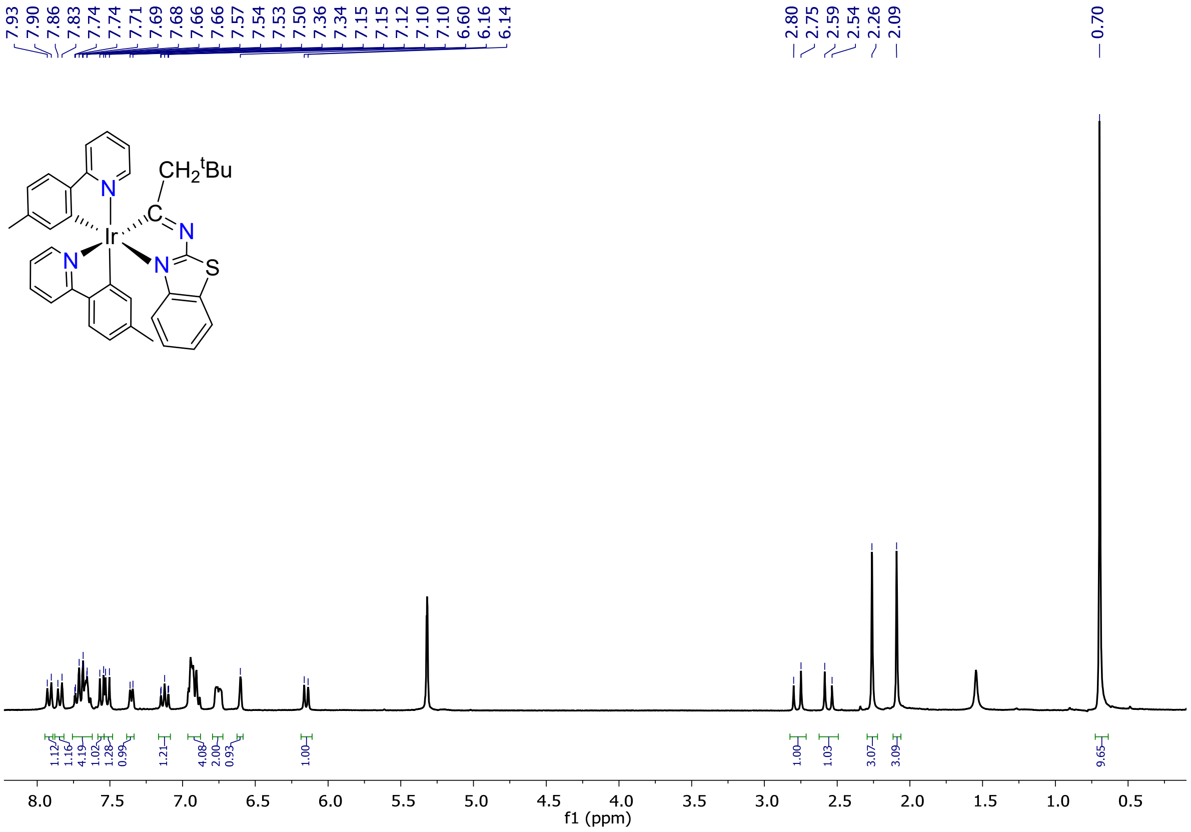

Figure S11. ^{1}H-NMR (300 MHz, CD_2Cl_2, 298 K) of **7**.

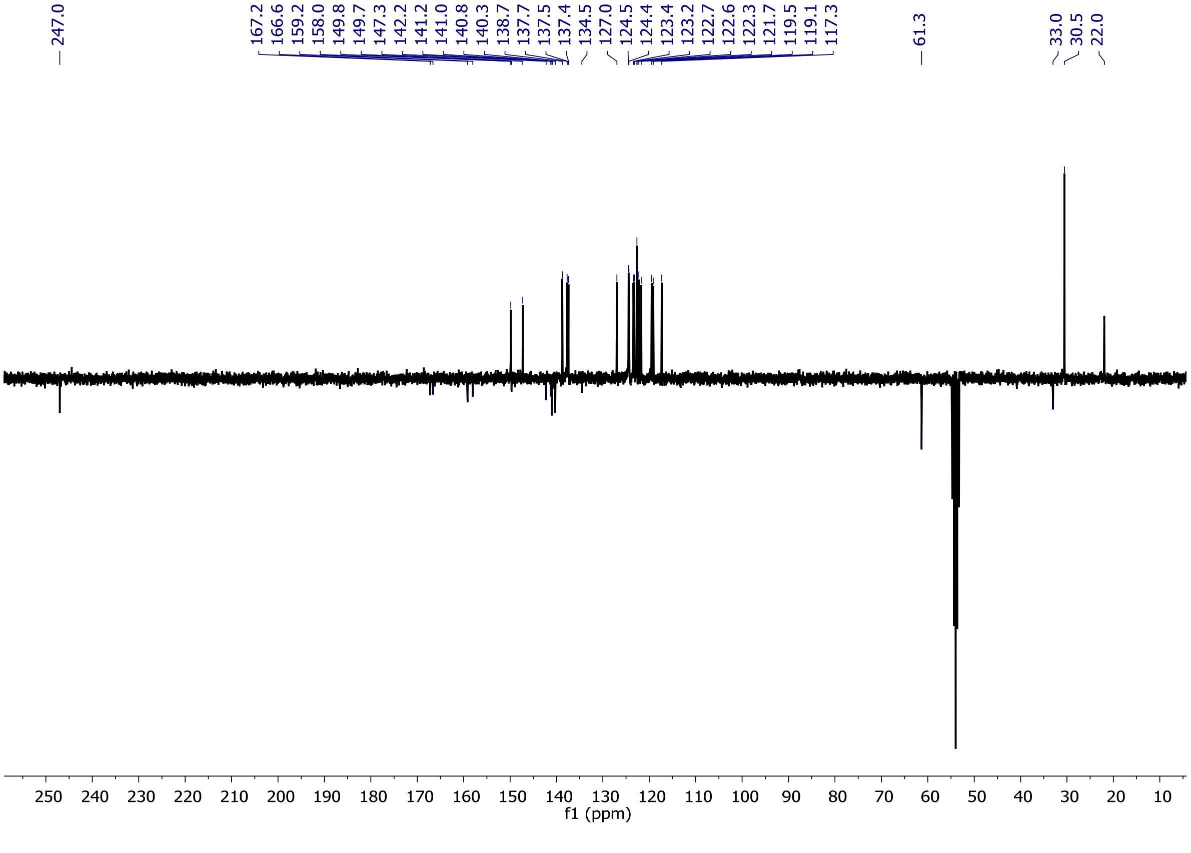

Figure S12. $^{13}C\{^{1}H\}$-apt NMR (75 MHz, CD_2Cl_2, 298 K) of **7**.

Structural Analysis of Complexes 3, 4, and 6. X-ray data were collected on a D8 Venture Bruker diffractometer (Mo radiation, λ = 0.71073 Å). The crystals were mounted under oil in a MiTeGen mount and cooled to 100(2) K with an open-flow nitrogen gas (Oxford Cryosystems). Data were collected for absorption by using a multiscan method applied with the SADABS program.[1] The structures were solved by Patterson or direct methods and refined by full-matrix least squares on F^2 with SHELXL2019,[2] including isotropic and subsequently anisotropic displacement parameters. The hydrogen atoms were observed in the last Fourier Maps or calculated, and refined freely or using a restricted riding model. The disordered groups were refined with two moieties with anisotropic displacement parameters, restrained geometry and complementary occupancy factors.

Crystal data for **3** (CCDC 2492647): $C_{38}H_{38}IrN_5$, Mw 756.93, yellow, irregular block (0.260 x 0.054 x 0.038 mm^3), monoclinic, space group $P2_1/n$, *a*: 11.1677(5) Å, *b*: 18.3663(7) Å, *c*: 15.8214(7) Å, *β*: 101.2265(16)°, *V* = 3183.0(2) Å^3, *Z* = 4, *Z'* = 1, D_{calc}: 1.580 g cm^{-3}, F(000): 1512, T = 100(2) K, μ 4.230 mm^{-1}. 114529 measured reflections (2θ: 3-57°, ω and φ scans 0.5°), 7907 unique (R_{int} = 0.0398); min./max. transm. factors 0.514/0.746. Final agreement factors were R^1 = 0.0180 (7288 observed reflections, I > 2σ(I)) and wR^2 = 0.0451; data/restraints/parameters 7907/0/403; GoF = 1.064. Largest peak and hole 0.923 (close to Ir atoms) and -0.901 e/Å^3.

Crystal data for **4** (CCDC 2492649): $C_{34}H_{35}IrN_4O$, Mw 707.86, violet, irregular block, (0.200 x 0.023 x 0.015 mm^3), monoclinic, space group $P2_1/c$, *a*: 33.5073(13) Å, *b*: 10.1187(4) Å, *c*: 17.3102(6) Å, *β*: 90.8420(14)°, *V* = 5868.4(4) Å^3, *Z* = 8, *Z'* = 2, D_{calc}: 1.602 g cm^{-3}, F(000): 2816, T = 100(2) K, μ 4.584 mm^{-1}. 189369 measured reflections (2θ: 3-57°, ω and φ scans 0.5°), 14640 unique (R_{int} = 0.0753); min./max. transm. factors 0.628/0.746. Final agreement factors were R^1 = 0.0507 (13036 observed reflections, I > 2σ(I)) and wR^2 = 0.1076;

data/restraints/parameters 14640/649/831; GoF = 1.210. Largest peak and hole 2.342 (close to Ir atoms) and -2.918 e/Å^3.

Crystal data for **6** (CCDC 2492648): $C_{34}H_{35}IrN_4S$, M_W 723.92, yellow, irregular block, (0.118 x 0.107 x 0.079 mm^3), monoclinic, space group $P2_1/c$, *a*: 16.5312(11) Å, *b*: 10.5640(11) Å, *c*: 16.7219(11) Å, *β*: 93.111(2)°, V = 2915.9(4) Å^3, Z = 4, Z' = 1, D_{calc}: 1.649 g cm^{-3}, F(000): 1440, T = 100(2) K, μ 4.681 mm^{-1}. 118666 measured reflections (2θ: 3-57°, ω and φ scans 0.5°), 7191 unique (R_{int} = 0.0405); min./max. transm. factors 0.644/0.746. Final agreement factors were R^1 = 0.0193 (6924 observed reflections, I > 2σ(I)) and wR^2 = 0.0504; data/restraints/parameters 7191/0/367; GoF = 1.075. Largest peak and hole 2.237 (close to Ir atoms) and -1.132 e/Å^3.

Computational Details. All calculations were performed at the DFT level using the B3LYP functional[3] supplemented with the Grimme's dispersion correction D3[4] as implemented in Gaussian09.[5] Ir atoms were described by means of an effective core potential SDD for the inner electrons[6] and its associated double-ζ basis set for the outer ones, complemented with a set of f-polarization functions for iridium.[7] The 6-31G** basis set was used for the H, C, N, O and S atoms.[8] All minima were verified to have no negative frequencies. The geometries of singlets and triplets were fully optimized in THF (ε = 7.4257) solvent using the continuum SMD model.[9] We performed TD-DFT calculations at the same level of theory in THF calculating the lowest 50 singlet-singlet excitations at the ground state S_0. It should be noted that the singlet-triplet excitations are set to zero due to the neglect of spin-orbit coupling in the TD-DFT calculations as implemented in G09. The UV/vis absorption spectra were obtained by using the GaussSum 3 software.[10] The phosphorescence emission compares well with the 0-0 transition calculated taking into account the zero point energies (zpe) of the geometries of both the optimized T_1 and S_0 states in THF. In the mechanistic studies, the transition states were identified by having one imaginary frequency in the Hessian matrix. It was confirmed that transition states connect with

the corresponding intermediates by means of application of an eigenvector corresponding to the imaginary frequency and subsequent optimization of the resulting structures. Gibbs energies were computed at 298.15 K and 1 atmosphere. All values collected in Figure S13 correspond to Gibbs energies (kcal mol^{-1}) in toluene (ε = 2.3741).

The Cartesian coordinates for the computed structures can be found in the supplemental file xyz. The file may be opened as a text file to read the coordinates, or opened directly by a molecular modeling program such as Mercury (version 3.3 or later, http://www.ccdc.cam.ac.uk/pages/Home.aspx) for visualization and analysis.

Energies of Optimized Structures

**Compound**	**Sum of electronic and thermal Free Energies (Hartree/Molecule)**
**A**	-1695.18583
**TS_{AB}**	-1695.140936
**B**	-1695.151024
**TS_{BC}**	-1695.149034
**C**	-1695.193165
**TS_{C2}**	-1695.124222
**2**	-1695.229712
**D**	-2016.033987
**TS_{DE}**	-2016.00266
**E**	-2016.029703
1-methyl-1*H*-imidazol-2-amine (ligand model)	-320.834044

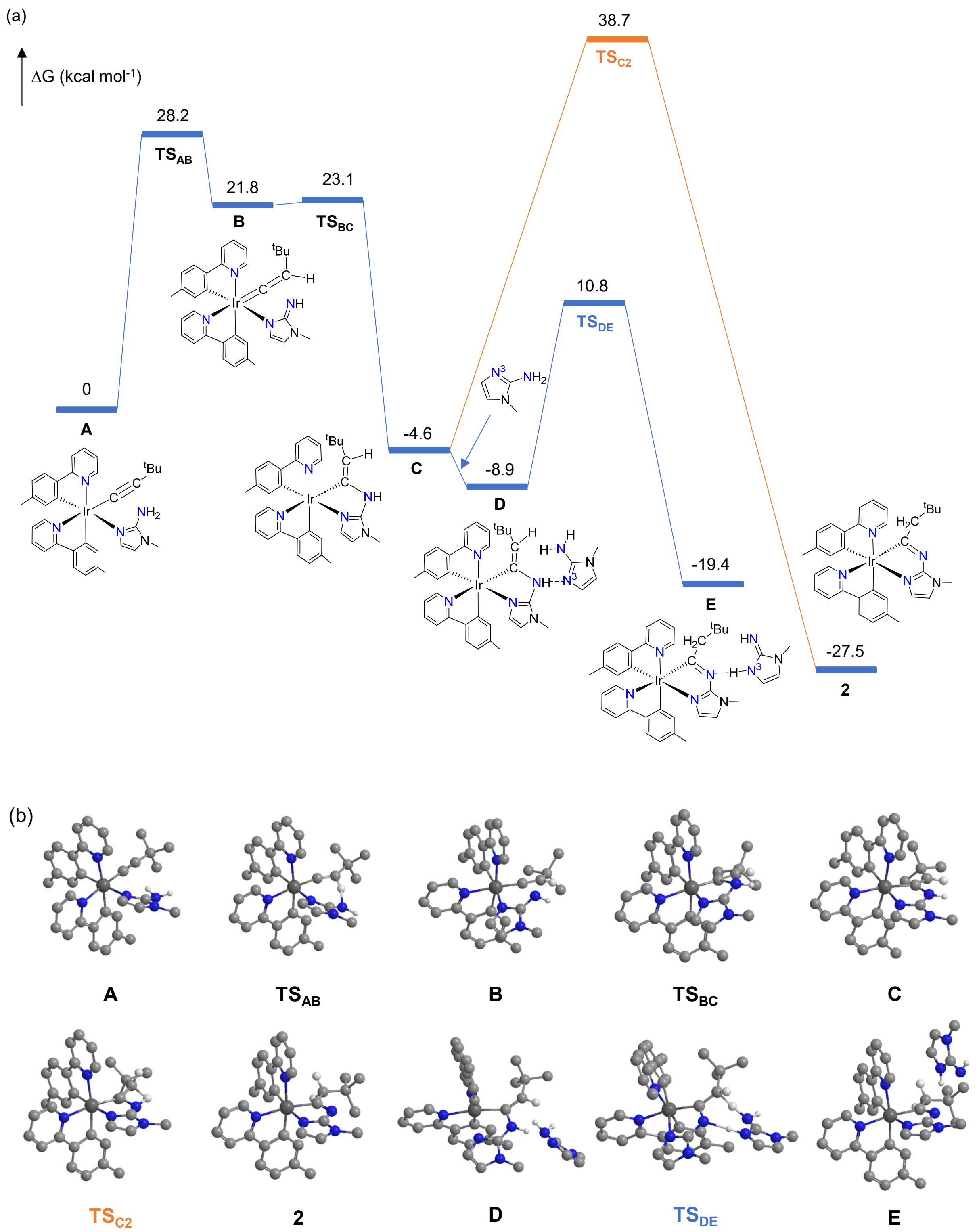

Figure S13. (a) Computed energy profile for the formation of the iridaimidazole complex **2** via direct (orange lines) or amine-assisted (blue lines) 1,3-hydrogen shift. (b) Optimized structures of intermediates and transition states. Hydrogen atoms have been omitted except for those from the NH_2 amine group.

Energies of Optimized Structures of 2−7

Complex 2-S_0(THF)

Zero-point correction= 0.627345 (Hartree/Particle)
Thermal correction to Energy= 0.665297
Thermal correction to Enthalpy= 0.666241
Thermal correction to Gibbs Free Energy= 0.554614
Sum of electronic and zero-point Energies= -1695.159046
Sum of electronic and thermal Energies= -1695.121094
Sum of electronic and thermal Enthalpies= -1695.120150
Sum of electronic and thermal Free Energies= -1695.231777

Complex 2-T_1(THF)

Zero-point correction= 0.623232 (Hartree/Particle)
Thermal correction to Energy= 0.661835
Thermal correction to Enthalpy= 0.662780
Thermal correction to Gibbs Free Energy= 0.548840
Sum of electronic and zero-point Energies= -1695.068468
Sum of electronic and thermal Energies= -1695.029864
Sum of electronic and thermal Enthalpies= -1695.028920
Sum of electronic and thermal Free Energies= -1695.142860

Complex 3-S_0(THF)

Zero-point correction= 0.674322 (Hartree/Particle)
Thermal correction to Energy= 0.714845
Thermal correction to Enthalpy= 0.715790
Thermal correction to Gibbs Free Energy= 0.599362
Sum of electronic and zero-point Energies= -1848.782217
Sum of electronic and thermal Energies= -1848.741694
Sum of electronic and thermal Enthalpies= -1848.740750
Sum of electronic and thermal Free Energies= -1848.857178

Complex 3-T_1(THF)

Zero-point correction= 0.670589 (Hartree/Particle)
Thermal correction to Energy= 0.711627
Thermal correction to Enthalpy= 0.712571
Thermal correction to Gibbs Free Energy= 0.594660
Sum of electronic and zero-point Energies= -1848.690780
Sum of electronic and thermal Energies= -1848.649742
Sum of electronic and thermal Enthalpies= -1848.648798
Sum of electronic and thermal Free Energies= -1848.766709

Complex 4-S_0(THF)

Zero-point correction= 0.615415 (Hartree/Particle)
Thermal correction to Energy= 0.652823
Thermal correction to Enthalpy= 0.653767
Thermal correction to Gibbs Free Energy= 0.544505
Sum of electronic and zero-point Energies= -1715.027213
Sum of electronic and thermal Energies= -1714.989806
Sum of electronic and thermal Enthalpies= -1714.988861
Sum of electronic and thermal Free Energies= -1715.098124

Complex 4-T_1(THF)

Zero-point correction= 0.611558 (Hartree/Particle)
Thermal correction to Energy= 0.649451
Thermal correction to Enthalpy= 0.650395
Thermal correction to Gibbs Free Energy= 0.539676
Sum of electronic and zero-point Energies= -1714.934797
Sum of electronic and thermal Energies= -1714.896905
Sum of electronic and thermal Enthalpies= -1714.895961
Sum of electronic and thermal Free Energies= -1715.006680

Complex 5-S_0(THF)

Zero-point correction= 0.633720 (Hartree/Particle)
Thermal correction to Energy= 0.672330
Thermal correction to Enthalpy= 0.673275
Thermal correction to Gibbs Free Energy= 0.560281
Sum of electronic and zero-point Energies= -1829.348380
Sum of electronic and thermal Energies= -1829.309769
Sum of electronic and thermal Enthalpies= -1829.308825
Sum of electronic and thermal Free Energies= -1829.421819

Complex 5-T_1(THF)

Zero-point correction= 0.629764 (Hartree/Particle)
Thermal correction to Energy= 0.668957
Thermal correction to Enthalpy= 0.669901
Thermal correction to Gibbs Free Energy= 0.554793
Sum of electronic and zero-point Energies= -1829.255831
Sum of electronic and thermal Energies= -1829.216639
Sum of electronic and thermal Enthalpies= -1829.215694
Sum of electronic and thermal Free Energies= -1829.330802

Complex 6-S_0(THF)

Zero-point correction= 0.611937 (Hartree/Particle)
Thermal correction to Energy= 0.649836
Thermal correction to Enthalpy= 0.650780
Thermal correction to Gibbs Free Energy= 0.540282

Sum of electronic and zero-point Energies= -2038.009114
Sum of electronic and thermal Energies= -2037.971215
Sum of electronic and thermal Enthalpies= -2037.970271
Sum of electronic and thermal Free Energies= -2038.080769

Complex 6-T_1(THF)

Zero-point correction= 0.608042 (Hartree/Particle)
Thermal correction to Energy= 0.646687
Thermal correction to Enthalpy= 0.647631
Thermal correction to Gibbs Free Energy= 0.533382
Sum of electronic and zero-point Energies= -2037.924935
Sum of electronic and thermal Energies= -2037.886290
Sum of electronic and thermal Enthalpies= -2037.885345
Sum of electronic and thermal Free Energies= -2037.999595

Complex 7-S_0(THF)

Zero-point correction= 0.630612 (Hartree/Particle)
Thermal correction to Energy= 0.669788
Thermal correction to Enthalpy= 0.670732
Thermal correction to Gibbs Free Energy= 0.556960
Sum of electronic and zero-point Energies= -2152.330156
Sum of electronic and thermal Energies= -2152.290980
Sum of electronic and thermal Enthalpies= -2152.290036

Complex 7-T_1(THF)

Zero-point correction= 0.627645 (Hartree/Particle)
Thermal correction to Energy= 0.667465
Thermal correction to Enthalpy= 0.668410
Thermal correction to Gibbs Free Energy= 0.551525
Sum of electronic and zero-point Energies= -2152.244848
Sum of electronic and thermal Energies= -2152.205029
Sum of electronic and thermal Enthalpies= -2152.204084
Sum of electronic and thermal Free Energies= -2152.320969

NICS and $NICS_{zz}$ for complexes 2−7

Table S1. NICS and $NICS_{zz}$ for the iridaimidazole ring of complexes **2−7**

Complex	NICS			$NICS_{zz}$		
	0	1	-1	0	1	-1
**2**	0.8322	-0.7526	-1.2279	22.3994	-0.6123	-0.6020
**3**	0.9892	-0.6323	-1.2265	23.5670	-0.1481	-0.1530
**4**	0.3070	-1.7880	-1.0311	20.4477	-1.8681	-0.7394
**5**	0.4261	-1.0056	-1.5655	21.2226	-0.9992	-1.0250
**6**	2.6025	-1.2020	-0.7508	23.2042	-1.6204	-0.2258
**7**	0.8414	-0.8867	-1.4316	19.2043	-1.0141	-2.5435

Induced current density (AICD) plots of complexes 2−7

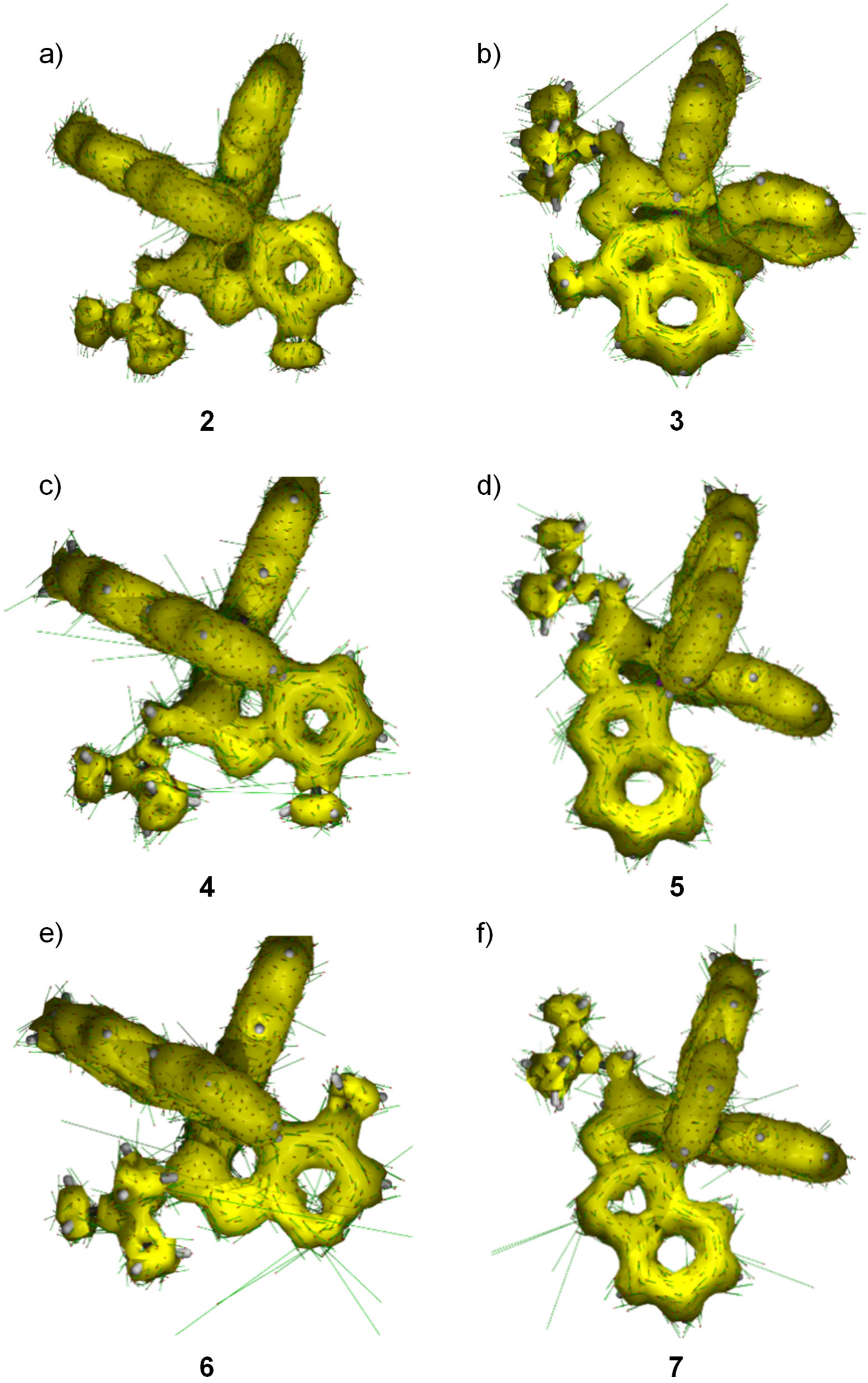

Figure S14. AICD plots of complexes **2** (a), **3** (b), **4** (c), **5** (d), **6** (e), and **7** (f) with isosurface values of 0.03.

UV-vis Spectra of Complexes 2−7 (Observed and Calculated)

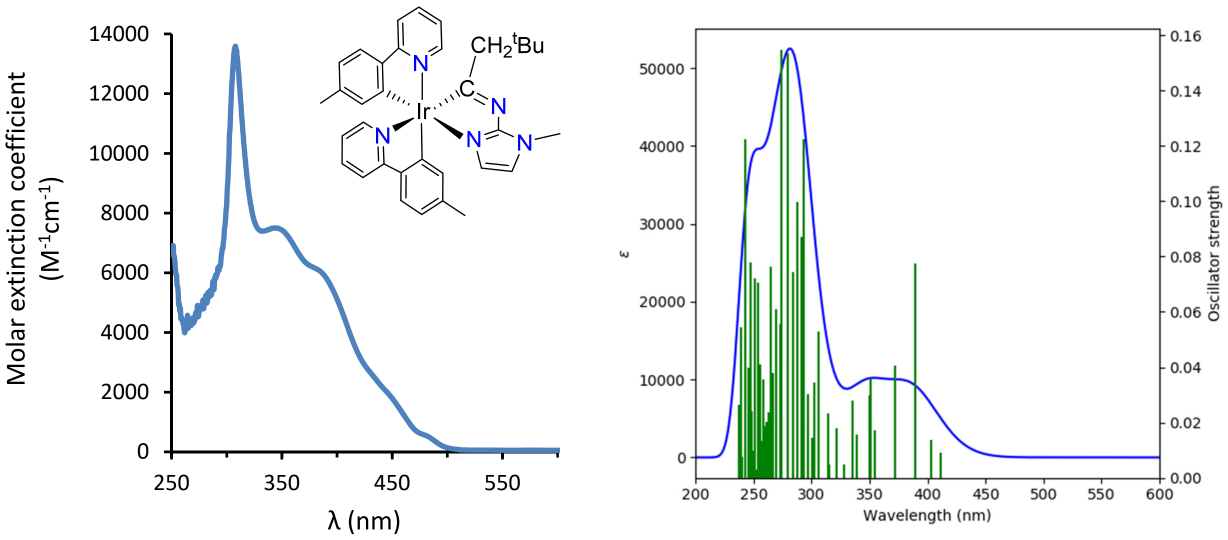

Figure S15. Observed UV-vis spectrum of complex **2** in 2-MeTHF (1.0 x 10^{-4} M) and calculated (B3LYP-D3//SDD(f)/6-31G**) in THF.

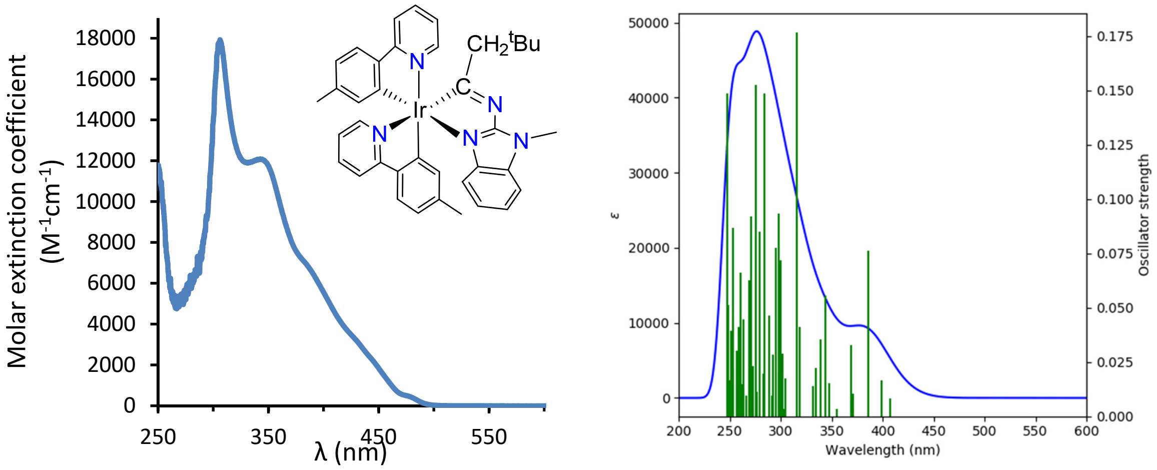

Figure S16. Observed UV-vis spectrum of complex **3** in 2-MeTHF (1.0 x 10^{-4} M) and calculated (B3LYP-D3//SDD(f)/6-31G**) in THF.

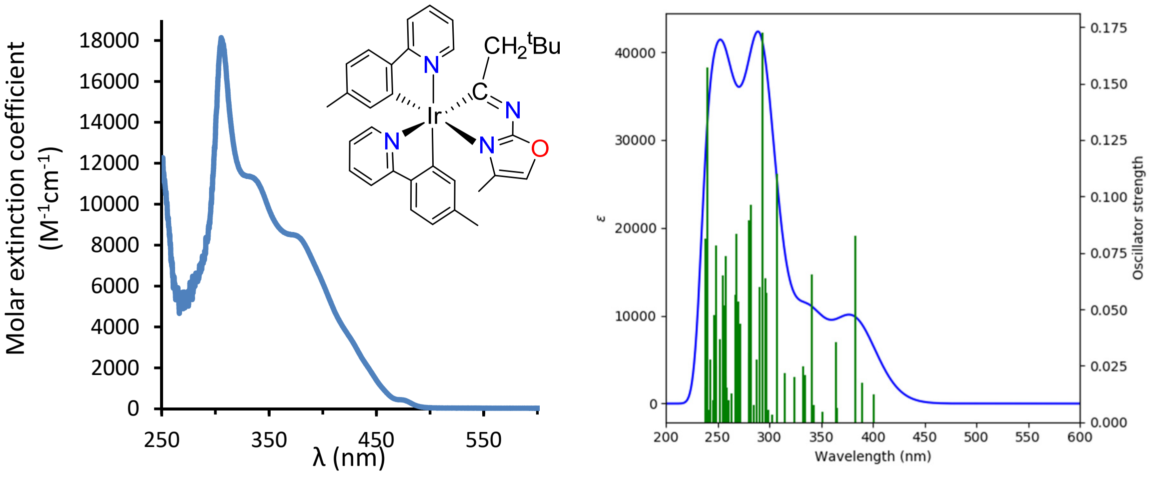

Figure S17. Observed UV-vis spectrum of complex **4** in 2-MeTHF (1.0 x 10^{4} M) and calculated (B3LYP-D3//SDD(f)/6-31G**) in THF.

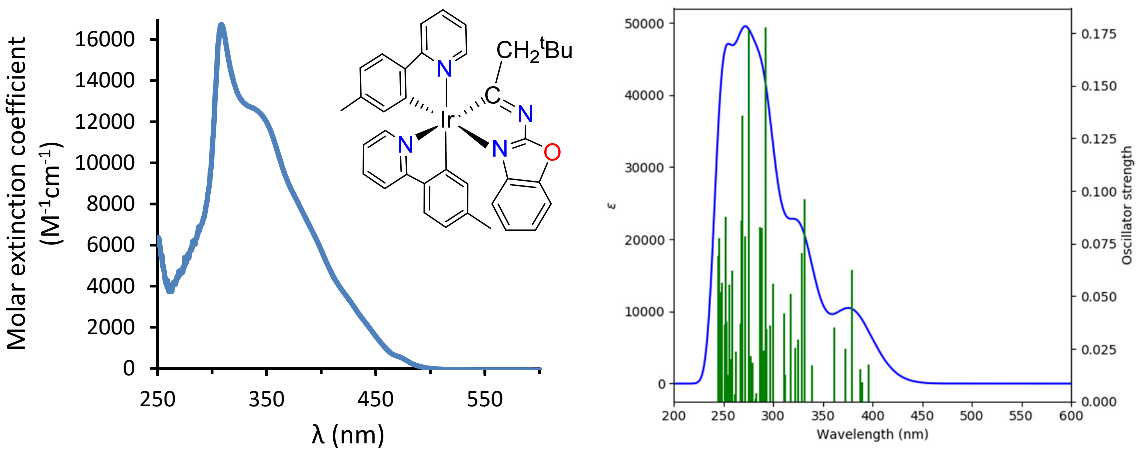

Figure S18. Observed UV-vis spectrum of complex **5** in 2-MeTHF (1.0 x 10^{-4} M) and calculated (B3LYP-D3//SDD(f)/6-31G**) in THF.

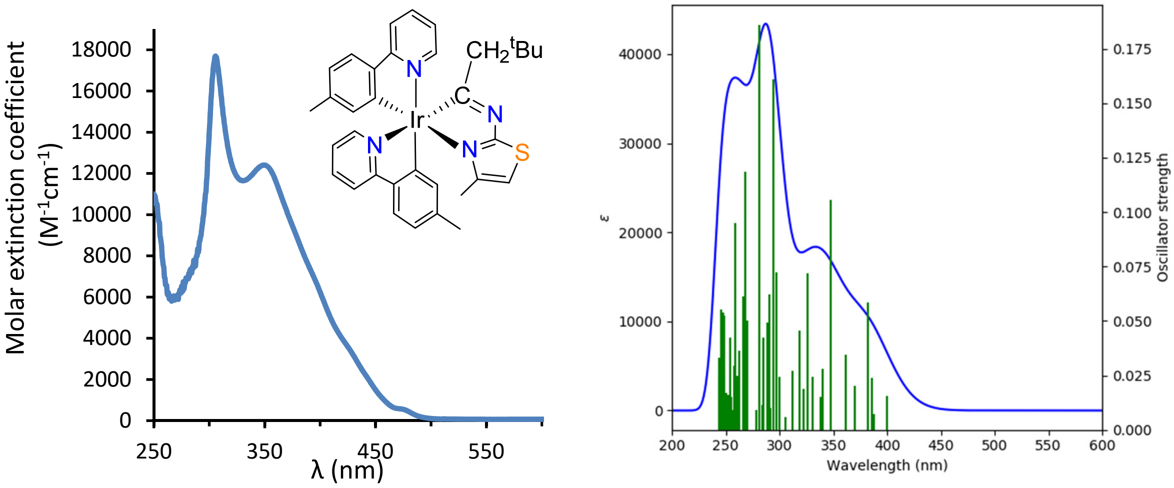

Figure S19. Observed UV-vis spectrum of complex **6** in 2-MeTHF (1.0 x 10^{-4} M) and calculated (B3LYP-D3//SDD(f)/6-31G**) in THF.

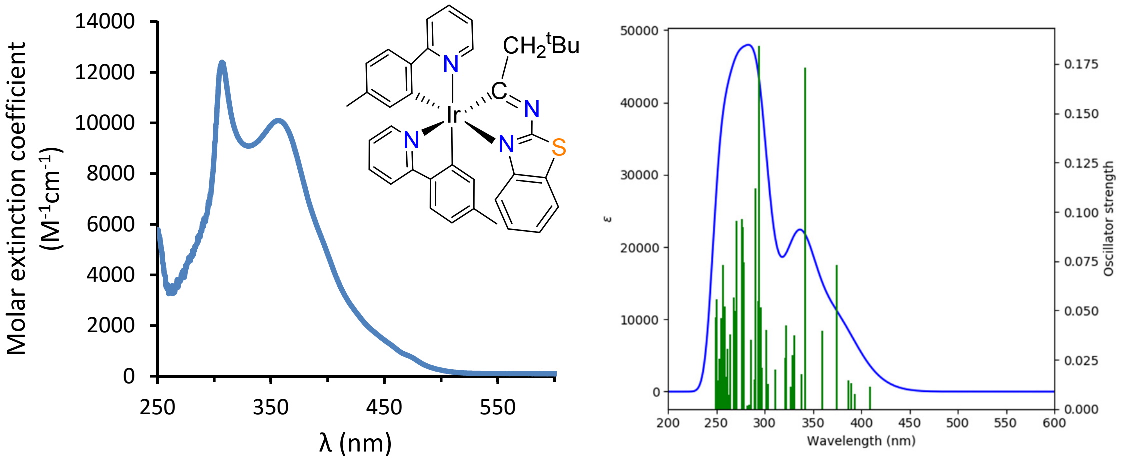

Figure S20. Observed UV-vis spectrum of complex **7** in 2-MeTHF (1.0 x 10^{-4} M) and calculated (B3LYP-D3//SDD(f)/6-31G**) in THF.

Analysis of Computed UV/Vis Data of Complexes 2–7

Selected transitions for the calculated UV spectra, oscillator strengths, and molecular orbital contributions to the transitions are given in Tables S2–S7.

Table S2. Selected transitions for the calculated UV spectrum of complex 2 in THF

λ (nm)	Osc. Strength	Symmetry	Major contributions	Minor contributions
457	0	Triplet-A	HOMO->LUMO (32%), HOMO->L+1 (31%), H-2->LUMO (19%)	H-1->LUMO (8%), H-4->L+1 (7%), H-2->L+1 (7%)
411	0.009	Singlet-A	HOMO->LUMO (96%)	-
403	0.0138	Singlet-A	HOMO->L+1 (95%)	H-1->L+1 (2%)
389	0.0773	Singlet-A	H-1->LUMO (93%)	-
372	0.0406	Singlet-A	H-1->L+1 (88%)	H-2->LUMO (4%), HOMO->L+1 (2%)
351	0.0359	Singlet-A	H-2->L+1 (26%), HOMO->L+2 (64%)	H-2->LUMO (5%)
306	0.0528	Singlet-A	H-3->LUMO (83%)	H-4->LUMO (5%), H-5->LUMO (4%), H-6->LUMO (2%)
274	0.1548	Singlet-A	H-3->L+2 (59%), HOMO->L+5 (12%)	H-6->LUMO (7%), H-5->L+4 (3%), H+4->L+2 (3%), H-6->L+1 (2%), H-3-> L+4 (2%)
270	0.0611	Singlet-A	H-4->L+2 (74%)	H-4->L+3 (4%), H-3->L+2 (4%), H-8->LUMO (2%), H-6->L+1 (2%)

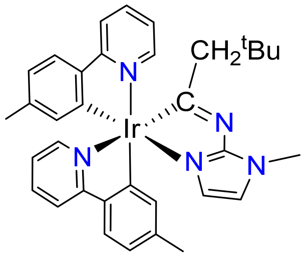

Table S3. Selected transitions for the calculated UV spectrum of complex 3 in THF

λ (nm)	Osc. Strength	Symmetry	Major contributions	Minor contributions
454	0	Triplet-A	HOMO->L+1 (44%), HOMO->LUMO (23%)	H-3->L+1 (7%), H-2->L+1 (5%), H-1->LUMO (5%), H-3->LUMO (3%)
407	0.0083	Singlet-A	HOMO->LUMO (96%)	
386	0.076	Singlet-A	H-1->LUMO (94%)	
369	0.0329	Singlet-A	H-1->L+1 (91%)	HOMO->L+1 (3%)
348	0.0151	Singlet-A	HOMO->L+2 (18%), HOMO->L+3 (73%)	H-2->LUMO (5%)
344	0.0556		H-2->LUMO (68%), H-2->L+1 (15%)	H-1->L+3 (5%), HOMO->L+3 (4%)
316	0.1768	Singlet-A	H-2->L+2 (71%), H-1->L+4 (12%)	H-2-> L+3 (3%)
300	0.0717	Singlet-A	H-4->L+1 (11%), H-3-> L+1 (67%)	H-4->LUMO (5%), H-6->L+1 (4%), H-2->L+1 (3%), H-5->LUMO (2%)
279	0.085	Singlet-A	H-3->L+2 (73%)	H-3->L+3 (4%), H-4->L+2 (4%), H-6->L+1 (3%), H-4->L+3 (2%)
275	0.1524	Singlet-A	H-4->L+2 (48%), H-7->L+1 (18%)	H-6->LUMO (6%), H-7->LUMO (5%), H-5->L+2 (4%), H-3->L+3 (4%)

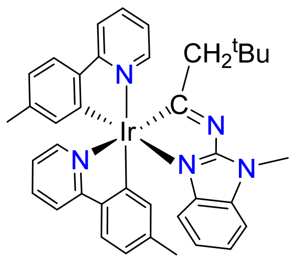

Table S4. Selected transitions for the calculated UV spectrum of complex 4 in THF

λ (nm)	Osc. Strength	Symmetry	Major contributions	Minor contributions
451	0	Triplet-A	HOMO->LUMO (29%),<br>HOMO->L+1 (28%),<br>H-1->LUMO (12%)	H-2->L+1 (8%),<br>H-3->L+1 (5%),<br>H-4->LUMO (4%)
401	0.0125	Singlet-A	HOMO->LUMO (94%)	H-1->LUMO (4%)
390	0.0176	Singlet-A	HOMO->L+1 (91%)	H-1->L+1 (6%)
383	0.0825	Singlet-A	H-1->LUMO (92%)	HOMO->LUMO (4%)
365	0.0354	Singlet-A	H-1->L+1 (72%),<br>HOMO-> L+3 (11%)	HOMO->L+2 (6%),<br>HOMO->L+1 (4%)
341	0.0656	Singlet-A	H-2->LUMO (58%),<br>HOMO->L+2 (21%)	HOMO->LUMO (8%),<br>HOMO->L+1 (3%)
293	0.1727	Singlet-A	H-3->L+1 (73%),<br>H-2->L+4 (11%)	H-4->L+1 (3%)
291	0.0598	Singlet-A	H-4->L+1 (80%)	H-3->L+1 (5%),<br>H-5->L+1 (3%),<br>H-2->L+2 (2%)

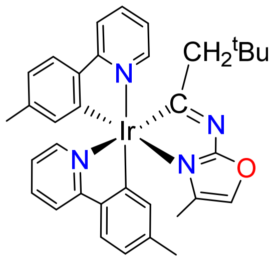

Table S5. Selected transitions for the calculated UV spectrum of complex 5 in THF

λ (nm)	Osc. Strength	Symmetry	Major contributions	Minor contributions
449	0	Triplet-A	HOMO->LUMO (32%), HOMO->L+1 (22%), H-1->LUMO (12%)	H+2 ->L+1 (7%), H-4->L+1 (5%), H+1->L+1 (4%), H-3->LUMO (3%)
396	0.0176	Singlet-A	HOMO->LUMO (92%)	HOMO->L+2 (4%)
379	0.0626	Singlet-A	H-1->LUMO (84%), H-1->L+2 (11%)	
339	0.0172	Singlet-A	HOMO->L+3 (92%)	
332	0.0959	Singlet-A	H-2->LUMO (62%), H-1->L+3 (22%)	H+2->L+2 (6%), H-4->LUMO (3%)
329	0.0706	Singlet-A	H-1->L+3 (65%), H-2->LUMO (13%), H-2->L+2 (12%)	H-2->L+1 (5%), H-3->L+1 (3%)
292	0.1778	Singlet-A	H-4->L+1 (65%), H-3->L+1 (11%)	H-2->L+1 (4%), H-5->L+1 (3%), H-4->L+2 (3%), H-2->L+4 (3%)
287	0.083	Singlet-A	H-3->L+2 (62%)	H-5->LUMO (8%), H-4->L+2 (7%), H-6->LUMO (3%), H-3->LUMO (3%), H-3->L+1 (3%), H-2->L+3 (2%)
268	0.0857	Singlet-A	H-3->L+3 (49%), H-5->L+2 (21%), H-6->L+2 (12%)	H-4->L+3 (5%)

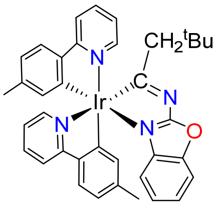

Table S6. Selected transitions for the calculated UV spectrum of complex 6 in THF

λ (nm)	Osc. Strength	Symmetry	Major contributions	Minor contributions
462	0	Triplet-A	HOMO->L+2 (47%), H-2->L+2 (24%), HOMO->LUMO (11%),	H-6->L+2 (5%), H-2->LUMO (3%)
400	0.0157	Singlet-A	HOMO->LUMO (87%)	HOMO->L+2 (7%), H-1->LUMO (4%)
382	0.0584	Singlet-A	H-1->L+1 (80%)	H-1->L+2 (7%), HOMO-> L+1 (6%), HOMO->LUMO (4%)
347	0.1057	Singlet-A	H-2->LUMO (89%)	H-2->L+2 (3%)
319	0.0458	Singlet-A	H-5->L+2 (52%), H-2->L+2 (20%), H-5->LUMO (13%)	H-4->L+2 (4%), H-6->L+2 (2%)
297	0.0723	Singlet-A	H-4->LUMO (79%)	H-5->L+2 (2%), H-3->L+1 (4%), H-2 ->L+3 (5%), H-2 ->L+4 (3%)
294	0.1611	Singlet-A	H-3->L+1 (68%), H-2->L+4 (14%)	H-5->LUMO (6%)

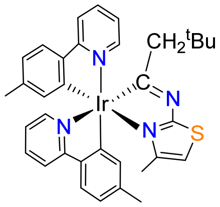

Table S7. Selected transitions for the calculated UV spectrum of complex 7 in THF

λ (nm)	Osc. Strength	Symmetry	Major contributions	Minor contributions
459	0	Triplet-A	HOMO->LUMO (35%), HOMO->L+1 (34%)	H-2->LUMO (8%), H-2->L+1 (7%), H-6->LUMO (3%), H-6->L+1 (3%)
409	0.0113	Singlet-A	HOMO->L+1 (58%), HOMO->LUMO (13%)	
374	0.073	Singlet-A	H-1->L+1 (60%), H-1->LUMO (33%)	
360	0.0395	Singlet-A	H-1->L+2 (91%)	H-2->L+1 (3%)
342	0.1732	Singlet-A	H-2->LUMO (66%)	H-2->L+1 (9%), H-5->LUMO (5%), H-5->L+1 (5%), HOMO->L+3 (4%), H-4->LUMO (2%), H-3->LUMO (2%)
294	0.1843	Singlet-A	H-4->L+2 (40%), H-3->L+2 (22%), H-4->L+1 (19%)	H-5->L+2 (2%), H-2->L+2 (2%)
293	0.0546	Singlet-A	H-3->L+2 (41%), H-4->L+2 (39%)	H-2->L+3 (8%), H-3->L+1 (4%)
291	0.1119	Singlet-A	H-2->L+3 (48%), H-3->L+1 (11%)	H-5->L+1 (7%), H-4->L+2 (7%), H-5->LUMO (6%), H-4->L+1 (6%), H-3->LUMO (3%), H-3->L+2 (2%), H-2->L+4 (2%)

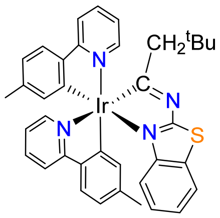

Theoretical Analysis of Molecular Orbitals of Complexes 2–7

Energies and population analysis (%) of molecular orbitals are given in Tables S8–S13 whereas Figures S21–S26 collect the frontier molecular orbitals.

Table S8. Composition (%) of the molecular orbitals of complex 2

MO	eV	Iridium	*p*-tol-py (3b-1)	*p*-tol-py (3b-2)	L (3b')
L+9	1.15	32	33	2	33
L+8	1.05	10	33	49	7
L+7	0.63	6	54	40	1
L+6	0.54	7	39	52	1
L+5	0.15	95	1	1	3
L+4	-0.22	10	1	4	85
L+3	-0.56	3	62	34	1
L+2	-0.75	3	35	61	1
L+1	-1.09	5	72	23	1
LUMO	-1.19	3	24	73	0
HOMO	-4.95	47	29	6	18
H-1	-5.09	42	7	43	8
H-2	-5.33	30	18	4	48
H-3	-5.83	11	5	54	30
H-4	-5.89	11	76	6	7
H-5	-6.01	18	15	19	47
H-6	-6.12	34	11	24	31
H-7	-6.60	19	49	10	22
H-8	-6.71	24	16	54	6
H-9	-6.93	16	31	40	14

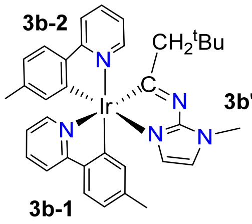

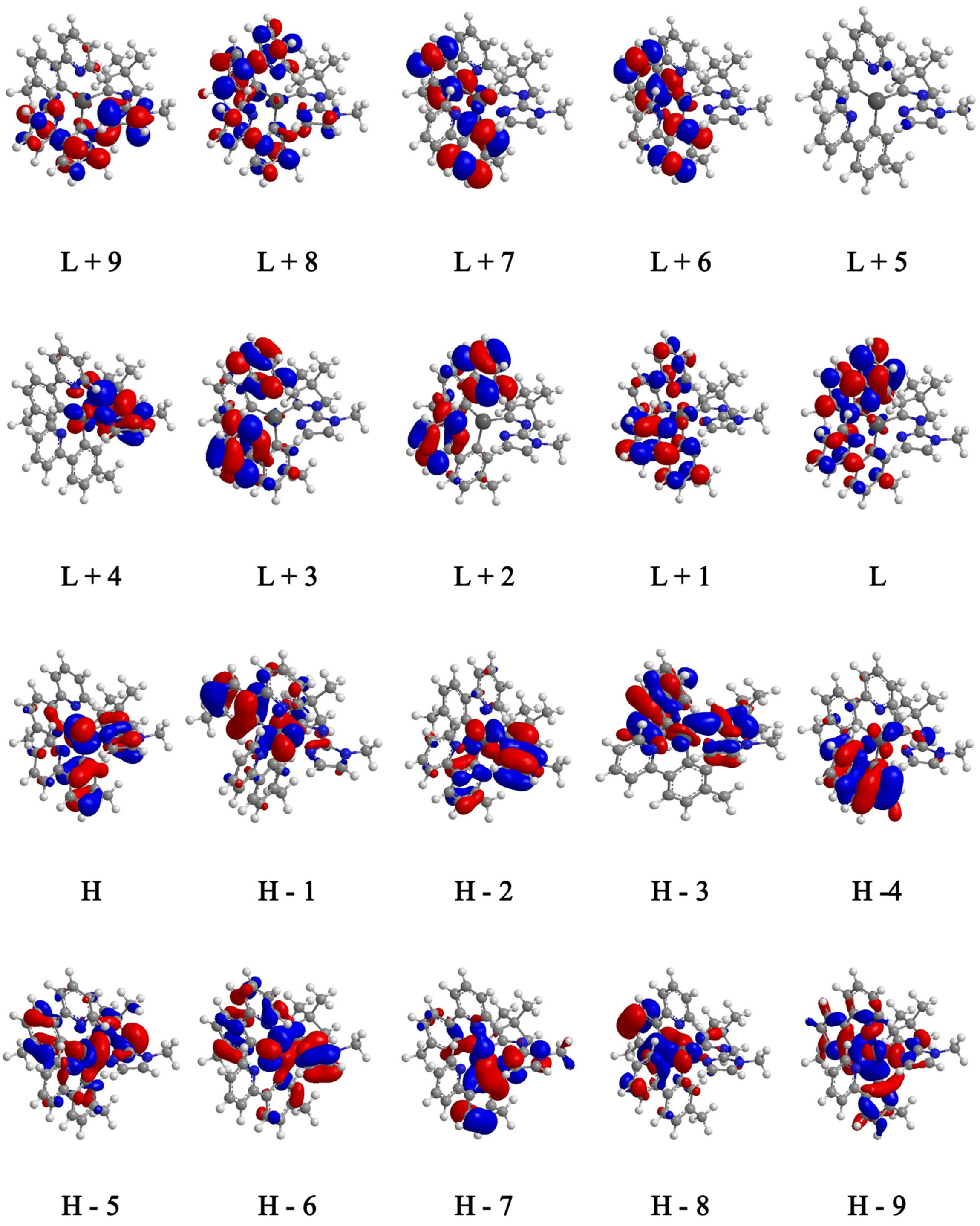

Figure S21. Molecular orbitals of complex **2** (isovalue 0.03 au).

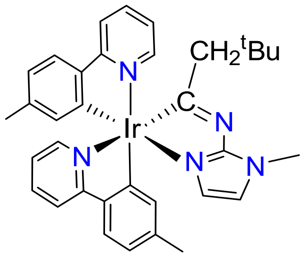

Table S9. Composition (%) of the molecular orbitals of complex 3

MO	eV	Iridium	*p*-tol-py (3b-1)	*p*-tol-py (3b-2)	L (3b')
L+9	0.63	5	50	26	19
L+8	0.58	4	4	21	72
L+7	0.52	6	36	40	18
L+6	0.39	8	4	8	80
L+5	0.16	94	1	1	3
L+4	-0.57	3	55	40	2
L+3	-0.71	6	30	27	37
L+2	-0.85	4	12	31	53
L+1	-1.12	4	76	19	1
LUMO	-1.21	3	21	76	1
HOMO	-5.00	46	32	5	17
H-1	-5.14	41	6	46	7
H-2	-5.46	29	14	5	52
H-3	-5.85	12	53	5	29
H-4	-5.88	7	14	63	17
H-5	-6.08	10	30	4	56
H-6	-6.12	17	20	25	38
H-7	-6.26	24	10	6	59
H-8	-6.73	25	12	57	6
H-9	-6.90	26	45	5	24

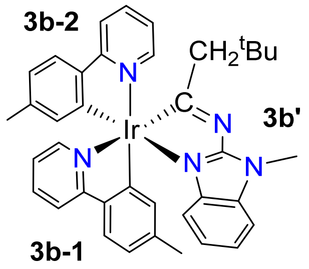

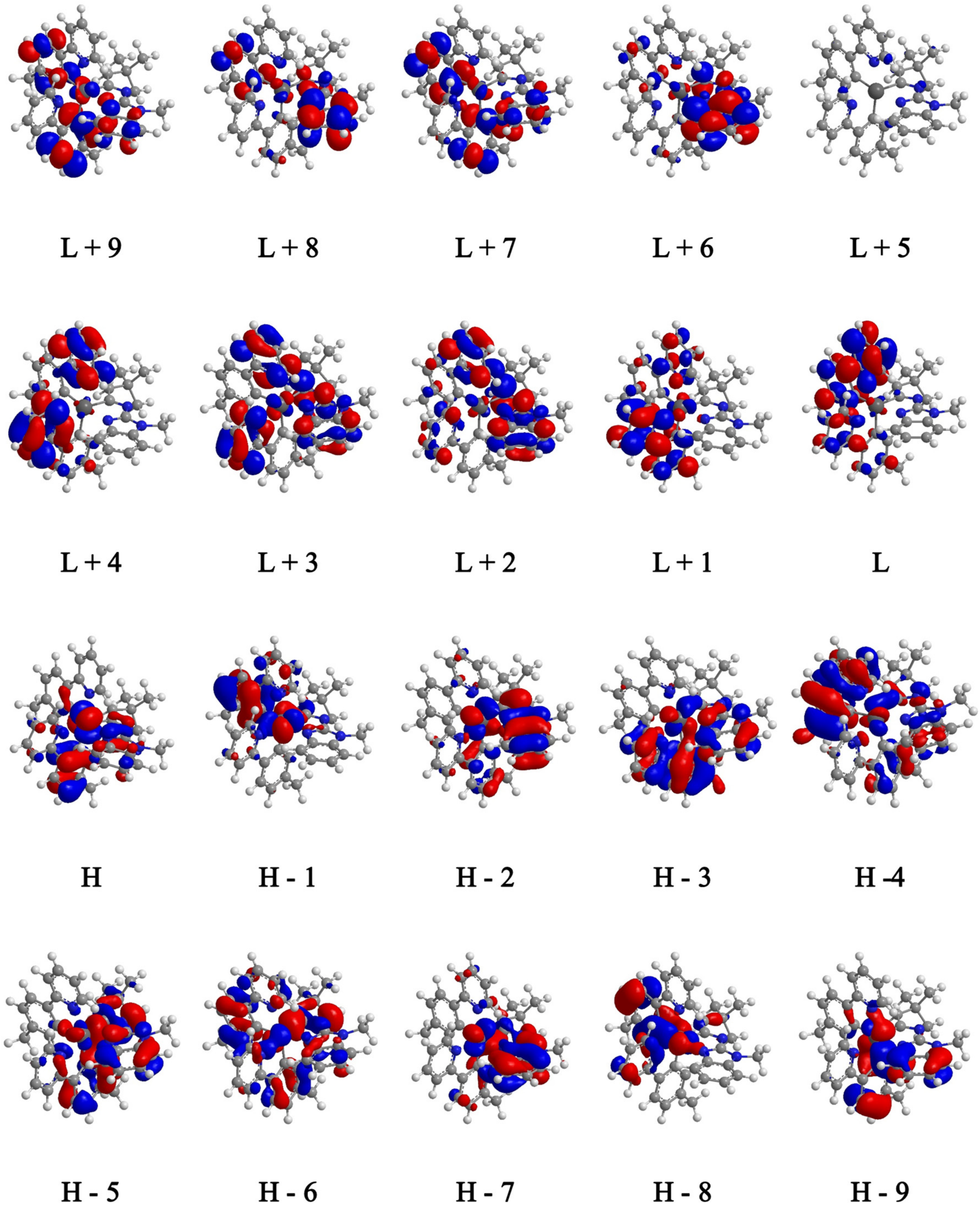

Figure S22. Molecular orbitals of complex **3** (isovalue 0.03 au).

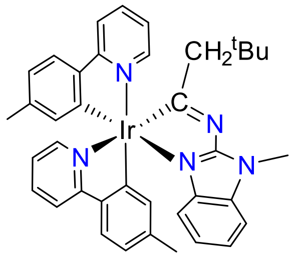

Table S10. Composition (%) of the molecular orbitals of complex 4

MO	eV	Iridium	*p*-tol-py (3b-1)	*p*-tol-py (3b-2)	L (3b')
L+9	1.05	12	1	18	68
L+8	0.99	9	38	42	12
L+7	0.58	6	55	38	1
L+6	0.45	6	38	56	0
L+5	0.16	94	1	1	4
L+4	-0.60	4	54	33	9
L+3	-0.75	11	19	4	66
L+2	-0.84	3	24	63	10
L+1	-1.16	5	72	23	1
LUMO	-1.28	2	25	71	2
HOMO	-5.11	45	35	4	16
H-1	-5.22	40	5	48	6
H-2	-5.56	27	23	7	43
H-3	-5.96	9	45	39	7
H-4	-6.01	10	40	43	7
H-5	-6.26	15	14	8	63
H-6	-6.28	37	10	12	42
H-7	-6.75	28	25	40	8
H-8	-6.88	27	39	22	12
H-9	-7.06	16	32	39	14

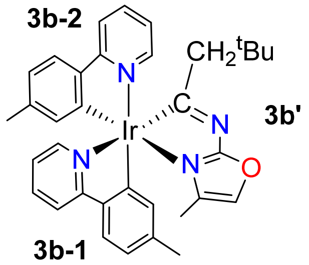

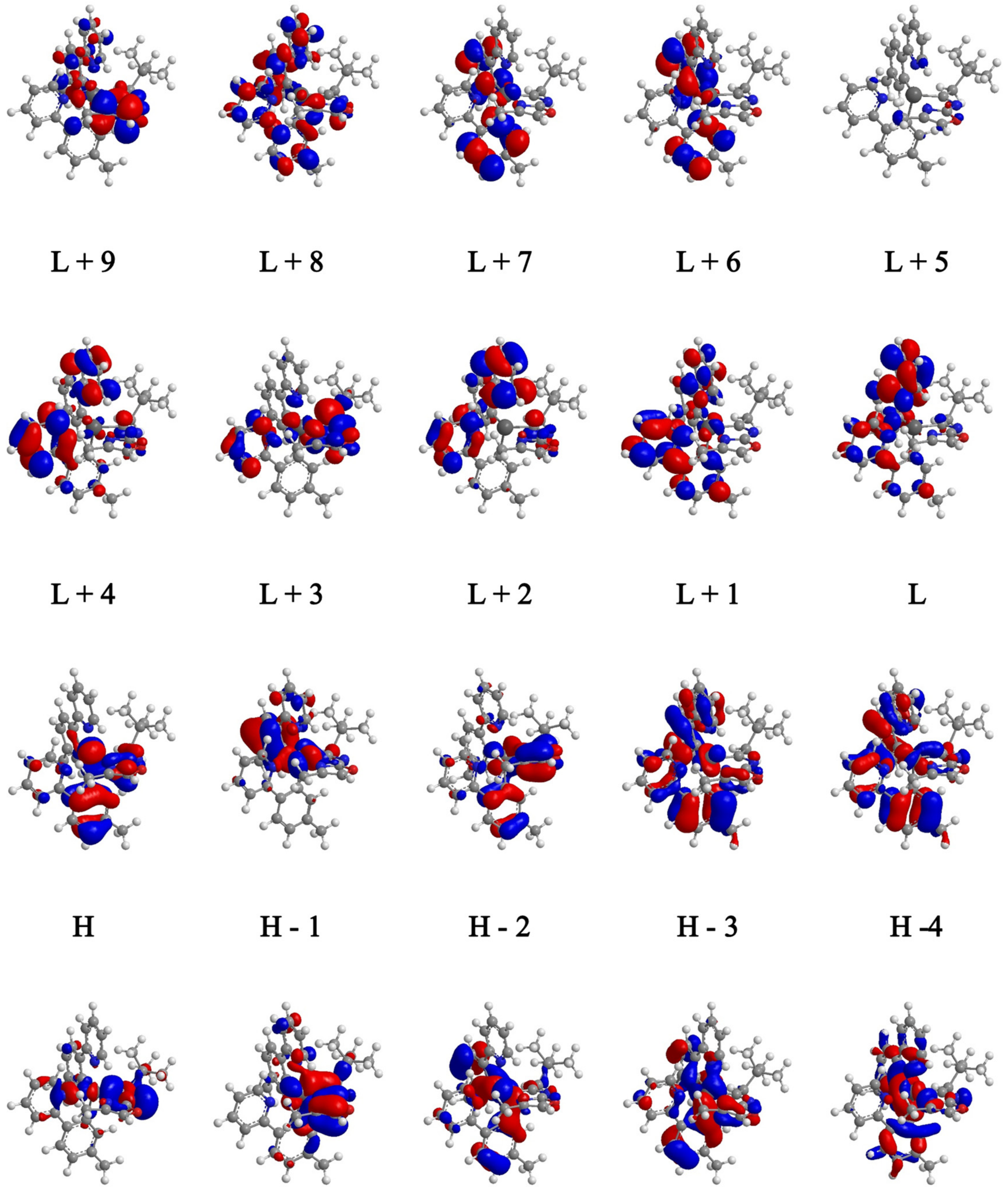

Figure S23. Molecular orbitals of complex **4** (isovalue 0.03 au).

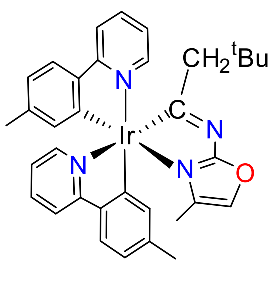

Table S11. Composition (%) of the molecular orbitals of complex 5

MO	eV	Iridium	*p*-tol-py (3b-1)	*p*-tol-py (3b-2)	L (3b')
L+9	0.56	5	58	35	2
L+8	0.45	8	31	56	5
L+7	0.36	4	4	2	90
L+6	0.14	92	1	2	5
L+5	0.04	7	1	1	92
L+4	-0.63	3	59	38	1
L+3	-0.81	4	37	55	4
L+2	-1.10	7	2	9	82
L+1	-1.18	4	74	21	1
LUMO	-1.28	2	22	71	5
HOMO	-5.16	45	35	8	11
H-1	-5.26	39	9	46	6
H-2	-5.70	27	26	8	40
H-3	-5.99	6	4	74	15
H-4	-6.01	16	74	3	6
H-5	-6.30	22	14	15	48
H-6	-6.32	22	14	7	58
H-7	-6.69	9	14	4	73
H-8	-6.83	26	12	52	9
H-9	-7.03	25	36	6	33

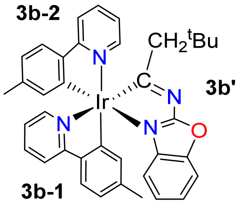

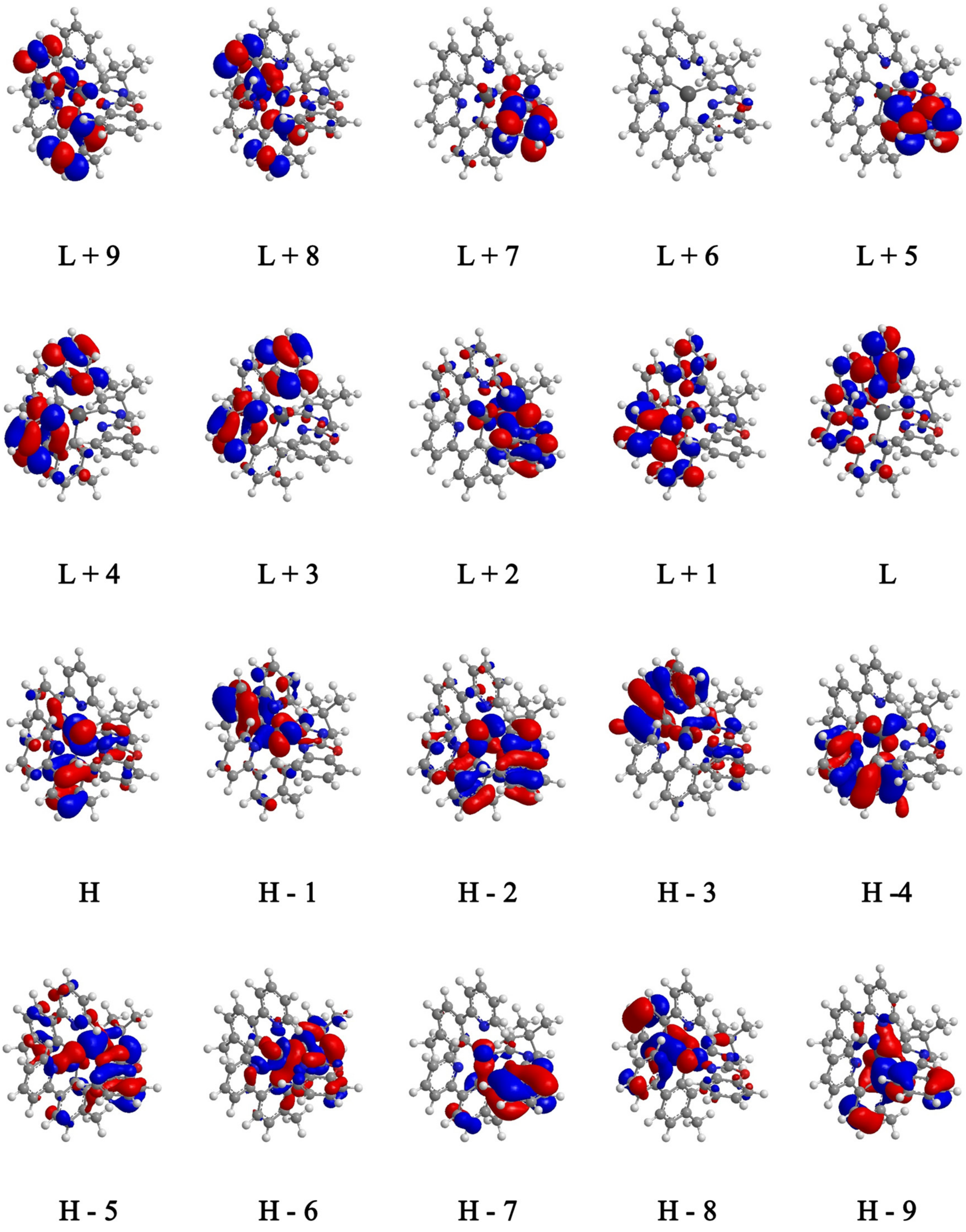

Figure S24. Molecular orbitals of complex **5** (isovalue 0.03 au).

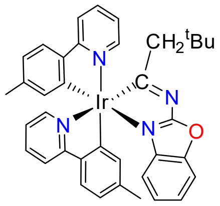

Table S12. Composition (%) of the molecular orbitals of complex 6

MO	eV	Iridium	*p*-tol-py (3b-1)	*p*-tol-py (3b-2)	L (3b')
L+9	0.79	6	4	1	89
L+8	0.66	9	4	10	77
L+7	0.57	6	56	36	3
L+6	0.40	6	32	50	12
L+5	0.15	94	1	1	4
L+4	-0.62	3	66	31	1
L+3	-0.83	4	30	63	3
L+2	-1.05	10	2	10	77
L+1	-1.17	4	79	15	1
LUMO	-1.30	2	16	74	8
HOMO	-5.14	44	38	4	14
H-1	-5.27	40	4	51	5
H-2	-5.52	27	18	5	50
H-3	-5.95	8	61	26	5
H-4	-6.02	8	26	57	9
H-5	-6.20	15	15	6	64
H-6	-6.33	39	7	11	42
H-7	-6.72	24	37	24	14
H-8	-6.83	24	28	36	13
H-9	-7.04	15	29	39	17

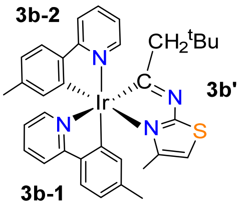

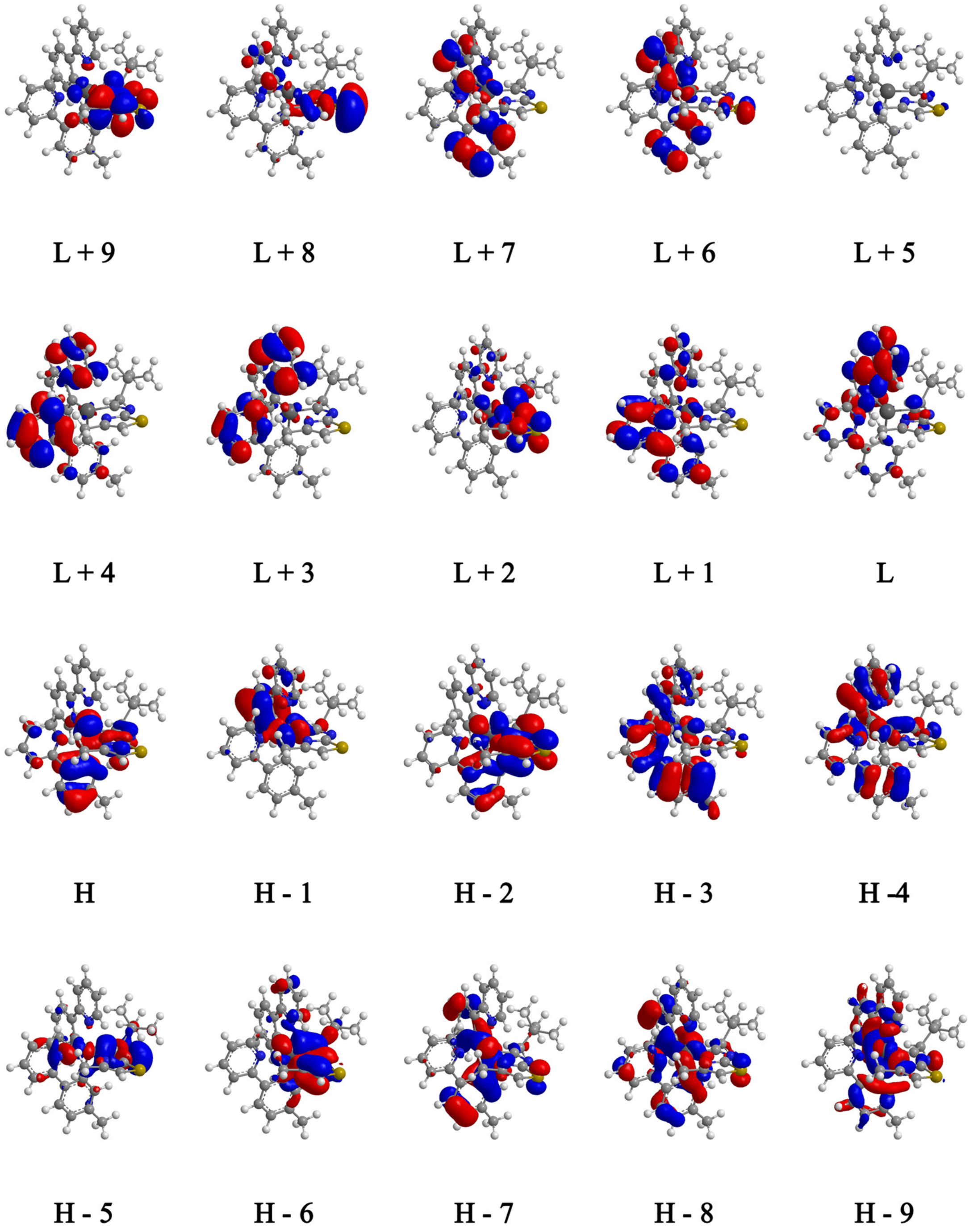

Figure S25. Molecular orbitals of complex **6** (isovalue 0.03 au).

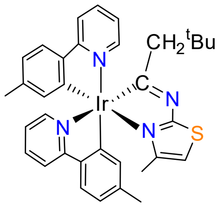

Table S13. Composition (%) of the molecular orbitals of complex 7

MO	eV	Iridium	*p*-tol-py (3b-1)	*p*-tol-py (3b-2)	L (3b')
L+9	0.48	7	20	48	25
L+8	0.34	5	2	2	91
L+7	0.28	7	9	12	72
L+6	0.14	92	2	2	4
L+5	-0.19	3	2	0	95
L+4	-0.64	3	58	39	1
L+3	-0.83	4	36	57	3
L+2	-1.19	4	80	15	1
L+1	-1.24	7	9	40	45
LUMO	-1.31	3	9	44	44
HOMO	-5.17	45	39	6	10
H-1	-5.30	38	8	49	5
H-2	-5.69	31	18	7	44
H-3	-5.98	11	65	15	9
H-4	-6.00	8	13	64	15
H-5	-6.21	15	14	6	66
H-6	-6.29	15	21	10	54
H-7	-6.51	18	7	5	70
H-8	-6.81	26	13	54	7
H-9	-7.01	30	44	4	22

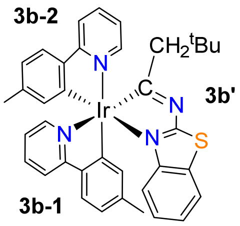

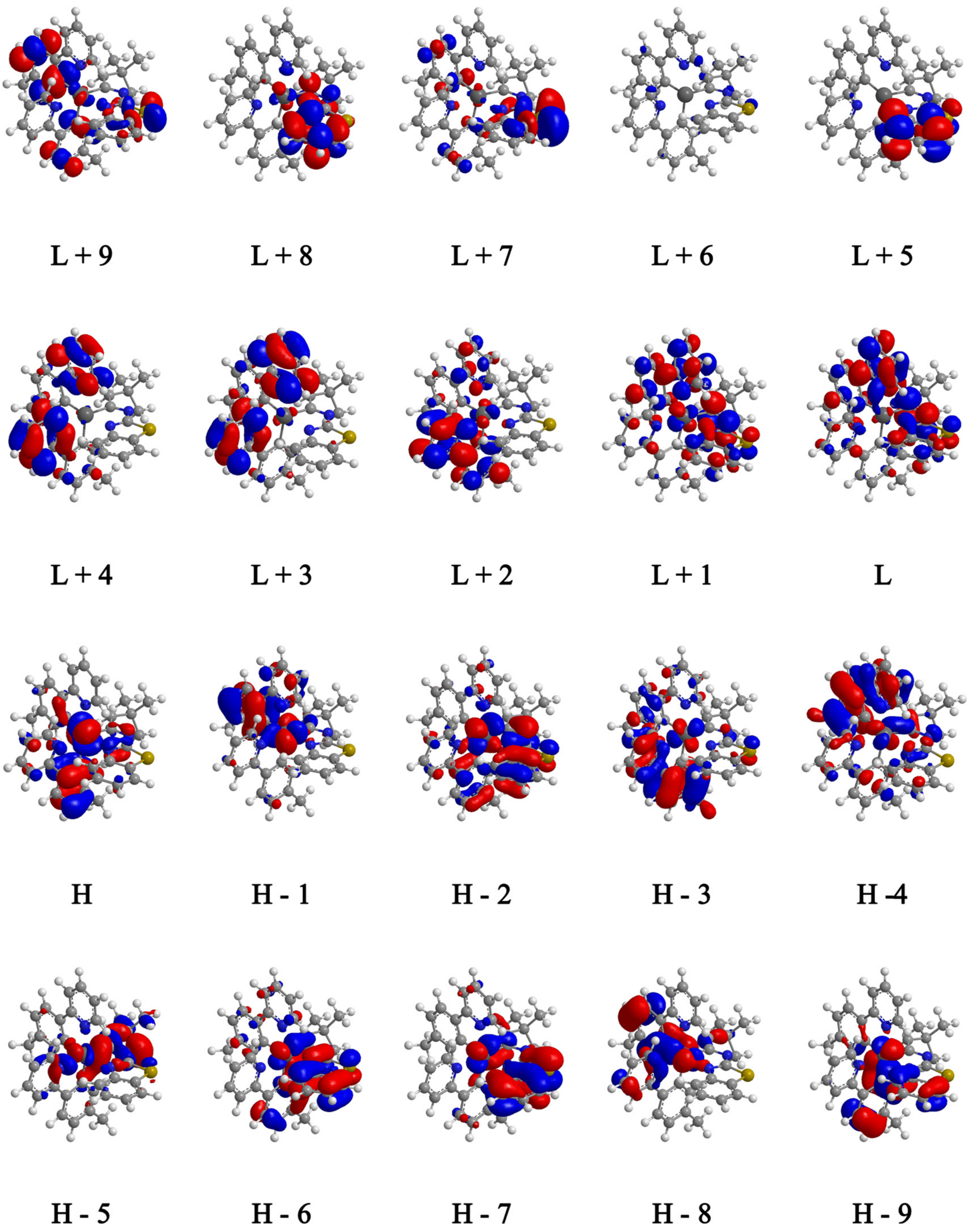

Figure S26. Molecular orbitals of complex **7** (isovalue 0.03 au).

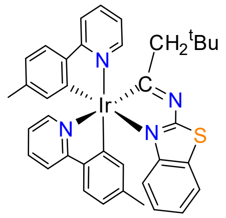

Cyclic Voltammograms

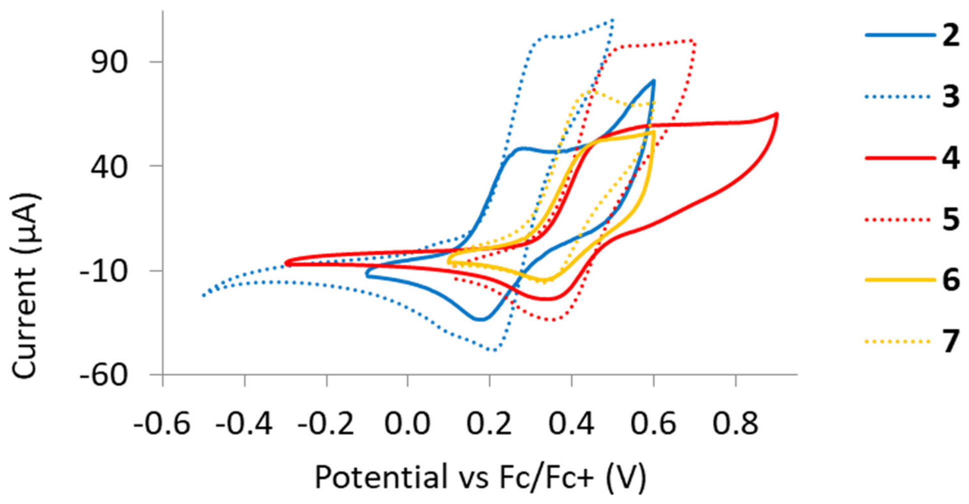

Figure S27. Cyclic voltammograms of complexes **2–7** in CH_2Cl_2 (10^{-3} M) with Bu_4NPF_6 as supporting electrolyte (0.1 M). The potentials are referenced to the ferrocenium/ferrocene (Fc^+/Fc) couple.

Normalized Emission and Excitation Spectra of Complexes 2−7

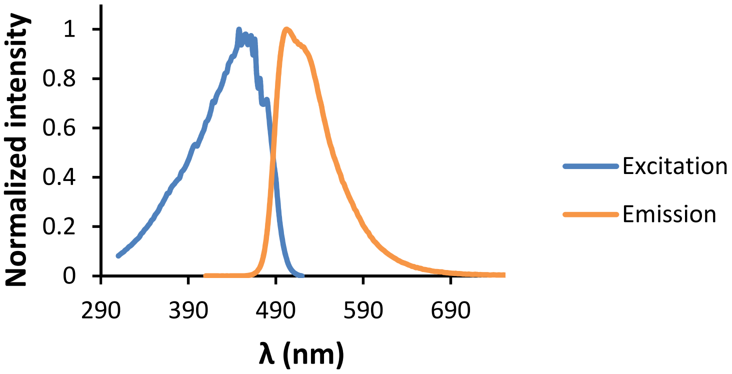

Figure S28. Normalized emission (orange line; λ_{exc} = 390 nm) and excitation (blue line; λ_{em} = 530 nm) spectrum of complex **2** in PMMA film (5 wt %) at 298 K.

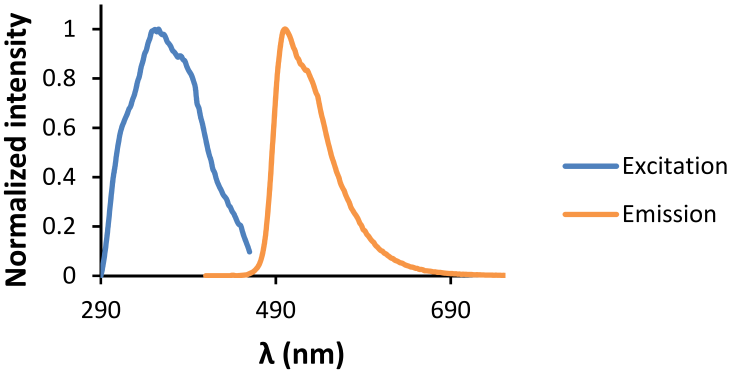

Figure S29. Normalized emission (orange line; λ_{exc} = 390 nm) and excitation (blue line; λ_{em} = 500 nm) spectrum for a 1 x 10^{-5} M solution of complex **2** in 2-MeTHF at 298 K.

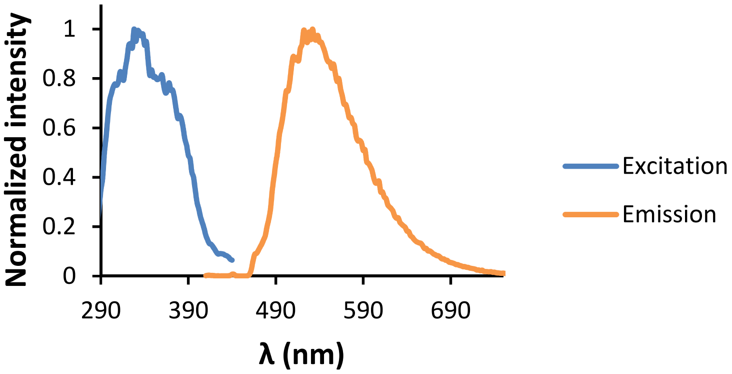

Figure S30. Normalized emission (orange line; λ_{exc} = 390 nm) and excitation (blue line; λ_{em} = 520 nm) spectrum for a 1 x 10^{-5} M solution of complex **2** in 2-MeTHF at 77 K.

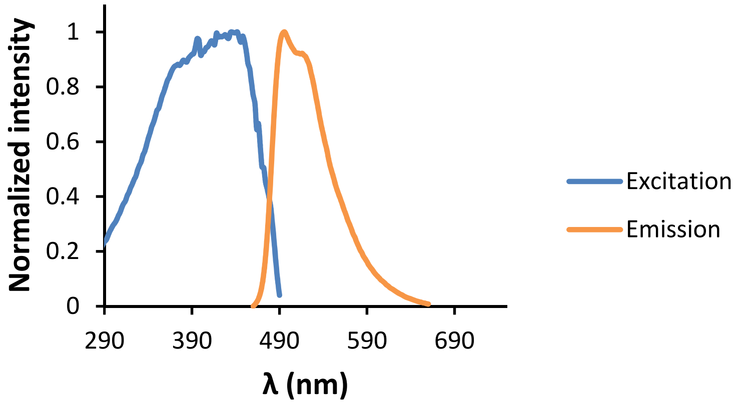

Figure S31. Normalized emission (orange line; λ_{exc} = 380 nm) and excitation (blue line; λ_{em} = 540 nm) spectrum of complex **3** in PMMA film (5 wt %) at 298 K.

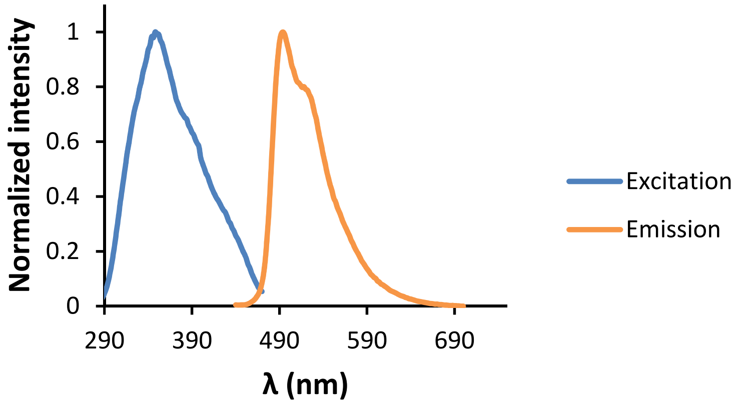

Figure S32. Normalized emission (orange line; λ_{exc} = 370 nm) and excitation (blue line; λ_{em} = 495 nm) spectrum for a 1 x 10^{-5} M solution of complex **3** in 2-MeTHF at 298 K.

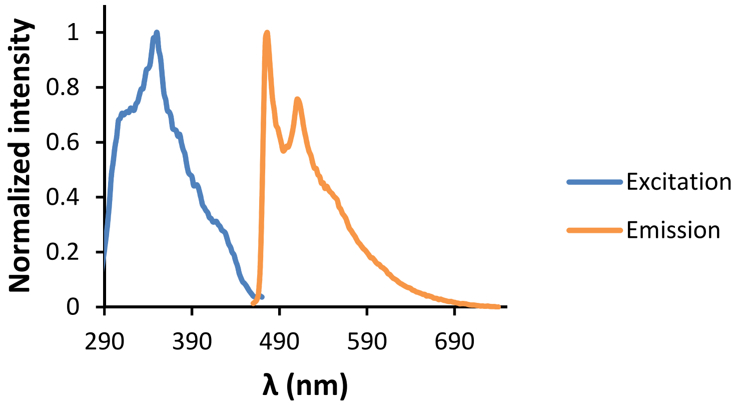

Figure S33. Normalized emission (orange line; λ_{exc} = 380 nm) and excitation (blue line; λ_{em} = 510 nm) spectrum for a 1 x 10^{-5} M solution of complex **3** in 2-MeTHF at 77 K.

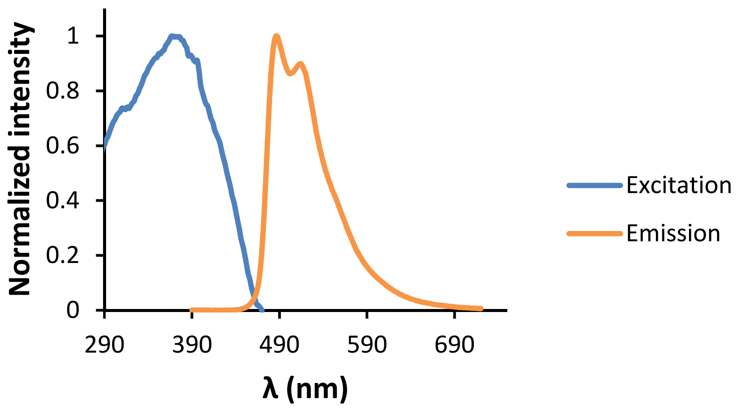

Figure S34. Normalized emission (orange line; λ_{exc} = 370 nm) and excitation (blue line; λ_{em} = 500 nm) spectrum of complex **4** in PMMA film (5 wt %) at 298 K.

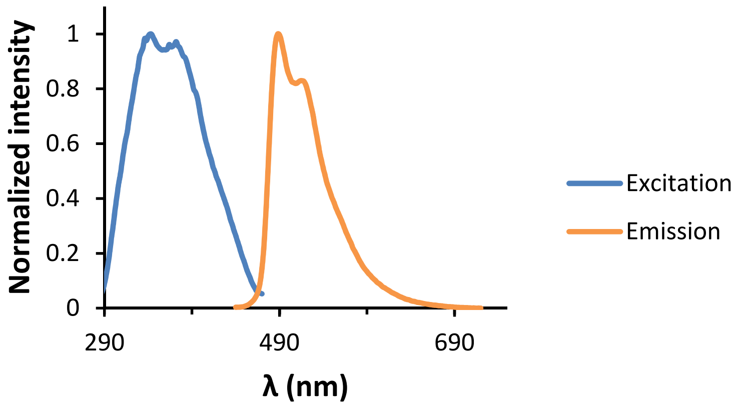

Figure S35. Normalized emission (orange line; λ_{exc} = 370 nm) and excitation (blue line; λ_{em} = 510 nm) spectrum for a 1 x 10^{-5} M solution of complex **4** in 2-MeTHF at 298 K.

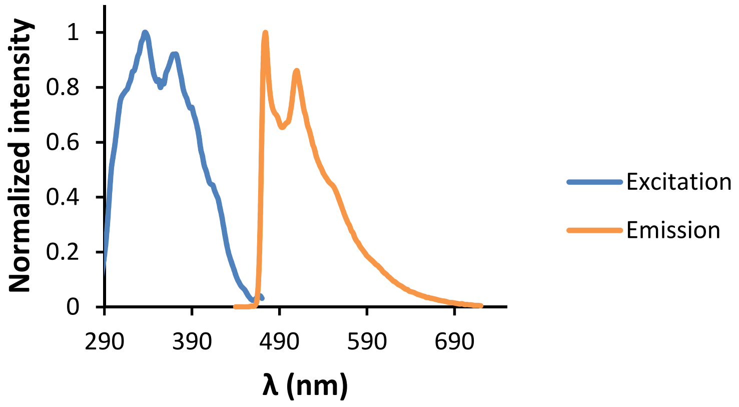

Figure S36. Normalized emission (orange line; λ_{exc} = 370 nm) and excitation (blue line; λ_{em} = 510 nm) spectrum for a 1 x 10^{-5} M solution of complex **4** in 2-MeTHF at 77 K.

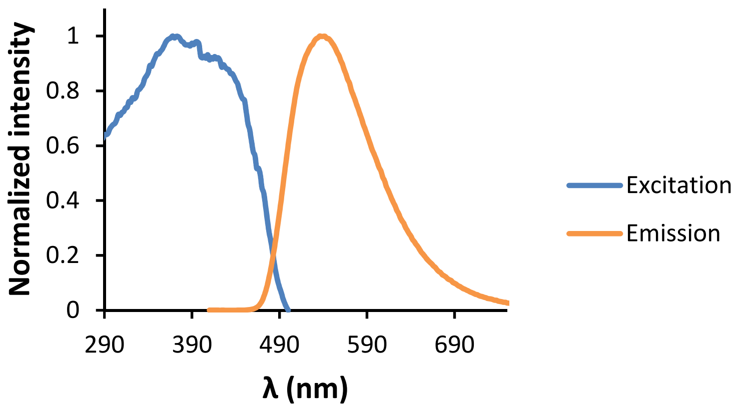

Figure S37. Normalized emission (orange line; λ_{exc} = 390 nm) and excitation (blue line; λ_{em} = 530 nm) spectrum of complex **5** in PMMA film (5 wt %) at 298 K.

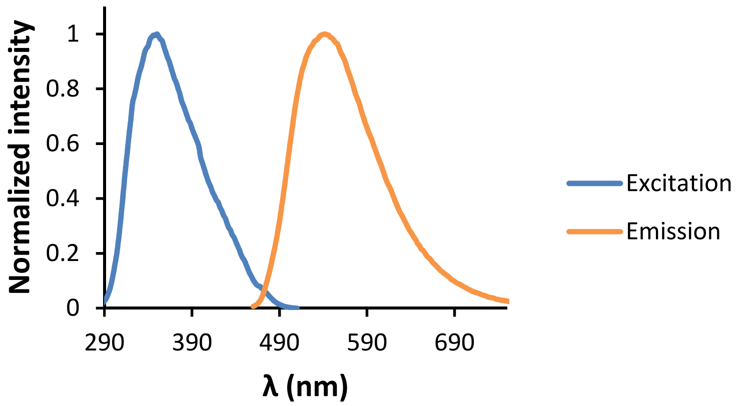

Figure S38. Normalized emission (orange line; λ_{exc} = 390 nm) and excitation (blue line; λ_{em} = 530 nm) spectrum for a 1 x 10^{-5} M solution of complex **5** in 2-MeTHF at 298 K.

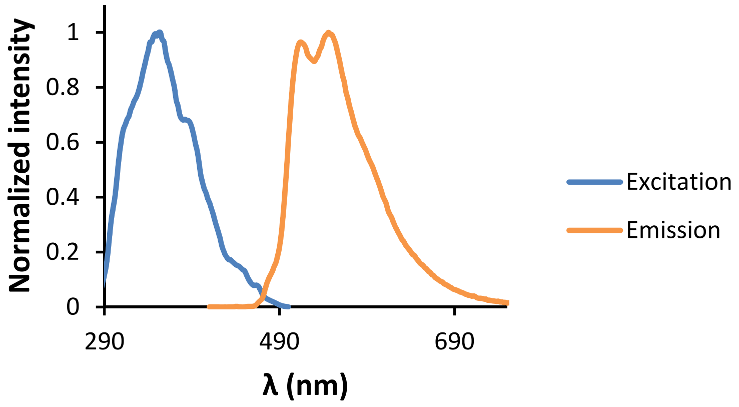

Figure S39. Normalized emission (orange line; λ_{exc} = 390 nm) and excitation (blue line; λ_{em} = 530 nm) spectrum for a 1 x 10^{-5} M solution of complex **5** in 2-MeTHF at 77 K.

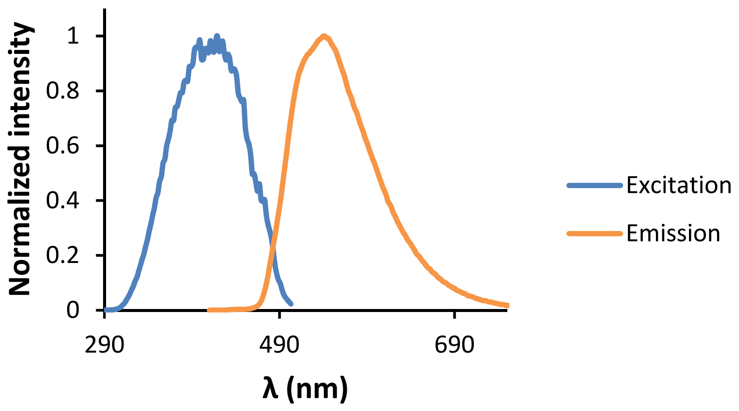

Figure S40. Normalized emission (orange line; λ_{exc} = 390 nm) and excitation (blue line; λ_{em} = 540 nm) spectrum of complex **6** in PMMA film (5 wt %) at 298 K.

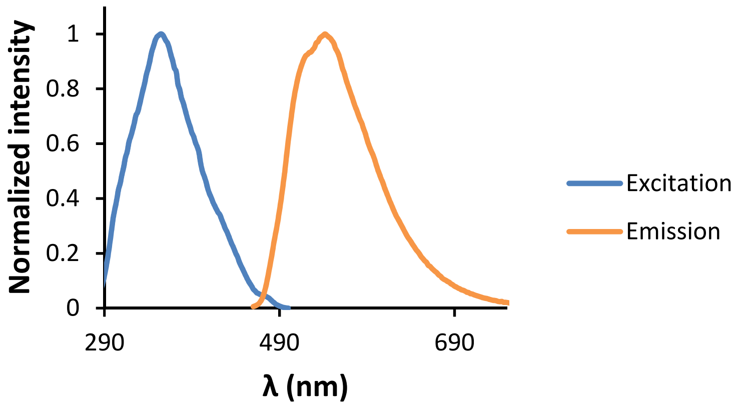

Figure S41. Normalized emission (orange line; λ_{exc} = 390 nm) and excitation (blue line; λ_{em} = 540 nm) spectrum for a 1 x 10^{-5} M solution of complex **6** in 2-MeTHF at 298 K.

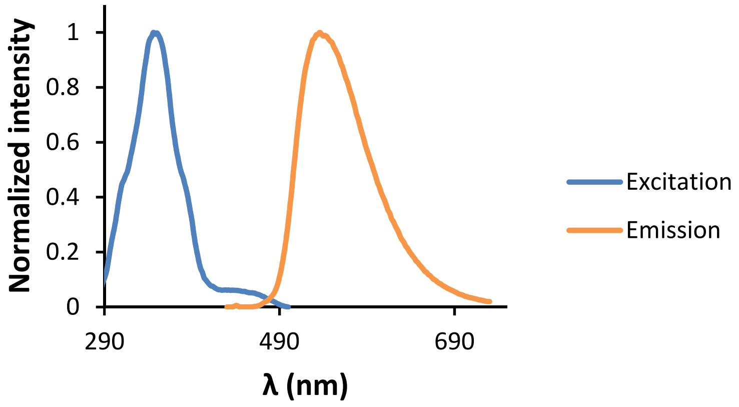

Figure S42. Normalized emission (orange line; λ_{exc} = 390 nm) and excitation (blue line; λ_{em} = 540 nm) spectrum for a 1 x 10^{-5} M solution of complex **6** in 2-MeTHF at 77 K.

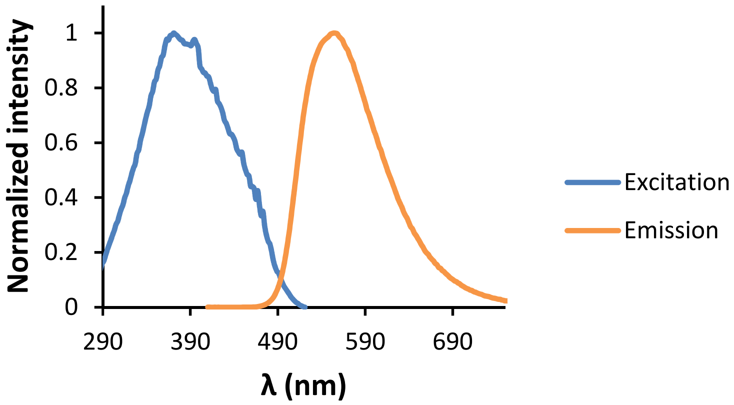

Figure S43. Normalized emission (orange line; λ_{exc} = 390 nm) and excitation (blue line; λ_{em} = 560 nm) spectrum of complex **7** in PMMA film (5 wt %) at 298 K.

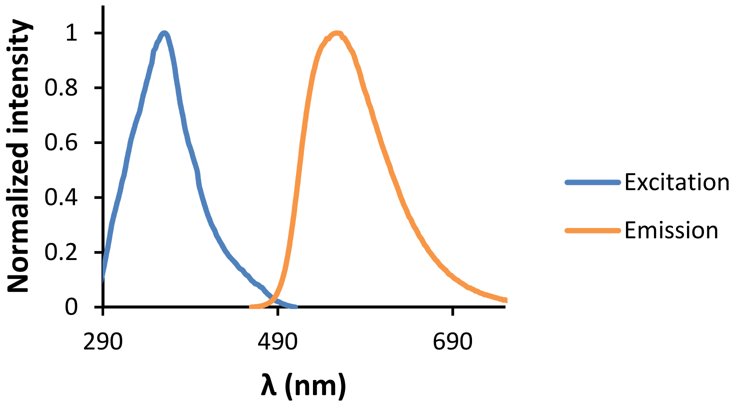

Figure S44. Normalized emission (orange line; λ_{exc} = 390 nm) and excitation (blue line; λ_{em} = 530 nm) spectrum for a 1 x 10^{-5} M solution of complex **7** in 2-MeTHF at 298 K.

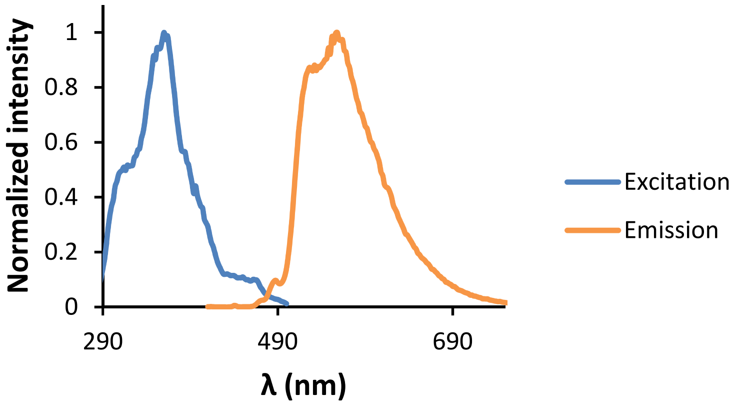

Figure S45. Normalized emission (orange line; λ_{exc} = 390 nm) and excitation (blue line; λ_{em} = 530 nm) spectrum for a 1 x 10^{-5} M solution of complex **7** in 2-MeTHF at 77 K.

Time-resolved Photoluminescence Decay of Complexes 2−7

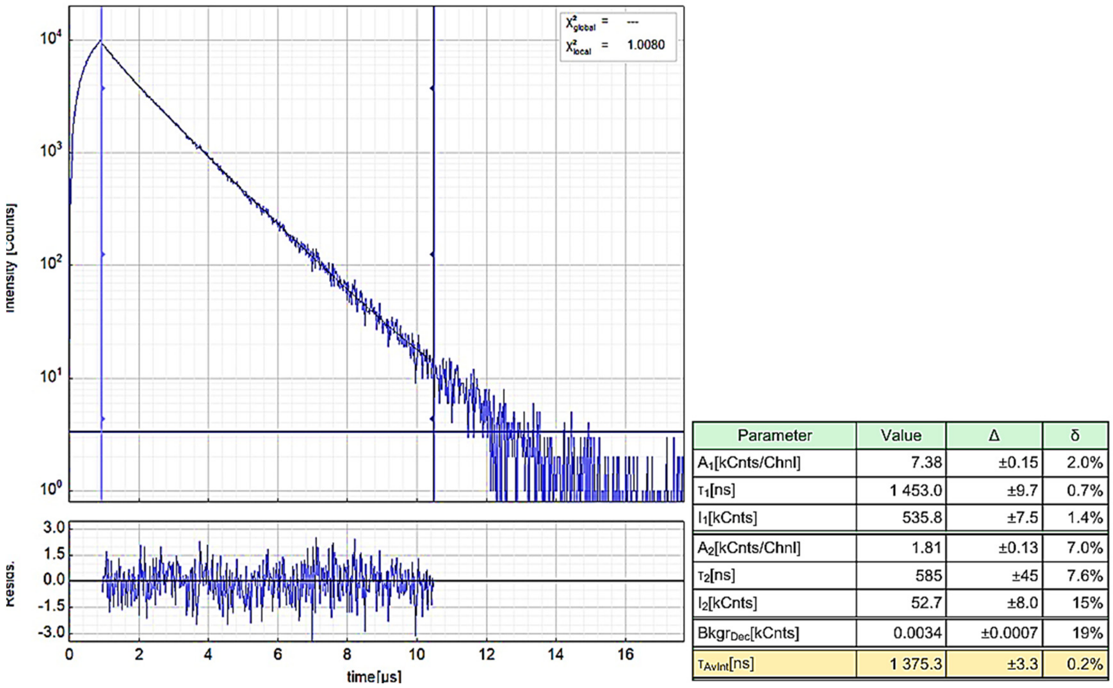

Parameter	Value	Δ	δ
A_1[kCnts/Chnl]	7.38	±0.15	2.0%
τ_1[ns]	1 453.0	±9.7	0.7%
I_1[kCnts]	535.8	±7.5	1.4%
A_2[kCnts/Chnl]	1.81	±0.13	7.0%
τ_2[ns]	585	±45	7.6%
I_2[kCnts]	52.7	±8.0	15%
$Bkgr_{Dec}$[kCnts]	0.0034	±0.0007	19%
τ_{AvInt}[ns]	1 375.3	±3.3	0.2%

Figure S46. Left: Raw (experimental) time-resolved photoluminescence decay of **2** in PMMA film (5 wt%) at 298 K (λ_{exc} = 450 nm, λ_{em} = 505 nm). Right: Fitting parameters and confidence limits.

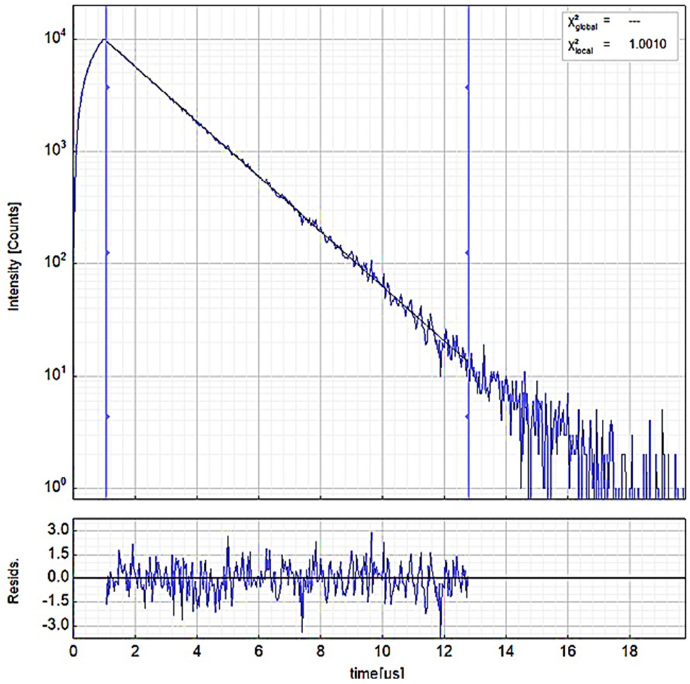

Parameter	Value	Δ	δ
A_1[kCnts/Chnl]	9.568	±0.029	0.3%
τ_1[ns]	1 773.8	±4.9	0.3%
I_1[kCnts]	424.28	±0.62	0.1%
$Bkgr_{Dec}$[kCnts]	0.0003	±0.0010	436%
τ_{AvInt}[ns]	1 773.8	±4.9	0.3%

Figure S47. Left: Raw (experimental) time-resolved photoluminescence decay of **2** in 2-MeTHF at 298 K (λ_{exc} = 375 nm, λ_{em} = 505 nm). Right: Fitting parameters and confidence limits.

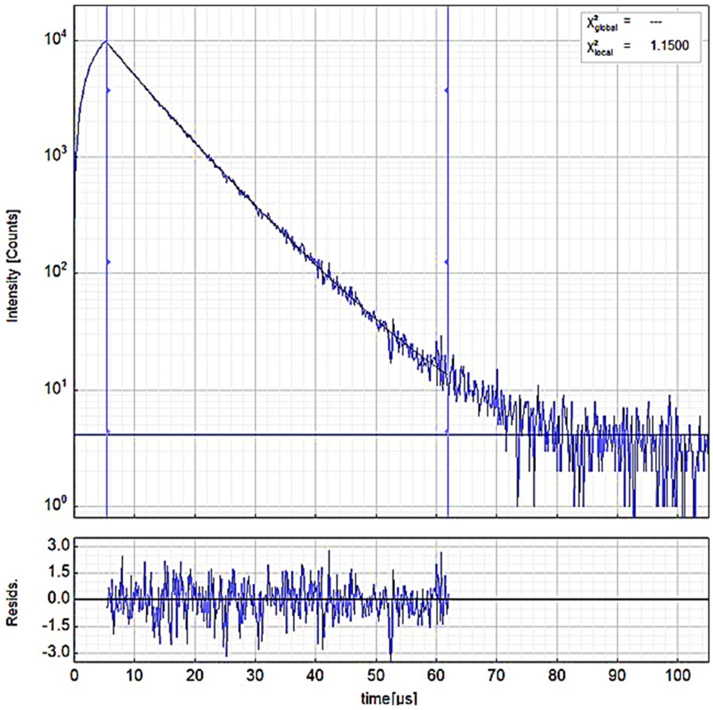

Parameter	Value	Δ	δ
A_1[kCnts/Chnl]	4.8	±1.9	40%
τ_1[ns]	9 040	±730	8.1%
I_1[kCnts]	268	±89	33%
A_2[kCnts/Chnl]	4.9	±1.9	38%
τ_2[ns]	5 700	±780	14%
I_2[kCnts]	172	±91	52%
$Bkgr_{Dec}$[kCnts]	0.0041	±0.0021	50%
τ_{AvInt}[ns]	7 731	±47	0.6%

Figure S48. Left: Raw (experimental) time-resolved photoluminescence decay of **2** in 2-MeTHF at 77 K (λ_{exc} = 375 nm, λ_{em} = 530 nm). Right: Fitting parameters and confidence limits.

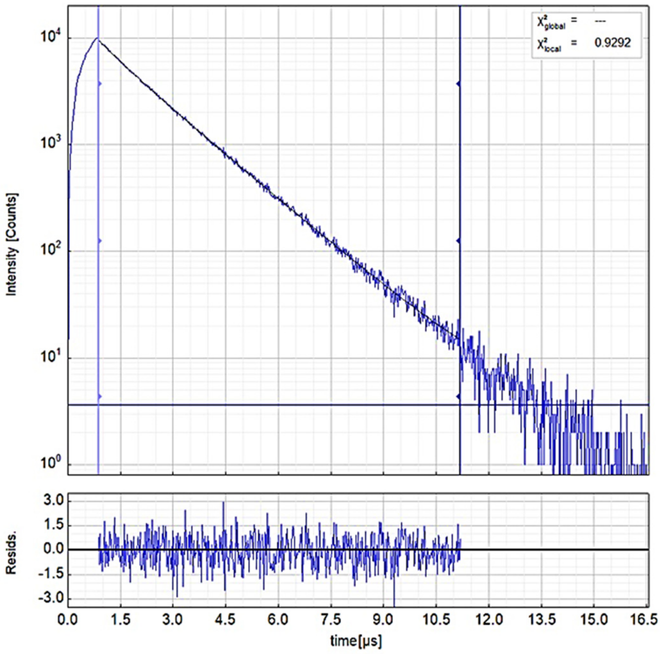

Parameter	Value	Δ	δ
A_1[kCnts/Chnl]	8.10	±0.17	2.0%
τ_1[ns]	1 568	±11	0.7%
I_1[kCnts]	634.1	±7.9	1.2%
A_2[kCnts/Chnl]	1.42	±0.14	9.8%
τ_2[ns]	667	±61	9.1%
I_2[kCnts]	47.3	±9.2	19%
$Bkgr_{Dec}$[kCnts]	0.0036	±0.0007	18%
τ_{AvInt}[ns]	1 504.9	±1.7	0.1%

Figure S49. Left: Raw (experimental) time-resolved photoluminescence decay of **3** in PMMA film (5 wt%) at 298 K (λ_{exc} = 375 nm, λ_{em} = 495 nm). Right: Fitting parameters and confidence limits.

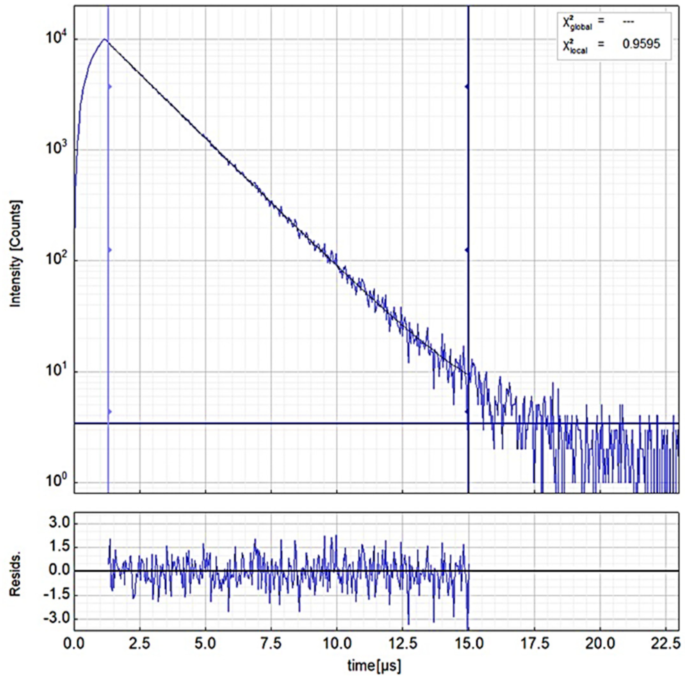

Parameter	Value	Δ	δ
A_1[kCnts/Chnl]	9.113	±0.016	0.2%
τ_1[ns]	1 863.6	±3.6	0.2%
I_1[kCnts]	424.54	±0.72	0.2%
$Bkgr_{Dec}$[kCnts]	0.0034	±0.0004	12%
τ_{AvInt}[ns]	1 863.6	±3.6	0.2%

Figure S50. Left: Raw (experimental) time-resolved photoluminescence decay of **3** in 2-MeTHF at 298 K (λ_{exc} = 375 nm, λ_{em} = 495 nm). Right: Fitting parameters and confidence limits.

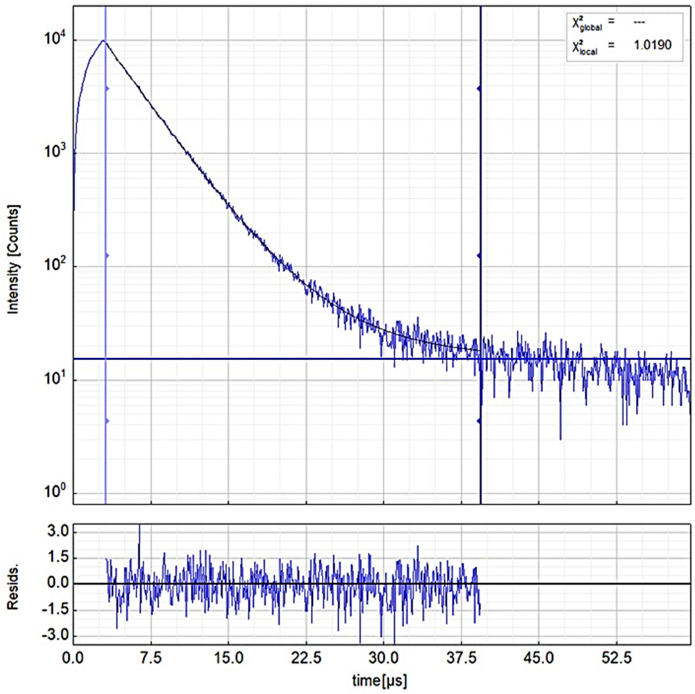

Parameter	Value	Δ	δ
A_1[kCnts/Chnl]	0.35	±0.24	68%
τ_1[ns]	7 500	±1 700	23%
I_1[kCnts]	32	±14	43%
A_2[kCnts/Chnl]	8.90	±0.24	2.7%
τ_2[ns]	3 364	±63	1.9%
I_2[kCnts]	374	±15	4.0%
$Bkgr_{Dec}$[kCnts]	0.0154	±0.0024	15%
τ_{AvInt}[ns]	3 683	±69	1.9%

Figure S51. Left: Raw (experimental) time-resolved photoluminescence decay of **3** in 2-MeTHF at 77 K (λ_{exc} = 375 nm, λ_{em} = 484 nm). Right: Fitting parameters and confidence limits.

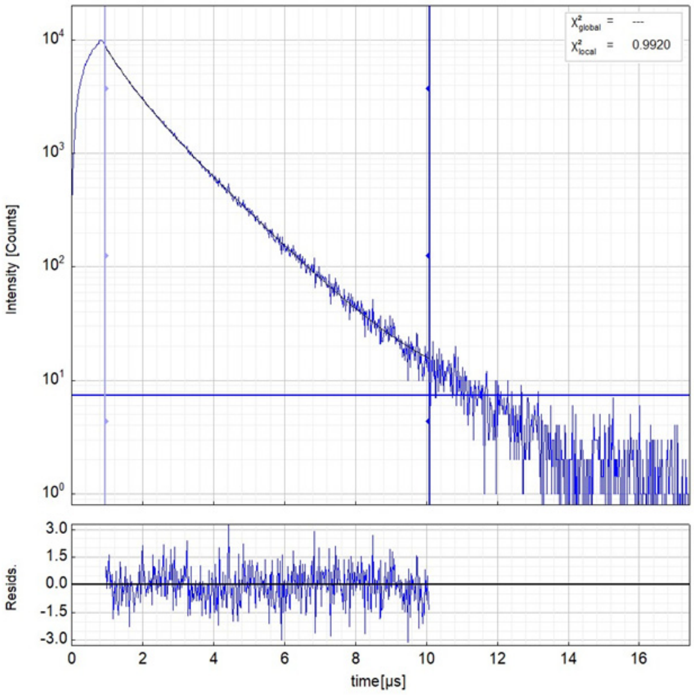

Parameter	Value	Δ	δ
A_1[kCnts/Chnl]	5.17	±0.19	3.6%
$τ_1$[ns]	1 416	±20	1.4%
I_1[kCnts]	365.7	±8.1	2.2%
A_2[kCnts/Chnl]	3.43	±0.17	4.8%
$τ_2$[ns]	554	±21	3.7%
I_2[kCnts]	94.7	±8.1	8.5%
$Bkgr_{Dec}$[kCnts]	0.0074	±0.0008	11%
$τ_{AvInt}$[ns]	1 237.9	±3.8	0.3%

Figure S52. Left: Raw (experimental) time-resolved photoluminescence decay of **4** in PMMA film (5 wt%) at 298 K ($λ_{exc}$ = 375 nm, $λ_{em}$ = 495 nm). Right: Fitting parameters and confidence limits.

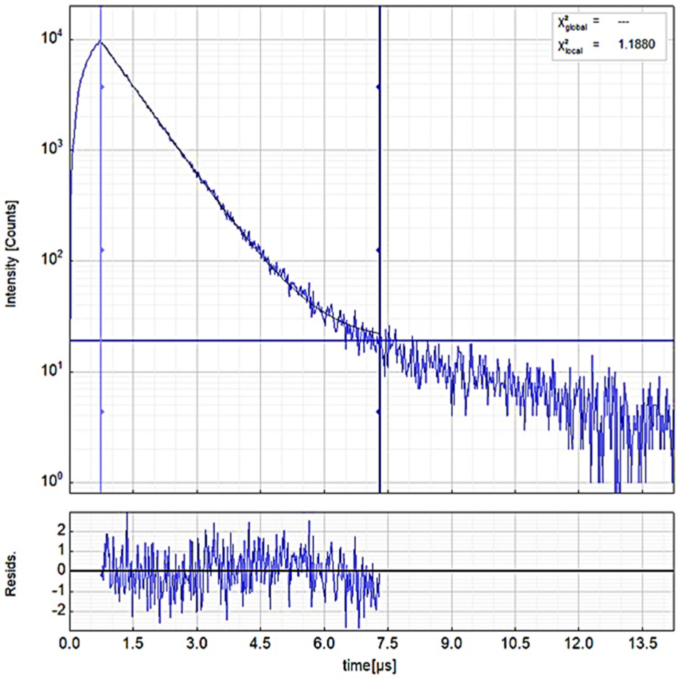

Parameter	Value	Δ	δ
A_1[kCnts/Chnl]	9.550	±0.011	0.1%
$τ_1$[ns]	815.20	±0.50	0.1%
I_1[kCnts]	389.25	±0.54	0.1%
$Bkgr_{Dec}$[kCnts]	0.0192	±0.0004	1.9%
$τ_{AvInt}$[ns]	815.20	±0.50	0.1%

Figure S53. Left: Raw (experimental) time-resolved photoluminescence decay of **4** in 2-MeTHF at 298 K ($λ_{exc}$ = 375 nm, $λ_{em}$ = 495 nm). Right: Fitting parameters and confidence limits.

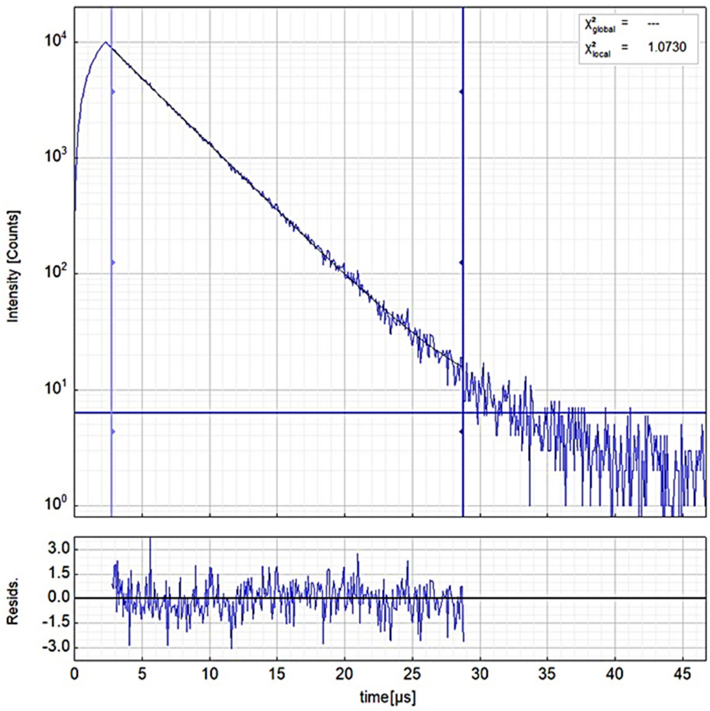

Parameter	Value	Δ	δ
A_1[kCnts/Chnl]	8.695	±0.024	0.3%
τ_1[ns]	3 793.7	±4.8	0.1%
I_1[kCnts]	412.4	±1.4	0.3%
$Bkgr_{Dec}$[kCnts]	0.0063	±0.0005	7.5%
τ_{AvInt}[ns]	3 793.7	±4.8	0.1%

Figure S54. Left: Raw (experimental) time-resolved photoluminescence decay of **4** in 2-MeTHF at 77 K (λ_{exc} = 375 nm, λ_{em} = 514 nm). Right: Fitting parameters and confidence limits.

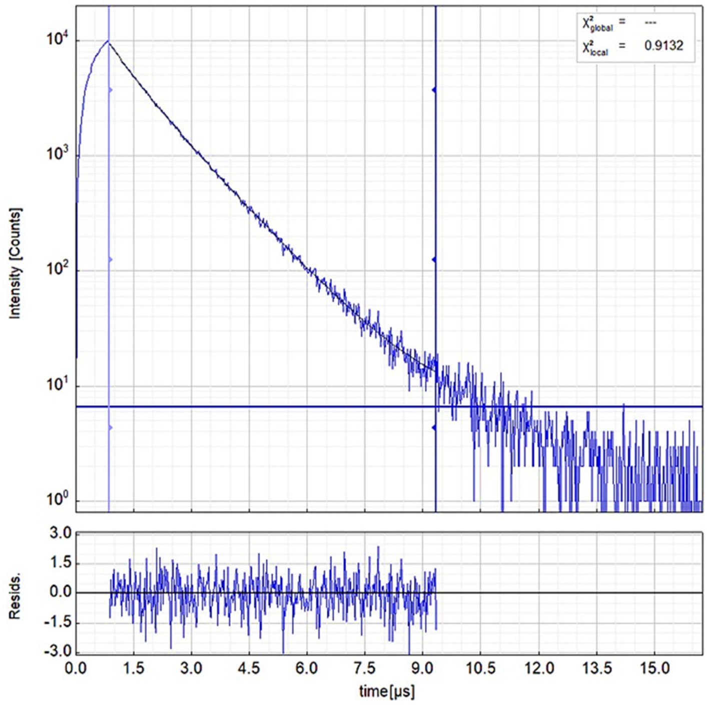

Parameter	Value	Δ	δ
A_1[kCnts/Chnl]	6.15	±0.61	9.8%
τ_1[ns]	1 240	±32	2.5%
I_1[kCnts]	381	±27	7.0%
A_2[kCnts/Chnl]	3.15	±0.60	19%
τ_2[ns]	620	±67	11%
I_2[kCnts]	98	±27	27%
$Bkgr_{Dec}$[kCnts]	0.0067	±0.0012	17%
τ_{AvInt}[ns]	1 113.0	±2.3	0.2%

Figure S55. Left: Raw (experimental) time-resolved photoluminescence decay of **5** in PMMA film (5 wt%) at 298 K (λ_{exc} = 375 nm, λ_{em} = 530 nm). Right: Fitting parameters and confidence limits.

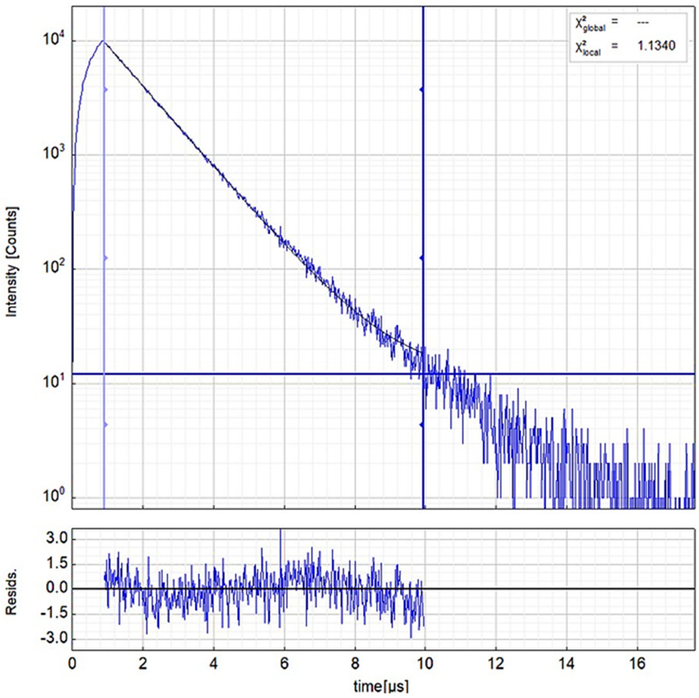

Parameter	Value	Δ	δ
A_1[kCnts/Chnl]	9.612	±0.018	0.2%
τ_1[ns]	1 233.5	±1.7	0.1%
I_1[kCnts]	592.76	±0.68	0.1%
$Bkgr_{Dec}$[kCnts]	0.0121	±0.0009	7.4%
τ_{AvInt}[ns]	1 233.5	±1.7	0.1%

Figure S56. Left: Raw (experimental) time-resolved photoluminescence decay of **5** in 2-MeTHF at 298 K (λ_{exc} = 375 nm, λ_{em} = 535 nm). Right: Fitting parameters and confidence limits.

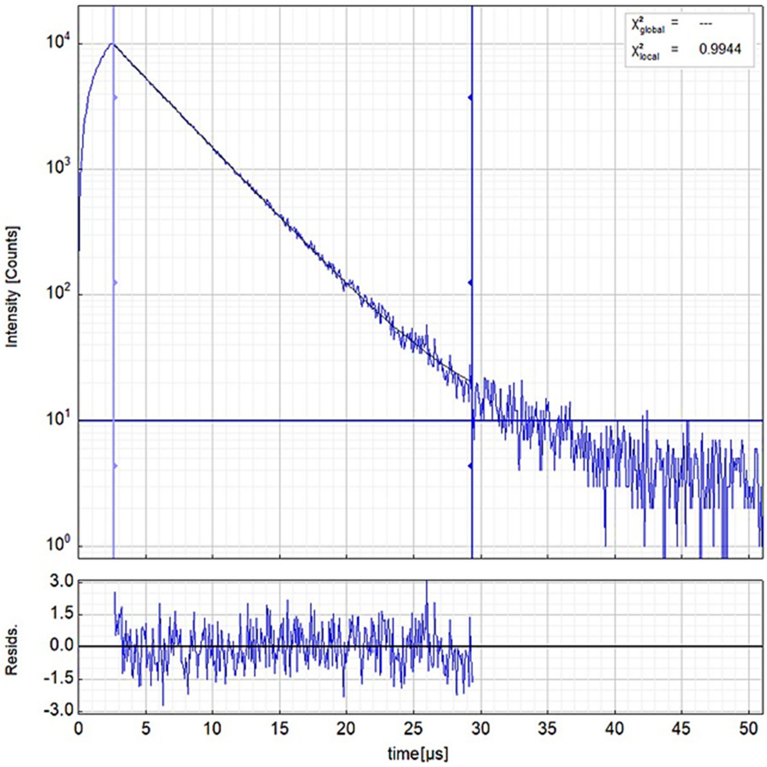

Parameter	Value	Δ	δ
A_1[kCnts/Chnl]	9.556	±0.022	0.2%
τ_1[ns]	3 899.3	±8.5	0.2%
I_1[kCnts]	465.75	±0.59	0.1%
$Bkgr_{Dec}$[kCnts]	0.0100	±0.0007	6.3%
τ_{AvInt}[ns]	3 899.3	±8.5	0.2%

Figure S57. Left: Raw (experimental) time-resolved photoluminescence decay of **5** in 2-MeTHF at 77 K (λ_{exc} = 375 nm, λ_{em} = 515 nm). Right: Fitting parameters and confidence limits.

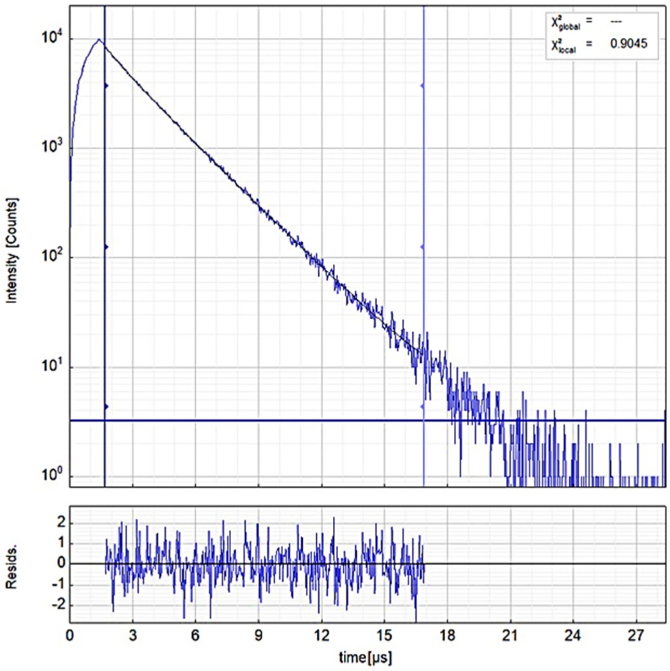

Parameter	Value	Δ	δ
A_1[kCnts/Chnl]	7.08	±0.20	2.7%
$τ_1$[ns]	2 294	±24	1.0%
I_1[kCnts]	405.8	±6.8	1.7%
A_2[kCnts/Chnl]	1.24	±0.17	14%
$τ_2$[ns]	940	±110	12%
I_2[kCnts]	28.8	±7.4	25%
$Bkgr_{Dec}$[kCnts]	0.0033	±0.0013	38%
$τ_{AvInt}$[ns]	2 203.4	±4.8	0.2%

Figure S58. Raw (experimental) time-resolved photoluminescence decay of **6** in PMMA film (5 wt%) at 298 K ($λ_{exc}$ = 375 nm, $λ_{em}$ = 530 nm), fitting parameters and confidence limits.

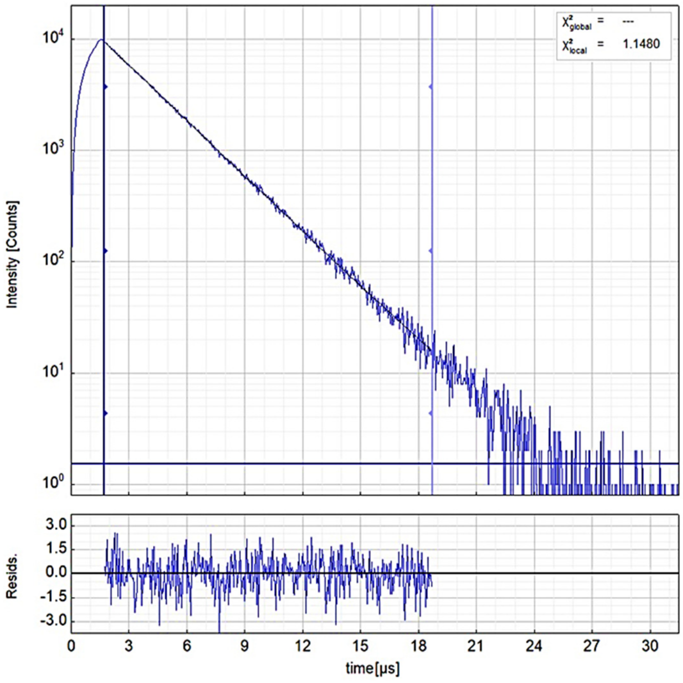

Parameter	Value	Δ	δ
A_1[kCnts/Chnl]	9.327	±0.017	0.2%
$τ_1$[ns]	2 616.0	±3.7	0.1%
I_1[kCnts]	609.95	±0.36	0.1%
$Bkgr_{Dec}$[kCnts]	0.0016	±0.0005	30%
$τ_{AvInt}$[ns]	2 616.0	±3.7	0.1%

Figure S59. Left: Raw (experimental) time-resolved photoluminescence decay of **6** in 2-MeTHF at 298 K ($λ_{exc}$ = 375 nm, $λ_{em}$ = 535 nm). Right: Fitting parameters and confidence limits.

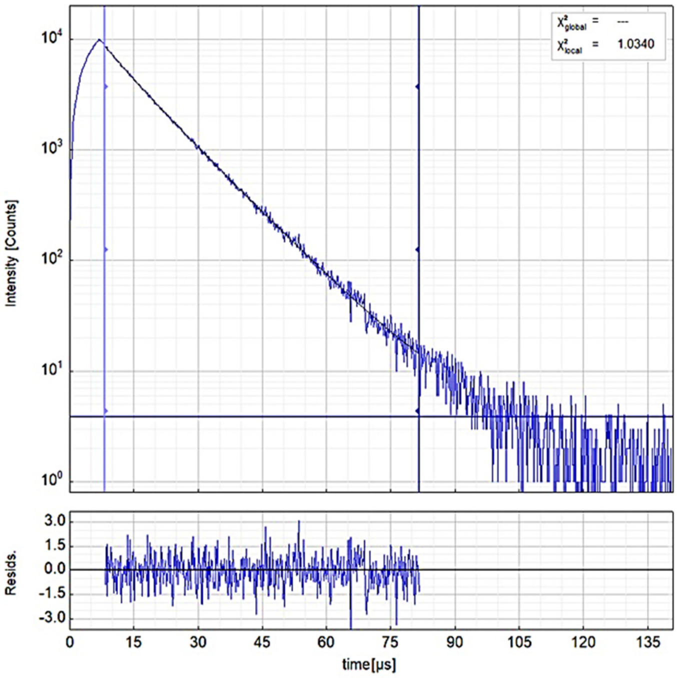

Parameter	Value	Δ	δ
A_1[kCnts/Chnl]	6.90	±0.36	5.1%
τ_1[ns]	11 310	±220	1.9%
I_1[kCnts]	488	±17	3.3%
A_2[kCnts/Chnl]	1.68	±0.36	22%
τ_2[ns]	5 710	±580	10%
I_2[kCnts]	60	±18	29%
$Bkgr_{Dec}$[kCnts]	0.0039	±0.0013	32%
τ_{AvInt}[ns]	10 698	±48	0.4%

Figure S60. Left: Raw (experimental) time-resolved photoluminescence decay of **6** in 2-MeTHF at 77 K (λ_{exc} = 375 nm, λ_{em} = 530 nm). Right: Fitting parameters and confidence limits.

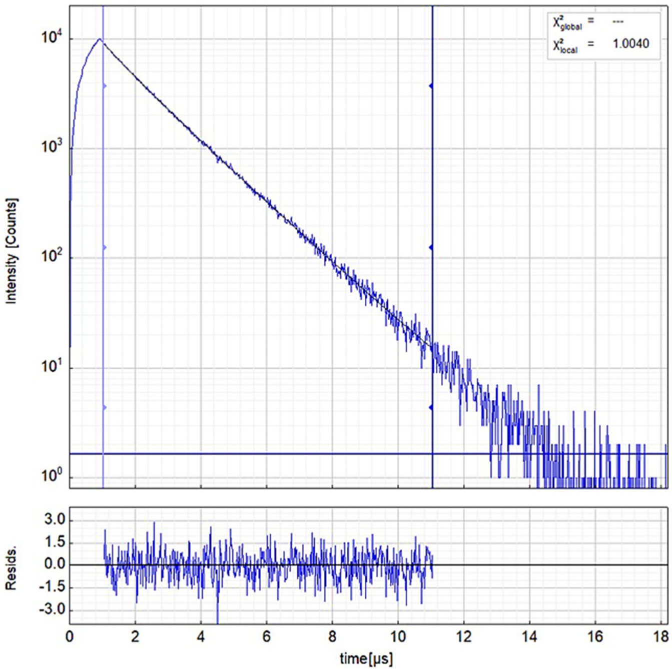

Parameter	Value	Δ	δ
A_1[kCnts/Chnl]	7.30	±0.50	6.8%
τ_1[ns]	1 585	±31	1.9%
I_1[kCnts]	578	±29	5.0%
A_2[kCnts/Chnl]	1.65	±0.47	28%
τ_2[ns]	760	±130	16%
I_2[kCnts]	63	±31	49%
$Bkgr_{Dec}$[kCnts]	0.0017	±0.0013	77%
τ_{AvInt}[ns]	1 504.1	±4.9	0.3%

Figure S61. Raw (experimental) time-resolved photoluminescence decay of **7** in PMMA film (5 wt%) at 298 K (λ_{exc} = 375 nm, λ_{em} = 545 nm), fitting parameters and confidence limits.

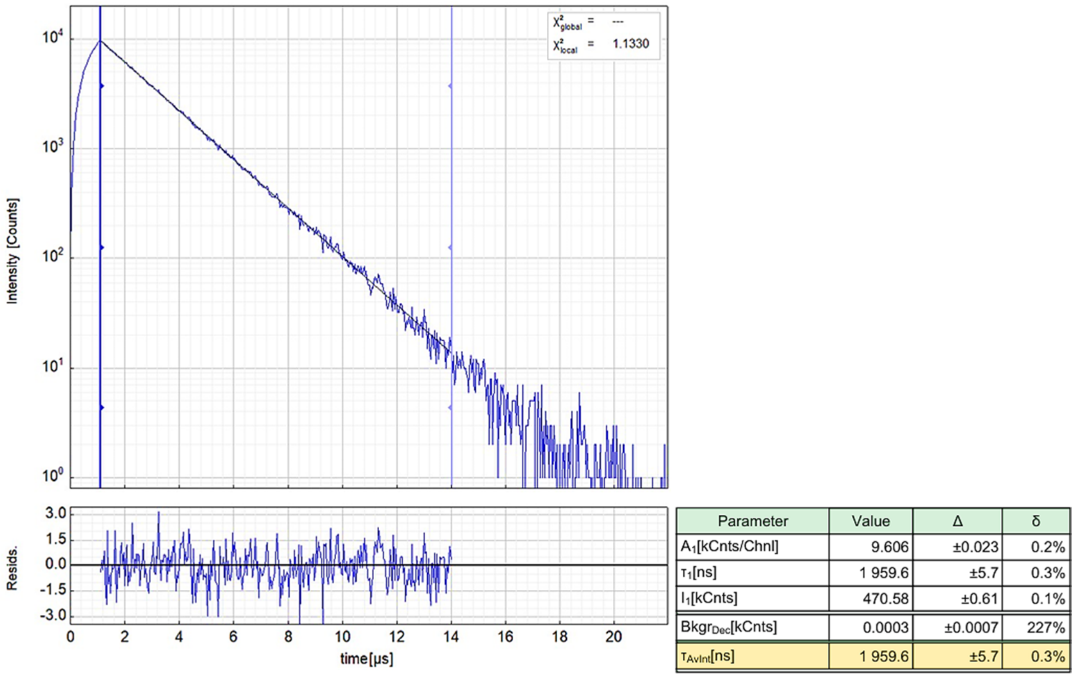

Parameter	Value	Δ	δ
A_1[kCnts/Chnl]	9.606	±0.023	0.2%
τ_1[ns]	1 959.6	±5.7	0.3%
I_1[kCnts]	470.58	±0.61	0.1%
$Bkgr_{Dec}$[kCnts]	0.0003	±0.0007	227%
τ_{AvInt}[ns]	1 959.6	±5.7	0.3%

Figure S62. Left: Raw (experimental) time-resolved photoluminescence decay of **7** in 2-MeTHF at 298 K (λ_{exc} = 375 nm, λ_{em} = 545 nm). Right: Fitting parameters and confidence limits.

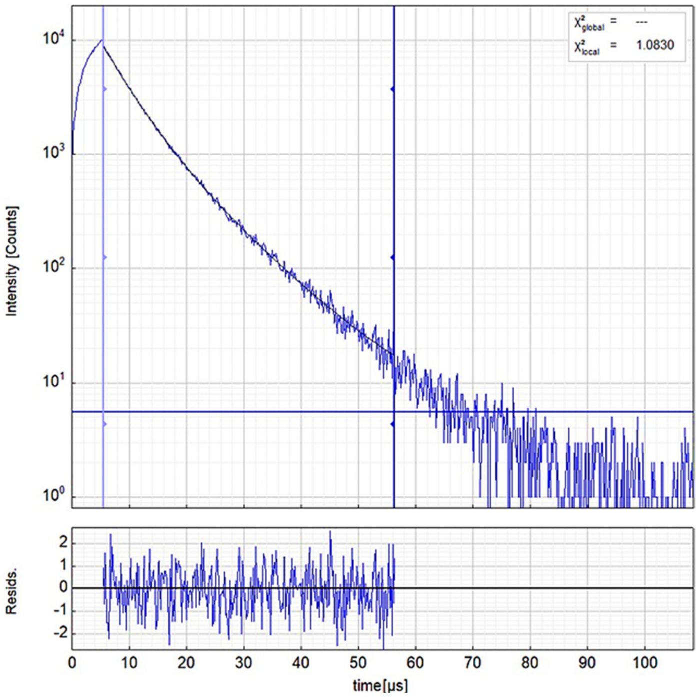

Parameter	Value	Δ	δ
A_1[kCnts/Chnl]	2.59	±0.35	13%
τ_1[ns]	9 430	±490	5.1%
I_1[kCnts]	153	±14	8.9%
A_2[kCnts/Chnl]	6.38	±0.32	4.9%
τ_2[ns]	4 280	±130	2.8%
I_2[kCnts]	171	±14	7.9%
$Bkgr_{Dec}$[kCnts]	0.0055	±0.0021	37%
τ_{AvInt}[ns]	6 708	±76	1.1%

Figure S63. Left: Raw (experimental) time-resolved photoluminescence decay of **7** in 2-MeTHF at 77 K (λ_{exc} = 375 nm, λ_{em} = 560 nm). Right: Fitting parameters and confidence limits.

TGA curves of complexes 2−7

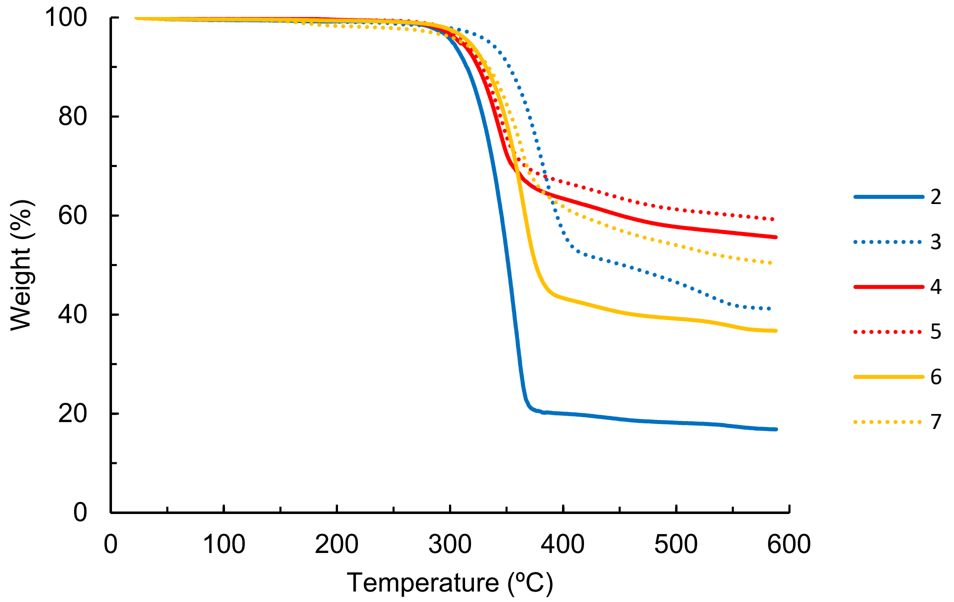

Figure S64. TGA curves of complexes **2−7**. The solids were heated under nitrogen (100 mL/min) at 10 °C/min.

References

(1) Blessing, R. H. *Acta Crystallogr.* **1995**, *A51*, 33. SADABS: Area-detector absorption correction; Bruker- AXS, Madison, WI, 1996.

(2) SHELXL-2016/6. Sheldrick, G. M. *Acta Cryst.* **2008**, *A64*, 112−122.

(3) (a) Lee, C.; Yang, W.; Parr, R. G. Development of the Colle-Salvetti correlation-energy formula into a functional of the electron density. *Phys. Rev. B* **1988**, *37*, 785–789. (b) Becke, A. D. Density-functional exchange-energy approximation with correct asymptotic behavior. *J. Chem. Phys*. **1993**, *98*, 5648–5652. (c) Stephens, P. J.; Devlin, F. J.; Chabalowski, C. F.; Frisch, M. J. Ab Initio Calculation of Vibrational Absorption and Circular Dichroism Spectra Using Density Functional Force Fields. *J. Phys. Chem*. **1994**, *98*, 11623–11627.

(4) Grimme, S.; Antony, J.; Ehrlich, S.; Krieg, H. A consistent and accurate ab initio parametrization of density functional dispersion correction (DFT-D) for the 94 elements H-Pu. *J. Chem. Phys*. **2010**, 132, 154104.

(5) Gaussian 09, Revision D.01, Frisch, M. J.; Trucks, G. W.; Schlegel H. B.; Scuseria, G. E.; Robb, M. A.; Cheeseman, J. R.; Scalmani, G.; Barone, V.; Mennucci, B.; Petersson, G. A.; Nakatsuji, H.; Caricato, M.; Li, X.; Hratchian, H. P.; Izmaylov, A. F.; Bloino, J.; Zheng, G.; Sonnenberg, J. L.; Hada, M.; Ehara, M.; Toyota, K.; Fukuda, R.; Hasegawa, J.; Ishida, M.; Nakajima, T.; Honda, Y.; Kitao, O.; Nakai, H.; Vreven, T.; Montgomery, J. A.; Peralta, Jr., J. E.; Ogliaro, F.; Bearpark, M.; Heyd, J. J.; Brothers, E.; Kudin, K. N.; Staroverov, V. N.; Keith, T.; Kobayashi, R.; Normand, J.; Raghavachari, K.; Rendell, A.; Burant, J. C.; Iyengar, S. S.; Tomasi, J.; Cossi, M.; Rega, N.; S43 Millam, J. M.; Klene, M.; Knox, J. E.; Cross, J. B.; Bakken, V.; Adamo, C.; Jaramillo, J.; Gomperts, R.; Stratmann, R. E.; Yazyev, O.; Austin, A. J.; Cammi, R.; Pomelli, C.; Ochterski, J. W.; Martin, R. L.; Morokuma, K.; Zakrzewski, V. G.; Voth, G.

A.; Salvador, P.; Dannenberg, J. J.; Dapprich, S.; Daniels, A. D.; Farkas, O.; Foresman, J. B.; Ortiz, J. V.; Cioslowski, J.; Fox, D. J. Gaussian, Inc., Wallingford CT, 2013.

(6) Andrea, D.; Häußermann, U. M.; Dolg, M.; Stoll, H.; Preuss, H. Energy-adjusted *ab initio* pseudopotentials for the second and third row transition elements. *Theor. Chim. Acta* **1990**, *77*, 123–141.

(7) Ehlers, A. W.; Bohme, M.; Dapprich, S.; Gobbi, A.; Hollwarth, A.; Jonas, V.; Kohler, K. F.; Stegmann, R.; Veldkamp, A.; Frenking, G. A set of f-polarization functions for pseudo-potential basis sets of the transition metals Sc-Cu, Y-Ag and La-Au. *Chem. Phys. Lett.* **1993**, *208*, 111–114.

(8) (a) Hehre, W. J.; Ditchfield, R.; Pople, J. A. Self-Consistent Molecular Orbital Methods. XII. Further Extensions of Gaussian-Type Basis Sets for Use in Molecular Orbital Studies of Organic Molecules. *J. Chem. Phys*. **1972**, *56*, 2257–2261. (b) Francl, M. M.; Pietro, W. J.; Hehre, W. J.; Binkley, J. S.; Gordon, M. S.; DeFrees, D. J.; Pople, J. A. Self-consistent molecular orbital methods. XXIII. A polarization-type basis set for second-row elements. *J. Chem. Phys*. **1982**, *77*, 3654–3665.

(9) Marenich, A. V.; Cramer, C. J.; Truhlar, D. G. Universal Solvation Model Based on Solute Electron Density and on a Continuum Model of the Solvent Defined by the Bulk Dielectric Constant and Atomic Surface Tensions. *J. Phys. Chem. B* **2009**, *113*, 6378–6396.

(10) O'Boyle, N. M.; Tenderholt, A. L.; Langner, K. M. cclib: A Library for Package-Independent Computational Chemistry Algorithms. *J. Comput. Chem.* **2008**, *29*, 839−845.
